# Succinate Dehydrogenase B (SDHB) Overexpression with Enzymatic Dysfunction Defines a Distinct Subtype of Undifferentiated Pleomorphic Sarcoma

**DOI:** 10.1158/2767-9764.CRC-25-0468

**Published:** 2025-10-30

**Authors:** Miguel Esperança-Martins, Hugo Vasques, Manuel Sokolov Ravasqueira, Filipa Santos, Filipa Fonseca, António Syder Queiroz, João Boavida, Daniel Martins Jordão, Joaquim Soares do Brito, Patrícia Corredeira, Marta Martins, Ângela Afonso, Jorge Antonio López, Richard S.P. Huang, Cecília Melo-Alvim, Isabel Fernandes, Dolores López-Presa, Maria Manuel Lemos, Brian A. Van Tine, Alliny C.S. Bastos, Sandra Casimiro, Nuno Abecasis, Luís Costa, Emanuel Gonçalves, Iola F. Duarte, Sérgio Dias

**Affiliations:** 1 Medical Oncology Department, Unidade Local de Saúde de Santa Maria, Lisboa, Portugal.; 2 Oncology Translational Laboratory, Gulbenkian Institute for Molecular Medicine, Lisboa, Portugal.; 3 Faculdade de Medicina da Universidade de Lisboa, Universidade de Lisboa, Lisboa, Portugal.; 4 General Surgery Department, Instituto Português de Oncologia de Lisboa Francisco Gentil, Lisboa, Portugal.; 5 Instituto de Engenharia de Sistemas e Computadores – Investigação e Desenvolvimento (INESC-ID), Lisboa, Portugal.; 6 Instituto Superior Técnico, Universidade de Lisboa, Lisboa, Portugal.; 7 Pathology Department, Instituto Português de Oncologia de Lisboa Francisco Gentil, Lisboa, Portugal.; 8 Pathology Department, Unidade Local de Saúde de Santa Maria, Lisboa, Portugal.; 9 General Surgery Department, Unidade Local de Saúde de Santa Maria, Lisboa, Portugal.; 10 Orthopedics Department, Unidade Local de Saúde de Santa Maria, Lisboa, Portugal.; 11 F.Hoffmann-LaRoche AG, Basel, Switzerland.; 12 Foundation Medicine Inc, Boston, Massachusetts.; 13 Medical Oncology Department, Hospital CUF Descobertas, Lisboa, Portugal.; 14 EpiDoC, CHRC, Nova Medical School, Universidade Nova de Lisboa, Lisboa, Portugal.; 15 Siteman Cancer Center, St. Louis, Missouri.; 16 Division of Oncology, Section of Medical Oncology, Washington University School of Medicine (WashU Medicine), St. Louis, Missouri.; 17 Department of Chemistry, LAQV-REQUIMTE & CICECO-Aveiro Institute of Materials, Universidade de Aveiro, Aveiro, Portugal.

## Abstract

Undifferentiated pleomorphic sarcoma (UPS) remains one of the most clinically aggressive and poorly characterized soft-tissue sarcoma subtypes. To uncover distinctive molecular traits for UPS, a multiomics analysis of UPS compared with leiomyosarcoma (LMS) and liposarcoma (LPS) was performed. Transcriptomic profiling revealed that UPS exhibits overexpression of genes encoding succinate dehydrogenase (SDH) subunits, particularly *SDHB*, *SDHC*, and *SDHD*, distinguishing it from LMS and dedifferentiated LPS. This finding was validated using The Cancer Genome Atlas Sarcoma dataset. High *SDHB* expression in UPS was significantly associated with shorter overall survival, shorter recurrence-free survival, and shorter overall survival from the date of first metastasis. IHC validated elevated SDHB protein levels in UPS and LMS relative to dedifferentiated LPS. Despite overexpression of SDH subunits, metabolomic profiling demonstrated a significantly higher succinate-to-fumarate ratio in UPS, suggesting functional impairment of SDH enzymatic activity potentially due to posttranslational modifications, altered assembly of SDH subunits, or imbalanced tricarboxylic acid flux. This paradoxical phenotype of SDH overexpression with enzymatic dysfunction defines a unique molecular and metabolic subtype of UPS with prognostic significance. Recognition of this distinct SDH-associated molecular and metabolic phenotype provides insights into UPS pathogenesis, identifies a potential novel prognostic biomarker, and suggests a new avenue for metabolic-targeted therapy.

**Significance:**

This study identified a paradoxical phenotype of SDH subunit overexpression with functional impairment in UPS, defining a molecular/metabolic subtype associated with poor prognosis. It offers new data on UPS pathogenesis and treatment.

## Introduction

Soft-tissue sarcomas (STS) are a group of rare and heterogeneous malignancies arising from mesenchymal tissues, accounting for approximately 1% of adult cancers and up to 15% of pediatric solid tumors (1). Their rarity, biological diversity, and heterogeneity, with more than 100 recognized histopathologic subtypes, pose significant challenges to understanding their molecular pathogenesis and to discovering and developing effective targeted therapies (2). Although subtype-specific genetic alterations have been identified in certain sarcomas, such as *KIT*/*PDGFRA* mutations in gastrointestinal stromal tumors or the *MDM2*/*CDK4* amplification in dedifferentiated liposarcomas (DDLPS), the majority of high-grade STS lack clearly defined and targetable molecular drivers (3). As a result, clinical outcomes for advanced or recurrent disease remain poor, and therapeutic advances have been limited for most of the high-grade sarcomas (4, 5).

Among adult STS, undifferentiated pleomorphic sarcoma (UPS) and L-type sarcomas [leiomyosarcoma (LMS) and liposarcoma (LPS)] are the three most prevalent histopathologic subtypes (6). UPS represents ∼17% of STS and has 5- and 10-year overall survival (OS) rates of approximately 60% and 48%, respectively, with high rates of local (14.1%) and distant (7.8%) recurrence (7–9). Despite its significant prevalence among STS and dismal prognosis, UPS remains poorly characterized at the molecular level outside of The Cancer Genome Atlas (TCGA; ref. 10). Previous studies have suggested similarities in gene expression and IHC profiles between UPS and LMS, suggesting that they share common oncogenic pathways and may correspond to different stages of the same oncologic entity (11–13), but these observations have not delineated a unique molecular or metabolic feature for UPS.

Recent evidence has highlighted the central role of tumor metabolism in driving oncogenesis, disease progression, and therapeutic resistance (14–18). Cancer cells undergo metabolic adaptations to meet the bioenergetic and biosynthetic demands of rapid proliferation (19). Mitochondrial enzymes, particularly those involved in the tricarboxylic acid (TCA) cycle and oxidative phosphorylation (OXPHOS), play a critical role in this adaptation (19, 20). Succinate dehydrogenase (SDH), also known as mitochondrial complex II, is at the interface of the TCA cycle and the electron transport chain (ETC), oxidizing succinate to fumarate while transferring electrons to ubiquinone (21). Inactivating mutations or epigenetic silencing of SDH subunits leading to succinate accumulation are well-established oncogenic mechanisms in several tumor types, including gastrointestinal stromal tumors, paragangliomas, and renal cell carcinomas (21, 22). In these contexts, SDH deficiency and succinate accumulation promote tumorigenesis through stabilization of hypoxia-inducible factors and widespread epigenetic dysregulation (23).

In contrast to these known SDH-deficient neoplasms, the role of SDH in UPS and other high-grade sarcomas has not been systematically investigated. Whether UPS harbors alterations in SDH expression or activity, and whether such alterations contribute to its aggressive clinical behavior, remains unknown. To address these gaps, the present study employed an integrative multiomics approach to define the molecular and metabolic biology of UPS. These findings provide evidence that SDH overexpression and enzymatic dysfunction in UPS has prognostic significance and potential therapeutic implications.

## Materials and Methods

### Gene expression analysis

#### Tissue cohort

The gene expression cohort consisted of 102 formalin-fixed, paraffin-embedded (FFPE) tumor samples from 101 patients with a high-grade (grade 3) STS, including 51 patients with a UPS, 25 patients with a high-grade LMS, and 25 patients with a DDLPS. Clinical, pathologic, and treatment characteristics have been described previously (24).

#### DNA and RNA sequencing

DNA and RNA were co-extracted from FFPE sections and analyzed at Foundation Medicine Inc., a Clinical Laboratory Improvement Amendments–certified, College of American Pathologists–accredited, and New York State–approved laboratory. DNA sequencing (DNA-seq) was performed using the FoundationOneCDx assay (F1CDx; ref. 25), and RNA sequencing (RNA-seq) was performed using the research-use–only FoundationOneRNA assay (F1RNA; ref. 26), as previously described (24).

#### Quality control

Of the 102 tumor samples (51 UPS, 25 LMS, and 26 DDLPS), 79 passed F1CDx quality control (QC), and 75 passed F1RNA QC. Samples failing F1RNA QC were predominantly archival, with a median age of 4.7 years. One additional outlier was excluded from the RNA-seq expression analysis based on principal component analysis. A total of 74 samples (43 UPS, 15 LMS, and 16 DDLPS) were included in downstream gene expression analyses.

#### RNA-seq–based differential gene expression analysis

Computational analyses were conducted in R v4.4.0 (RRID: SCR_001905). Lowly expressed genes were removed using the edgeR v4.2.1 (RRID: SCR_012802; ref. 27) filterByExpression method, followed by Voom normalization. Differential gene expression (DGE) analyses between histopathologic subtypes were performed using limma v3.60.4 (RRID: SCR_010943; ref. 28), and *P* values were adjusted for multiple testing with the Benjamini–Hochberg FDR. Genes with an adjusted *P* value ≤ 0.05 were considered differentially expressed. The concordance between metabolic pathways and genes included in F1RNA was identified using Kyoto Encyclopedia of Genes and Genomes pathway (RRID:SCR_018145) annotations (29).

#### Clinical significance of differentially expressed genes (study cohort)

As previously mentioned, 74 samples (43 UPS, 15 LMS, and 16 DDLPS) were considered for the downstream gene expression analyses. These 74 samples were obtained from 74 patients. Of these 74 patients, four had not been submitted to a surgical approach and were, therefore, excluded from the survival analyses, which means that a total of 70 patients were considered. Of the 70 patients whose samples were considered for the downstream gene expression analyses and that were considered for the survival analyses, 51 contributed with primary tumor samples to the study cohort, whereas 19 contributed with samples that corresponded to locally recurrent tumors (*n* = 13) or metastases (*n* = 6). Survival analyses were performed, within and between each histopathologic subtype and for each gene baited for expression and included in F1RNA, in R using the survival package v3.7.0 (30) for OS, recurrence-free survival (RFS), metastasis-free survival, progression-free survival, and OS from the date of metastasis. Samples were stratified into high- or low-expression groups for specific genes based on whether the gene expression levels were above or below the mean expression of the gene. Log-rank tests were then applied for each gene. Overrepresentation analysis (ORA; ref. 31) was conducted on differentially expressed genes (DEG), stratified by the directionality of expression (over- or under-expressed). This ORA was performed for sets of gene for which expression was significantly associated with survival (log-rank *P* < 0.05), further dividing by favorable versus unfavorable prognostic associations.

#### External validation using TCGA Sarcoma (independent cohort)

The TCGA Sarcoma (TCGA-SARC) dataset (RRID: SCR_003193; ref. 32) was used as an independent validation cohort, filtered to only include UPS, LMS, and DDLPS samples based on the updated histopathologic classification. RNA-seq data were processed and analyzed and survival analysis and ORA were performed similarly to which was done for the study cohort.

To assess concordance between cohorts, we evaluated whether the direction of expression changes between histopathologic subtypes was consistent across cohorts. For each pairwise comparison (UPS vs. LMS, UPS vs. DDLPS, and LMS vs. DDLPS), we identified genes with an adjusted *P* value < 0.05 in both cohorts and intersected these sets. Within the overlapping gene set, we then compared the direction of expression changes by analyzing log fold-change (log FC) values to determine whether genes were consistently overexpressed (log FC > 0) or under-expressed (log FC < 0) in both datasets. Statistical significance of the directional concordance was assessed using a hypergeometric test. In addition to DGE concordance, we investigated the consistency of survival associations. Focusing on the UPS subtype, we selected genes significantly associated with OS, defined by a log-rank test *P* value < 0.05, in each cohort. For each gene, we determined whether higher expression was associated with a favorable or unfavorable prognosis. We then intersected the sets of significant genes from the study cohort and TCGA and compared the directionality of their survival associations—i.e., whether high expression was consistently linked to either improved or worsened survival across datasets. This concordance was also evaluated using a hypergeometric test, following the same logic as in the DGE comparison.

### IHC evaluation of SDHB protein levels

All FFPE tumor sections were originally submitted to histopathologic review by a sarcoma pathologist at the Pathology Department of Instituto Português de Oncologia de Lisboa Francisco Gentil (IPOLFG). All cases were fixed in 10% formalin. SDHB IHC was performed on deparaffinized sections using a standard avidin–biotin–peroxidase complex method in an automated platform (BenchMark ULTRA, Ventana). FFPE blocks were qualitatively analyzed for the expression of a rabbit monoclonal anti-SDHB antibody (clone EP288, ref MAD-000739QD, Vitro Master Diagnóstica). Gastrointestinal stromal tumor tissue sections served as positive and negative controls. Two pathologists, blinded to clinical, pathologic, and molecular data, scored staining intensity. Definite cytoplasmic granular staining was considered positive. Immunostaining intensity was classified using a newly developed three-tier system: score 1: focal and weak staining (<10% of cells); score 2: diffuse and intermediate staining (10%–90% of cells); and score 3: diffuse and strong staining (>90% of cells). Additionally, cases lacking unequivocal cytoplasmic granular staining were considered negative and cases lacking a definite positive internal control were considered non-informative.

### Nuclear magnetic resonance metabolomics

#### Tissue cohort

Fresh frozen tumor and normal tissue samples were collected from 16 patients with STS undergoing surgery between 2021 and 2023 at two Portuguese sarcoma centers: IPOLFG and Unidade Local de Saúde de Santa Maria. Tumor samples (*n* = 15) included three UPS, three LMS, two LPS (one DDLPS and one myxoid LPS), and additional rare sarcomas (dermatofibrosarcoma protuberans, solitary fibrous tumor, synovial sarcoma, chondrosarcoma, clear-cell sarcoma–like tumor of the gastrointestinal tract, endometrial stromal sarcoma, and adamantinoma). Histopathologic diagnoses were confirmed by a dedicated sarcoma pathologist at IPOLFG. Clinical data were obtained from institutional records and national electronic health records. Data were anonymized and follow-up was closed on April 21, 2025.

#### Metabolite extraction from fresh tissue

Samples were first macerated using an agate mortar and pestle under liquid nitrogen and then stored at –80°C. Metabolites were extracted from ∼50 mg tissue by sequential addition of 650 µL of cold methanol 80%, 520 µL of cold chloroform, and 260 µL of cold Milli-Q water, followed by vortexing for 1 minute after each addition. After the final addition of water and vortexing, samples were incubated at 4°C for 20 minutes and centrifuged at 3,000 ×*g* for 10 minutes. A 500 µL aliquot of the resulting upper aqueous phase was collected, dried in a speed vacuum concentrator (Centrivap, model 73100, Labconco), and stored at –80°C until further analysis.

#### ^1^H nuclear magnetic resonance data acquisition and analysis

Dried extracts were reconstituted in deuterated phosphate buffer (pH 7.4) with 0.1 mmol/L TSP-d4 and transferred to 3-mm nuclear magnetic resonance tubes. Spectra were acquired at 298 K on a Bruker Avance III HD 500 MHz spectrometer using a standard noesypr1d sequence with water suppression, 512 scans, and processed in TopSpin 4.0.3 (RRID: SCR_014227). 2D ^1^H–^1^H TOCSY and J-resolved spectra were acquired for selected samples. Metabolite identification was based on Chenomx (RRID: SCR_014682) and BBIOREFCODE reference libraries.

Spectral integration and total area normalization were performed in Amix Viewer 3.9.15 (RRID: SCR_025468). Data normality was assessed using the Shapiro–Wilk test. For comparisons among three groups, one-way ANOVA with Tukey *post hoc* test (parametric) or Kruskal–Wallis with Dunn *post hoc* test (non-parametric) was used. Two-group comparisons were made using an unpaired Student *t* test with Welch correction or the Mann–Whitney test, as appropriate. A *P* value < 0.05 was considered statistically significant.

## Results

### Clinical and pathologic characteristics of the study cohort

From 101 patients with STS (51 UPS, 25 high-grade LMS, and 26 DDLPS), 102 FFPE tumor samples were studied (Table 1). One patient contributed with two independent samples. The cohort included mostly primary high-grade STS from diverse anatomic sites, representing the topographical heterogeneity of high-grade STS.

**Table 1.** Demographic, pathologic, clinical, and treatment characteristics of the study cohort that was considered for the gene expression profiling evaluation.

a. Demographic, pathologic, and clinical characteristics of the study cohort	
​	Total (*n* = 101)
Age at diagnosis, median (IQR), years	67 (19.7)
Gender, *n* (%)	​
Male	50 (49.5)
Female	51 (50.5)
Histopathologic subtypes, *n* (%)	​
DDLPS	25 (24.8)
LMS	25 (24.8)
UPS	51 (50.4)
Location, *n* (%)	​
Upper limb	9 (8.9)
Lower limb	49 (48.5)
Retroperitoneum	31 (30.7)
Trunk	12 (11.9)
Presentation type, *n* (%)	​
Localized	96 (95.0)
Distant metastasis	5 (5.0)
Size of the primary tumor, median (IQR), cm	13 (10.0)
Missing	4

b. Surgical and systemic treatment details of the patients of the study cohort	
​	Total (*n* = 94)
Neoadjuvant treatment, *n* (%), *n* = 94	​
No neoadjuvant treatment	91 (96.8)
Neoadjuvant treatment	3 (3.2)
Resectability, *n* (%), *n* = 94	​
Resectable	92 (97.9)
Unresectable	2 (2.1)
Resection margins, *n* (%), *n* = 92	​
R0/R1	89 (96.7)
R2	3 (3.3)
Adjuvant treatment, *n* (%), *n* = 92	​
Radiotherapy	55 (59.7)
No adjuvant treatment	34 (37.0)
Chemotherapy or chemoradiotherapy	3 (3.3)

### UPS displays distinct metabolic gene signatures

DGE analysis revealed that UPS display a closer gene expression pattern to high-grade LMS than to DDLPS (Supplementary Fig. S1). The lower number of DEGs between UPS and high-grade LMS (67 DEGs; Supplementary Table S1) compared with UPS and DDLPS (241 DEGs; Supplementary Table S2), as well as the smaller magnitude of DGE, with a mean log FC of 0.009 for UPS versus high-grade LMS and 0.01 for UPS versus DDLPS, supports this finding.

To identify features specific to UPS, we examined DEGs and pathways, either globally and across metabolism-related genes. Although the F1RNA gene panel contained limited representation of metabolic genes (84 of 1,519; Supplementary Fig. S2; Supplementary Table S3), three TCA cycle– and OXPHOS-related genes—*SDHB*, *SDHC*, and *SDHD*—and one arachidonic acid metabolism–related gene—*ALOX12*—were found to be significantly overexpressed in UPS relatively to both LMS and also to DDLPS (Supplementary Figs. S3 and S4; Supplementary Table S4).

### *SDHB*, *SDHC*, and *SDHD* are overexpressed in UPS relatively to high-grade LMS and DDLPS

**Figure 1.**
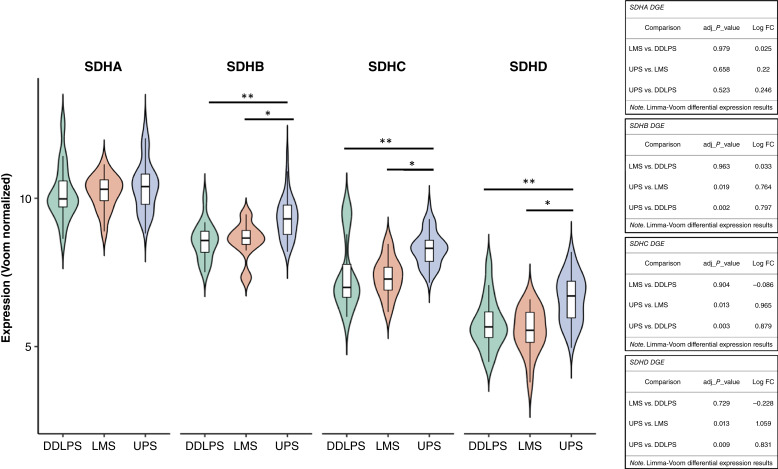
Limma-Voom differential expression of SDHA, SDHB, SDHC, and SDHD for comparative evaluations between UPS, high-grade LMS, and DDLPS. For each SDH DGE analysis (between different high-grade STS subtypes), adjusted *P* values and log FC values are provided. SDHB, SDHC, and SDHD are all significantly overexpressed in UPS vs. high-grade LMS (adjusted *P* value < 0.05) and in UPS vs. DDLPS (adjusted *P* value < 0.05). *, *P* value < 0.05; **, *P* value < 0.01.

Limma-Voom differential expression analysis confirmed that *SDHB*, *SDHC*, and *SDHD* were significantly overexpressed in UPS in comparison with both LMS and DDLPS, displaying positive log FC values and significant adjusted *P* values (Fig. 1). These genes encode subunits of the SDH complex, which bridges TCA cycle flux and ETC (linking the TCA cycle and OXPHOS). These results suggest that mitochondrial activity is relatively upregulated in UPS.

Size, topographical location, employment of adjuvant treatment, and sex did not show any correlation with *SDHA*, *SDHB*, *SDHC*, and *SDHD* expression patterns in the specific UPS population (Supplementary Fig. S5).

### *SDHB* overexpression is associated with poor survival in UPS

**Figure 2.**
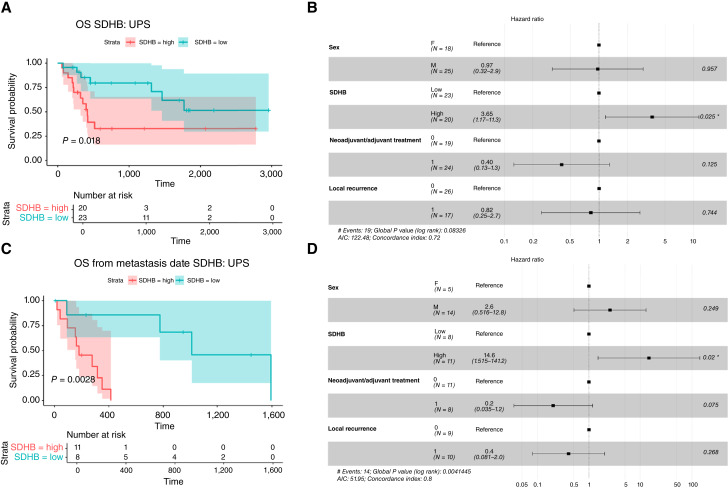
Within the UPS population, overexpression of SDHB is significantly correlated with shorter OS, using a Kaplan–Meier model (**A,***P* value < 0.05) and also using a Cox proportional hazards model (**B,***P* value < 0.05), and also with a shorter OS from the date of first metastasis, using a Kaplan–Meier model (**C,***P* value < 0.05) and also using a Cox proportional hazards model (**D,***P* value < 0.05). *, *P* value < 0.05.

*SDH* gene overexpression is correlated with clinical outcomes in UPS. High *SDHB* expression was significantly correlated with shorter OS and shorter OS from the date of first metastasis in UPS, as shown by both Kaplan–Meier (Fig. 2A and C) and Cox proportional hazards models (Fig. 2B and D). High *SDHB* expression was also significantly correlated with shorter RFS in a Kaplan–Meier model–based analysis (Supplementary Fig. S6A) although this correlation was found not to be statistically significant in a Cox proportional hazards model (Supplementary Fig. S6B). The absence of a profound statistical significance of the association in the Kaplan–Meier–based analysis (*P* value 0.044) and the inclusion of neoadjuvant/adjuvant treatment (which affects RFS) in the Cox proportional hazards model may explain this discrepancy. Moreover, for this specific UPS population, *SDHB* overexpression was not significantly associated with either metastasis-free survival or progression-free survival although trends toward poorer outcomes were observed (Supplementary Fig. S7). *SDHC* and *SDHD* overexpression showed no significant associations with either OS or other survival endpoints in UPS (Supplementary Figs. S8 and S9). *SDHB*, *SDHC*, and *SDHD* overexpression did not show any significant association with any survival endpoint in the whole cohort population (Supplementary Figs. S10–S13).

### TCGA-SARC validates UPS–LMS similarity and reveals UPS-specific pathway enrichment

In the independent TCGA-SARC cohort, UPS again demonstrated greater transcriptomic similarity to LMS than to DDLPS, with fewer DEGs and lower log FC between UPS and LMS (Supplementary Fig. S14; Supplementary Tables S5 and S6). Gene set enrichment analysis and differential pathway expression analysis confirmed the significant upregulation of OXPHOS and pyrimidine metabolism in UPS compared with LMS (Supplementary Fig. S15). Additional enrichment in immune activation–related pathways, such as antigen processing and presentation, and diverse DNA damage repair pathways/mechanisms was also observed in UPS. Pathway analysis reinforced the overexpression of diverse mitochondrial components and pathways, including SDH (complex II) and different respiratory chain and ATP synthesis pathways, in UPS relatively to LMS (Supplementary Fig. S16).

### TCGA-SARC validates *SDHB* overexpression in UPS relatively to high-grade LMS and DDLPS

**Figure 3.**
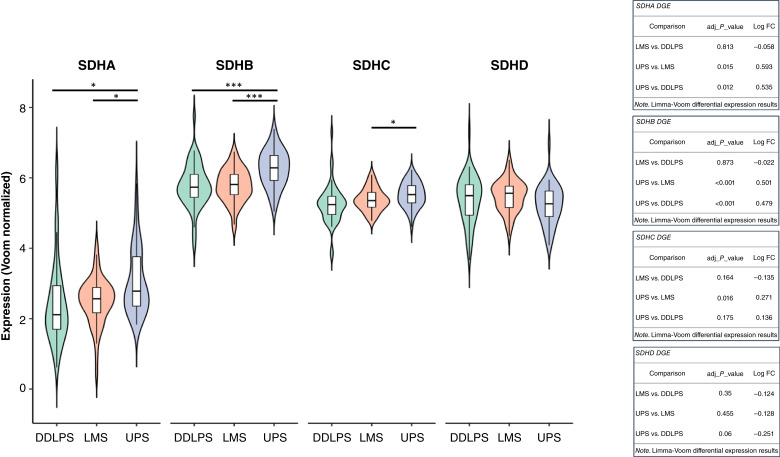
Limma-Voom differential expression of SDHA, SDHB, SDHC, and SDHD for comparative evaluations between UPS, high-grade LMS, and DDLPS samples of an independent cohort (TCGA-SARC). For each SDH DGE analysis (between different high-grade STS subtypes), adjusted *P* values and log FC values are provided. SDHA and SDHB are significantly overexpressed in UPS vs. high-grade LMS (adjusted *P* value < 0.05) and in UPS vs. DDLPS (adjusted *P* value < 0.05), whereas SDHC is only significantly overexpressed in UPS vs. high-grade LMS (adjusted *P* value < 0.05). *, *P* value < 0.05; **, *P* value < 0.01; ***, *P* value < 0.001.

We evaluated whether the directionality of gene expression changes was conserved across both datasets. For each pairwise, we identified genes significantly differentially expressed in both cohorts and compared their log FC values to determine directional concordance (Supplementary Figs. S17–S19). This directional agreement was statistically significant in all comparisons based on *χ*^2^ tests (Supplementary Fig. S20). Next, we performed similar DGE analyses comparing UPS with high-grade LMS and with DDLPS samples from the TCGA-SARC cohort. In both comparisons, *SDHA* and *SDHB* were found to be differentially expressed. Notably, the differential expression of *SDHB* between UPS and both high-grade LMS and DDLPS was consistently observed in both the study cohort and the independent TCGA-SARC dataset. Limma-Voom differential expression analysis of *SDHA*, *SDHB*, *SDHC*, and *SDHD* was performed for UPS versus high-grade LMS versus DDLPS (Fig. 3). *SDHA* differential overexpression in UPS relatively to both high-grade LMS and DDLPS was found to be statistically significant. Similarly, *SDHB* differential overexpression in UPS relatively to both high-grade LMS and DDLPS was also found to be statistically significant. These results validate *SDHB* overexpression as a distinctive molecular feature of UPS.

### The associations of *SDHB* overexpression with survival endpoints in UPS could not be validated using TCGA-SARC

We attempted to validate the associations of *SDHB* overexpression with survival endpoints that have been observed in the study cohort in UPS. We used the TCGA-SARC cohort for this purpose.

We examined whether the direction of the association between gene expression and OS was consistent across both cohorts. For this purpose, we identified genes for which expression was significantly associated with OS in each cohort and determined whether their high expression predicted better or worse prognosis. The overlap of significant genes was then used to evaluate whether the direction of survival association was conserved. For the UPS-only analysis, the *χ*^2^ test assessing survival direction concordance between both cohorts yielded a nonsignificant result (*P* value = 0.63; Supplementary Fig. S21). When the UPS, DDLPS, and LMS samples were considered, the *χ*^2^ test also yielded a nonsignificant result (*P* value = 0.71; Supplementary Fig. S22). This indicates that despite DGE concordance, survival associations do not seem to exhibit consistent directional agreement between cohorts.

We attempted to explore and examine potential causes of this discrepancy. An association between an enrichment in particular immune-related pathways (B cell– and humoral-dependent immune pathways) and high expression levels of genes for which expression is correlated with improved prognosis within this universe of UPS samples was verified in the TCGA-SARC cohort (Supplementary Fig. S23) but was not observed in our study cohort (Supplementary Fig. S24) despite the coverage of the same immune-related pathways by the F1RNA gene set (Supplementary Fig. S25). Interestingly, it is known that indels (generally) and frameshift indels (more specifically) can serve as potent generators of highly immunogenic neoantigens, leading to increased immunogenicity and activation of humoral immune–related transcriptional programs (33). In the study cohort, UPS did not exhibit either a higher general indel burden (Supplementary Fig. S26) and did not show a higher specific frameshift indel burden (Supplementary Fig. S27) compared with LMS or DDLPS. On the other hand, in the TCGA-SARC cohort, UPS samples displayed a markedly higher number of indels per patient relatively to the other two STS histotypes (Supplementary Fig. S28), whereas no significant differences in terms of frameshift indels were found between UPS, LMS, and DDLPS (even though UPS displayed a higher absolute number of frameshift indels when compared with LMS and DDLPS; Supplementary Fig. S29). It is important to note that within our study cohort, we employed the F1CDx assay, which utilizes targeted sequencing, whereas TCGA-SARC relies on whole-exome sequencing, which makes the absolute number and distribution of the detected indels non-directly comparable between cohorts. It is, indeed, possible that immunologically distinct features, likely driven by higher indel loads, influence survival patterns in the TCGA-SARC UPS cohort, limiting its utility for validating prognostic gene expression signatures identified in independent datasets. Moreover, the reduced size of the UPS sample pool (*n* = 43 in the study cohort and *n* = 44 in the TCGA-SARC cohort) of each cohort and differences in the composition of the gene sets employed for DNA-seq in each cohort may also explain this discrepancy.

### SDHB protein is overexpressed in UPS and LMS relative to DDLPS

**Figure 4.**
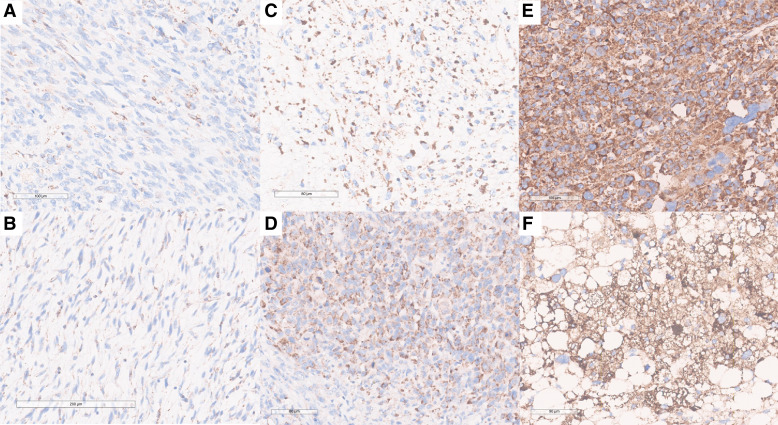
SDHB IHC in sarcomas: Three-tier score for SDH immunostaining intensity. **A** and **B** with weak and focal granular cytoplasmic immunostaining (score 1); **C** and **D** with diffuse intermediate granular cytoplasmic immunostaining (score 2); and **E** and **F** with diffuse and strong granular cytoplasmic immunostaining (score 3).

**Figure 5.**
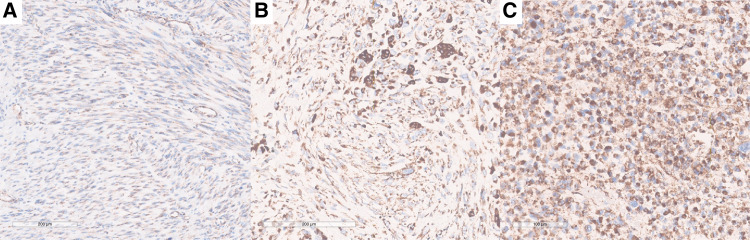
SDHB IHC in sarcomas: Differences in immunostaining intensity related to tumoral heterogeneity and cell morphology. Spindle cells with scarce and ill-defined cytoplasm tend to show a more focal and weak staining (**A**); epithelioid and pleomorphic cells, as well as multinucleated giant cells, with a more voluminous cytoplasm tend to show more granularity and diffusely intense staining (**B** and **C**).

IHC staining of SDHB was performed on FFPE samples, and a novel three-tier scoring system was applied (Fig. 4). UPS and LMS samples showed higher SDHB staining intensity than DDLPS. Specifically, 43% of UPS and 64% of LMS samples exhibited strong (score 3) staining compared with 12% of DDLPS (Fig. 5). Weak (score 1) staining was observed in 42% of DDLPS but was rare in UPS (6%) and absent in LMS. These findings confirm SDHB overexpression at the protein level in UPS and LMS.

### Metabolomic profiling reveals distinct sarcoma subtype–specific metabolic signatures

To assess whether these molecular differences translated to metabolic phenotypes, we performed ^1^H nuclear magnetic resonance metabolomics on fresh tumor tissue from patients with UPS (*n* = 3), LMS (*n* = 3), and LPS (*n* = 2). Detailed demographic, pathologic, surgical, and systemic treatment specificities of the 16 patients from whom normal and tumor tissue samples were collected are provided in Supplementary Tables S7 and S8.

**Figure 6.**
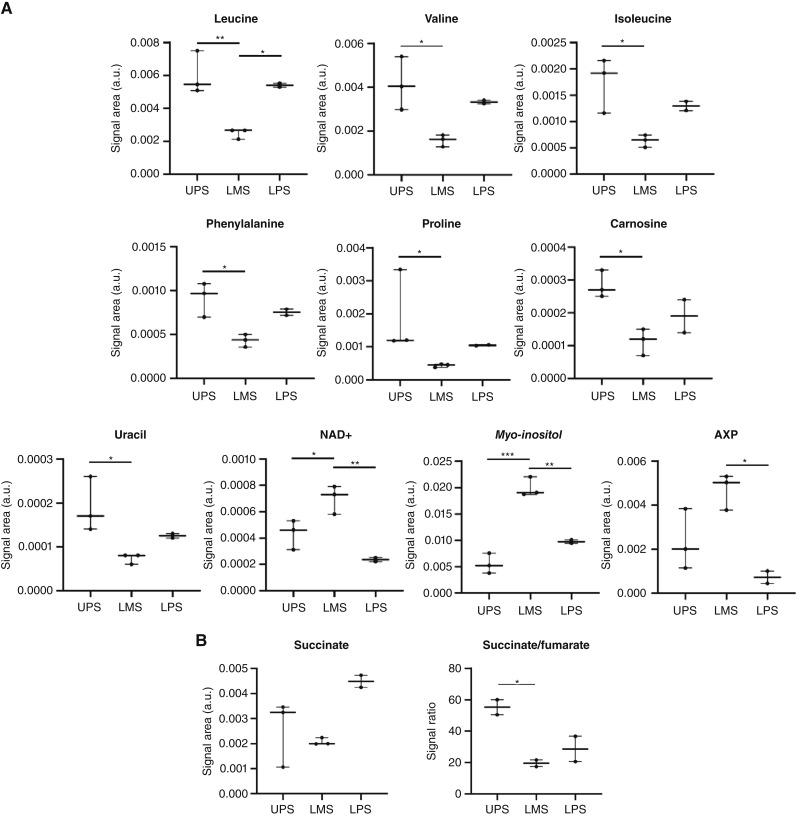
**A,** Metabolites for which abundance was significantly different between at least two of the three STS subtypes. **B,** Succinate levels and succinate-to-fumarate ratio in the different STS subtypes. Succinate-to-fumarate ratio is significantly higher in UPS vs. LMS and vs. LPS. *, *P* value < 0.05; **, *P* value < 0.01; ***, *P* value < 0.001. a.u., arbitrary units.

Forty metabolites were identified, with 10 differing significantly between subtypes (Fig. 6A). UPS displayed higher levels of branched-chain amino acids (leucine, isoleucine, and valine), phenylalanine, proline, carnosine, and uracil relative to LMS, consistent with increased protein turnover, anabolic growth, and oxidative stress adaptations. In contrast, LMS showed higher myo-inositol, NAD^+^, and adenine nucleotides, reflecting smooth muscle–specific metabolic programs. Although absolute succinate and fumarate levels did not differ significantly, the succinate-to-fumarate ratio was significantly higher in UPS compared with both LMS and LPS (Fig. 6B), demonstrating impaired SDH enzymatic activity despite overexpression of SDH subunits.

## Discussion

The identification of molecular and biological traits that distinguish UPS from high-grade LMS and DDLPS has not been deeply explored. Isolated single-omics studies, with limited sample sizes, compared UPS and LMS and highlighted a handful of differentially expressed microRNAs that target either UPS- and LMS-related genes (12) and *SRC* as potential discriminators (13), but these small studies did not robustly separate these two histopathologic subtypes at the transcriptomic level. Our study is the first to put the scope on a direct comparative analysis of the molecular profiles of UPS, LMS, and DDLPS employing a multiomics approach.

The gene expression profiling of the high-grade STS samples included in the study cohort points toward a closer molecular similarity between UPS and high-grade LMS, a finding that was validated in the TCGA-SARC dataset and that is consistent with prior descriptions (34).

UPS are distinctively characterized by the overexpression of TCA cycle– and OXPHOS-related genes, namely *SDHB*, *SDHC*, and *SDHD*, a finding that was also validated in the TCGA-SARC dataset. Although increased mitochondrial activity and TCA cycle upregulation have been previously reported in UPS (35, 36), the overexpression of SDH subunit–encoding genes represents a novel observation.

SDH, positioned at the interface of the TCA cycle and the ETC, is typically portrayed by a panoply of loss-of-function mutations in sarcomas, which promote succinate accumulation, epigenetic dysregulation, and oncogenesis (21, 22, 37). Overexpression of SDH subunits, in contrast, has been rarely described in cancer although elevated *SDHA* expression levels have been observed in metastatic uveal melanoma (37), advanced ovarian cancer (38), and multiple myeloma (39), in which they are linked to enhanced OXPHOS capacity, increased expression of proteins that shuttle electrons between different complexes of the ETC, augmented levels of key TCA enzymes, enhanced ATP yield, and amplified metabolic plasticity (37, 38). Although *SDHA* encodes a flavoprotein, *SDHB* encodes the iron–sulfur protein that functions as the SDH catalytic core (38). Given its role as the catalytic core of the SDH complex, *SDHB* overexpression may be linked to its dysfunction in UPS.

The correlation between high *SDHB* expression and poor survival in UPS in the study cohort further underscores the clinical relevance of this metabolic phenotype. Indeed, *SDHB* overexpression seems to identify a biologically aggressive subgroup of UPS, characterized by short survival rates, which may be little affected by the employment of different types of treatment. Survival associations could not be validated using TCGA-SARC, possibly because of either the small size of the UPS sample pool of each cohort and different coverage of the gene sets employed for DNA-seq in each cohort or potentially because of the putative survival impact of distinct immunologic profiles of UPS populations of each cohort, likely driven by higher relative indel burden and overexpression of antigen processing and presentation pathways of UPS samples of the TCGA-SARC dataset.

IHC evaluation confirmed higher SDHB protein abundance in UPS and LMS compared with DDLPS. The proposed three-tier scoring system for SDHB staining provides a newly developed tool to categorize SDH expression levels in sarcoma tissues, complementing transcriptomic findings and offering a potential biomarker for future studies. Nevertheless, it is important to note that intra-tumoral heterogeneity and cell morphologic patterns may influence the evaluation of granular staining intensity, potentially without any correlation with underlying genomic and/or transcriptomic alterations. This is particularly relevant for UPS, which may show areas with different morphologies mixing epithelioid, rhabdoid, pleomorphic, and spindle cells. SDH granules are in the cytoplasm. Epithelioid, rhabdoid, and pleomorphic cells tend to have a more voluminous cytoplasm and, therefore, effectively more granules are more easily detectable. Hence, staining tends to seem more intense and diffused in regions rich in these cells. Spindle cells, on the other hand, have a scarce and ill-defined cytoplasm, typically showing fewer granularities. This way, staining tends to seem less intense in regions rich in spindle cells.

Metabolomic profiling further revealed distinctive metabolic features of UPS, including an enrichment in amino acids, uracil, and carnosine. Increased amino acid levels may reflect enhanced protein turnover, anabolic growth, or altered transamination, consistent with aggressive tumor behavior (40). Higher uracil levels suggest augmented nucleotide turnover or dysregulated pyrimidine metabolism (41), in line with enhanced metabolic plasticity and proliferative signaling, whereas increased levels of carnosine, a dipeptide with antioxidant and pH-buffering properties (42), may indicate oxidative and metabolic stress adaptation. Carnosine accumulation could also contribute to immune evasion by modulating the expression of immune-modulatory proteins, protecting the neoplastic tissue from T cell–mediated immune surveillance (43). Together, these findings seem to locate critical metabolic adaptations (with clinical impact) of aggressive and progressing UPS in the mitochondria, potentially at the ETC.

The higher succinate-to-fumarate ratio observed in UPS, despite SDHB overexpression, suggests functional impairment of SDH enzymatic activity, potentially because of posttranslational modifications, SDH subunit misassemble, or TCA cycle flux imbalance. Future work in this direction will identify the posttranslational modifications that could be responsible for this observation and the implications of this phenotype on UPS microenvironmental composition and on the nature of antitumor (UPS) immune response.

Indeed, succinate accumulation was shown to increase antigen presentation by the transcriptional and epigenetic activation of MHC-APP–related genes in melanoma, potentially enhancing tumor immunogenicity and indirectly modifying the microenvironmental immune composition (44). The clarification of the interplay between SDH dysfunction, succinate accumulation, and immunogenicity specifically in UPS is thus crucial as the microenvironmental immune composition of UPS was shown to have prognostic value (denser infiltration by CD8^+^ T lymphocytes and monocytes is associated with significantly better survival outcomes; refs. 45, 46), and the intracellular succinate levels of both tumor cells and microenvironmental CD8^+^ T cells are known to be linked to different profiles of antitumor immunogenicity (47).

This study identified a paradoxical phenotype of SDH subunit overexpression with functional impairment in UPS, defining a molecular and metabolic subtype associated with poor prognosis. These findings suggest novel avenues for understanding UPS pathogenesis and for developing therapies targeting mitochondrial metabolism and its interaction with the immune microenvironment. Future studies should clarify the mechanisms underlying SDH dysfunction in UPS and explore the therapeutic potential of modulating SDH activity and succinate levels to improve clinical outcomes.

## Supplementary Material

Graphical Abstract Graphical abstract

Supplementary Results Characterization of the cohort of STS patients whose samples were used for the exploratory metabolomic characterization using 1H NMR

Supplementary Figure 1 Supplementary Figure 1

Supplementary Figure 2 Supplementary Figure 2

Supplementary Figure 3 Supplementary Figure 3

Supplementary Figure 4 Supplementary Figure 4

Supplementary Figure 5 Supplementary Figure 5

Supplementary Figure 6 Supplementary Figure 6

Supplementary Figure 7 Supplementary Figure 7

Supplementary Figure 8 Supplementary Figure 8

Supplementary Figure 9 Supplementary Figure 9

Supplementary Figure 10 Supplementary Figure 10

Supplementary Figure 11 Supplementary Figure 11

Supplementary Figure 12 Supplementary Figure 12

Supplementary Figure 13 Supplementary Figure 13

Supplementary Figure 14 Supplementary Figure 14

Supplementary Figure 15 Supplementary Figure 15

Supplementary Figure 16 Supplementary Figure 16

Supplementary Figure 17 Supplementary Figure 17

Supplementary Figure 18 Supplementary Figure 18

Supplementary Figure 19 Supplementary Figure 19

Supplementary Figure 20 Supplementary Figure 20

Supplementary Figure 21 Supplementary Figure 21

Supplementary Figure 22 Supplementary Figure 22

Supplementary Figure 23 Supplementary Figure 23

Supplementary Figure 24 Supplementary Figure 24

Supplementary Figure 25 Supplementary Figure 25

Supplementary Figure 26 Supplementary Figure 26

Supplementary Figure 27 Supplementary Figure 27

Supplementary Figure 28 Supplementary Figure 28

Supplementary Figure 29 Supplementary Figure 29

Supplementary Table 1 Supplementary Table 1

Supplementary Table 2 Supplementary Table 2

Supplementary Table 3 Supplementary Table 3

Supplementary Table 4 Supplementary Table 4

Supplementary Table 5 Supplementary Table 5

Supplementary Table 6 Supplementary Table 6

Supplementary Table 7 Supplementary Table 7

Supplementary Table 8 Supplementary Table 8

## Data Availability

The data generated in this study are publicly available in Figshare at https://figshare.com/s/7b32f477f01a1fcf7de0.
