# Supplementary figures and images for: Succinate Dehydrogenase B (SDHB) Overexpression with Enzymatic Dysfunction Defines a Distinct Subtype of Undifferentiated Pleomorphic Sarcoma
Source: Cancer Res Commun. 2025 Oct 30;5(10):1934–45. doi: 10.1158/2767-9764.CRC-25-0468 (PMC12573234; doi:10.1158/2767-9764.CRC-25-0468)

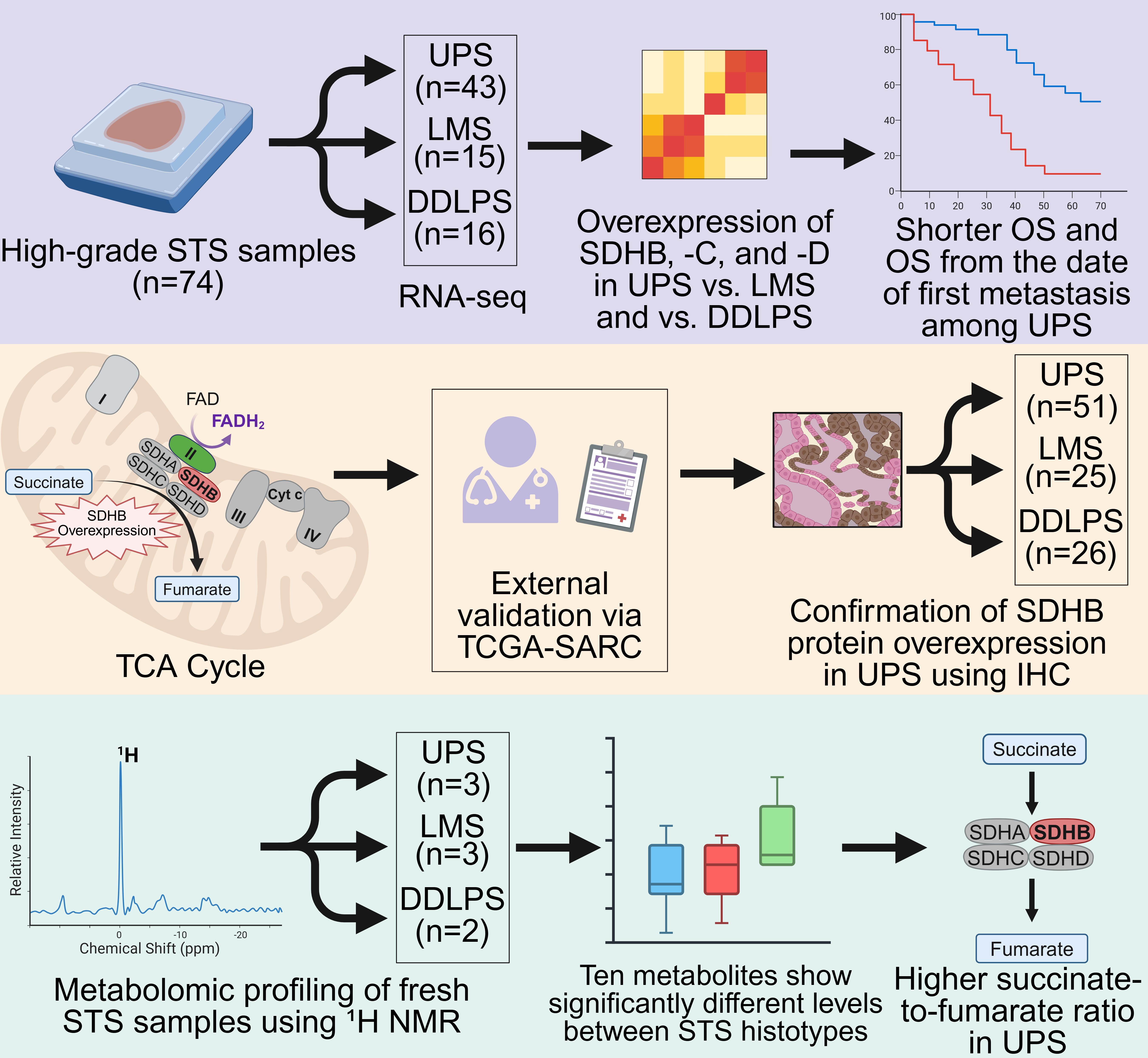

Supplement: Graphical Abstract [file crc-25-0468_graphical_abstract_suppga1.png]

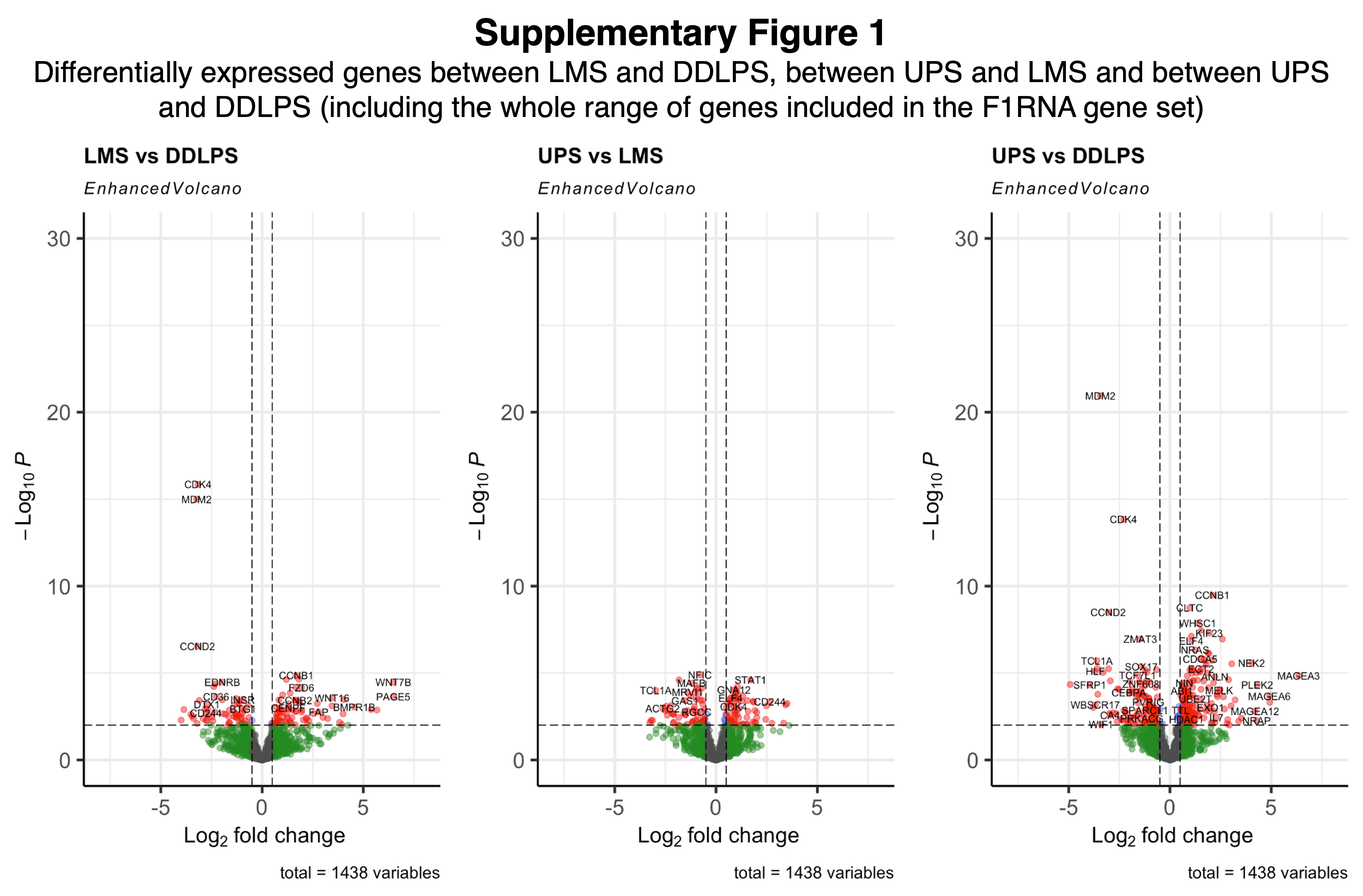

Supplement: Supplementary Figure 1 [file crc-25-0468_supplementary_figure_1_suppsf1.png]

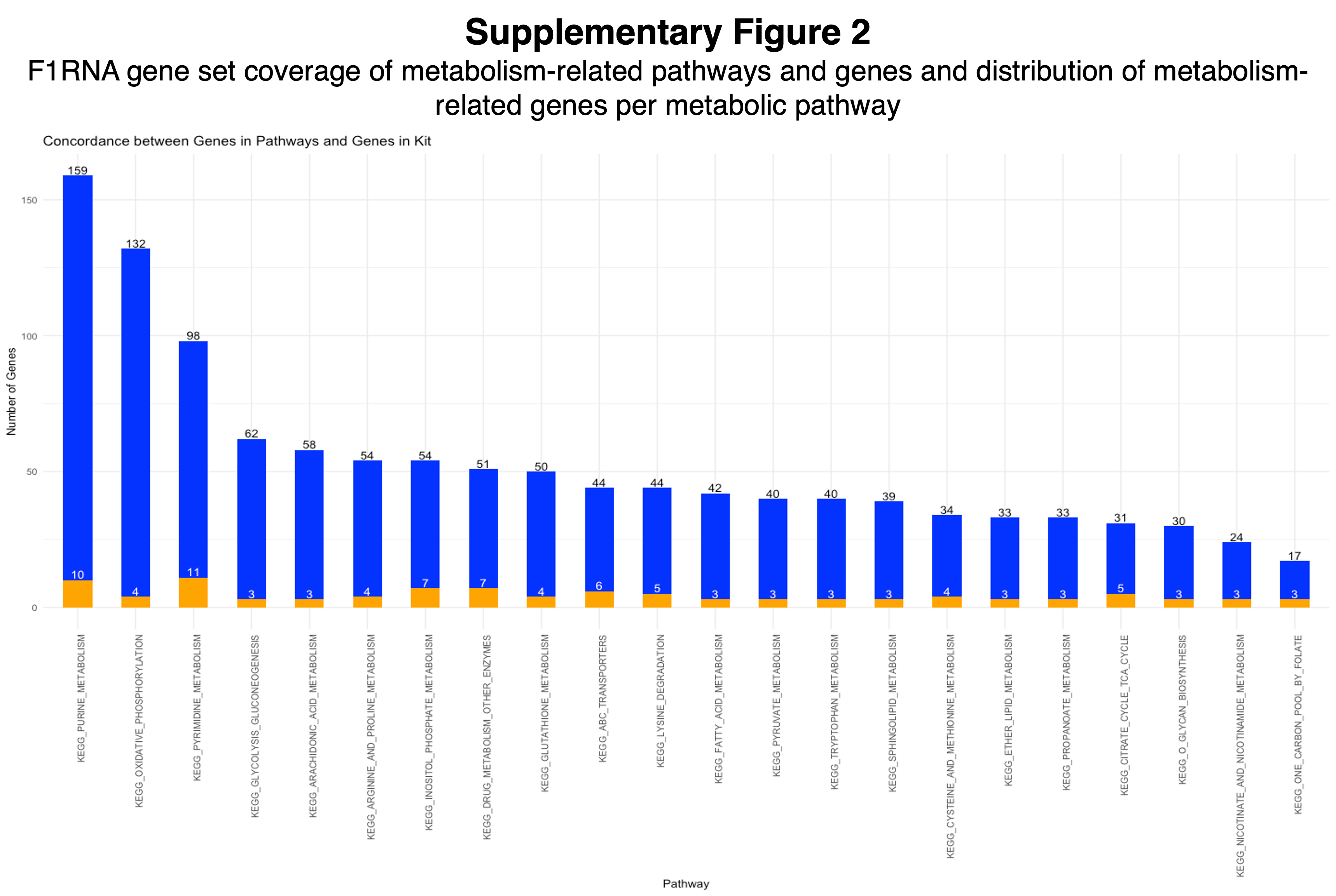

Supplement: Supplementary Figure 2 [file crc-25-0468_supplementary_figure_2_suppsf2.png]

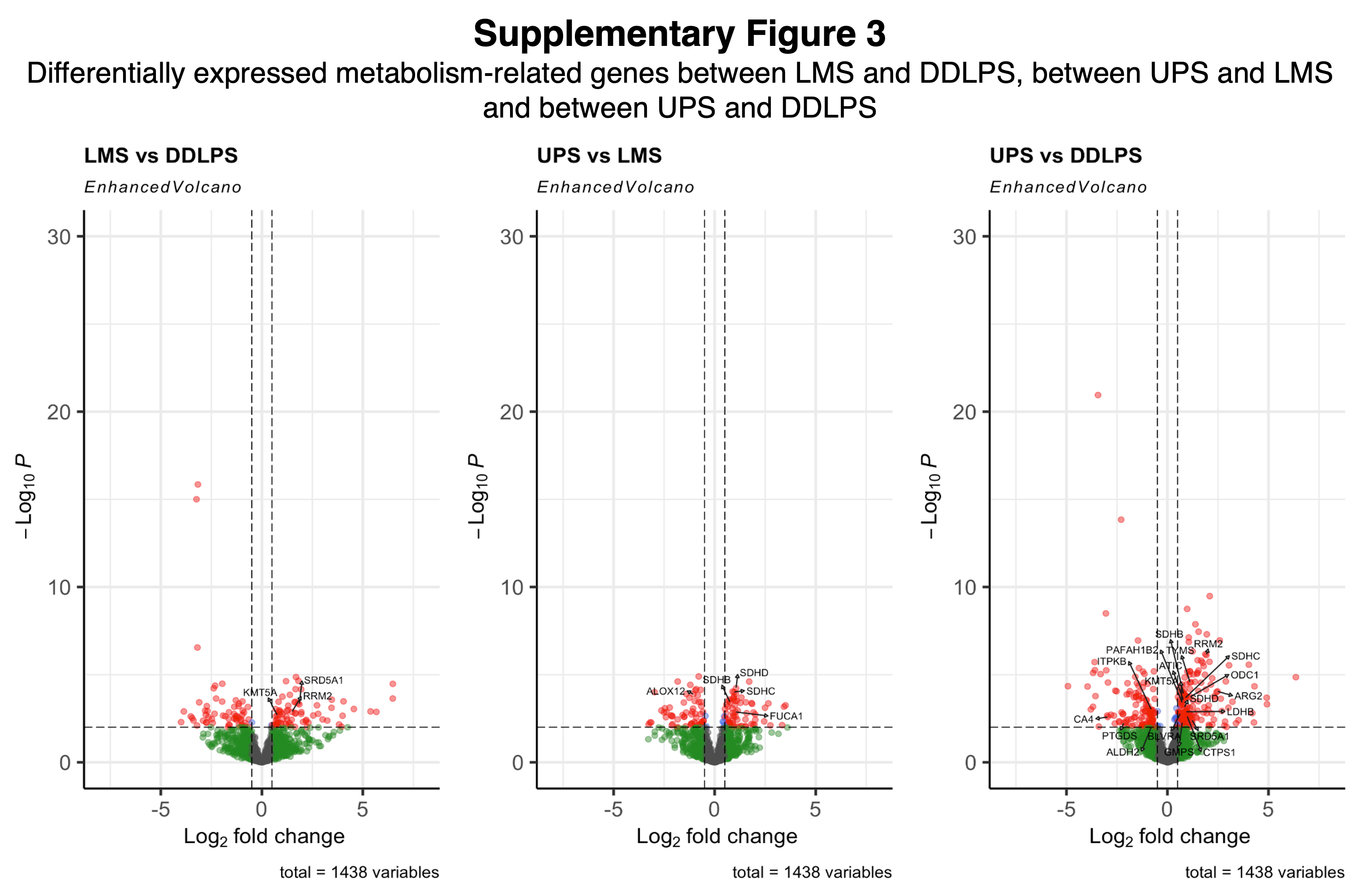

Supplement: Supplementary Figure 3 [file crc-25-0468_supplementary_figure_3_suppsf3.png]

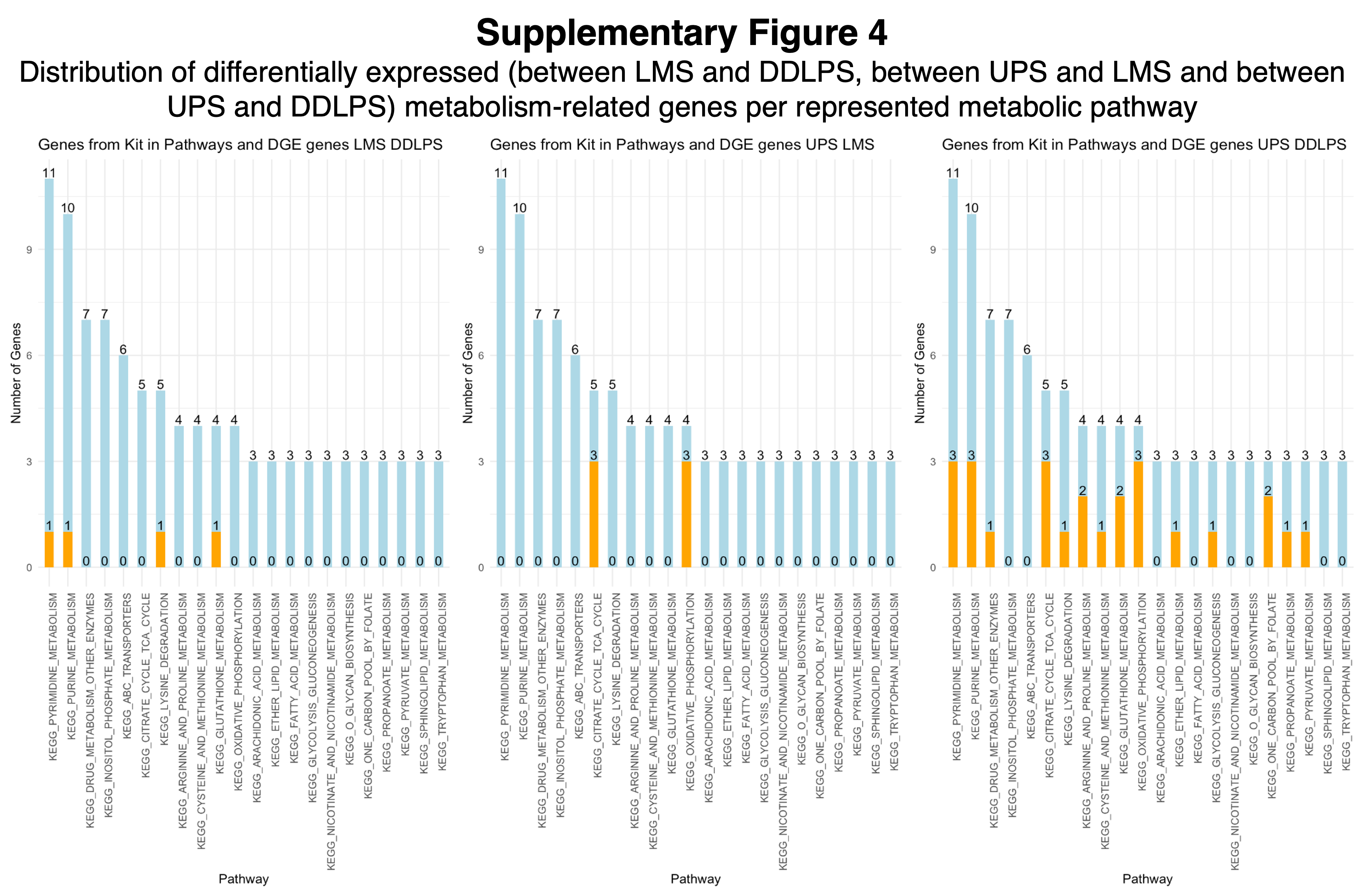

Supplement: Supplementary Figure 4 [file crc-25-0468_supplementary_figure_4_suppsf4.png]

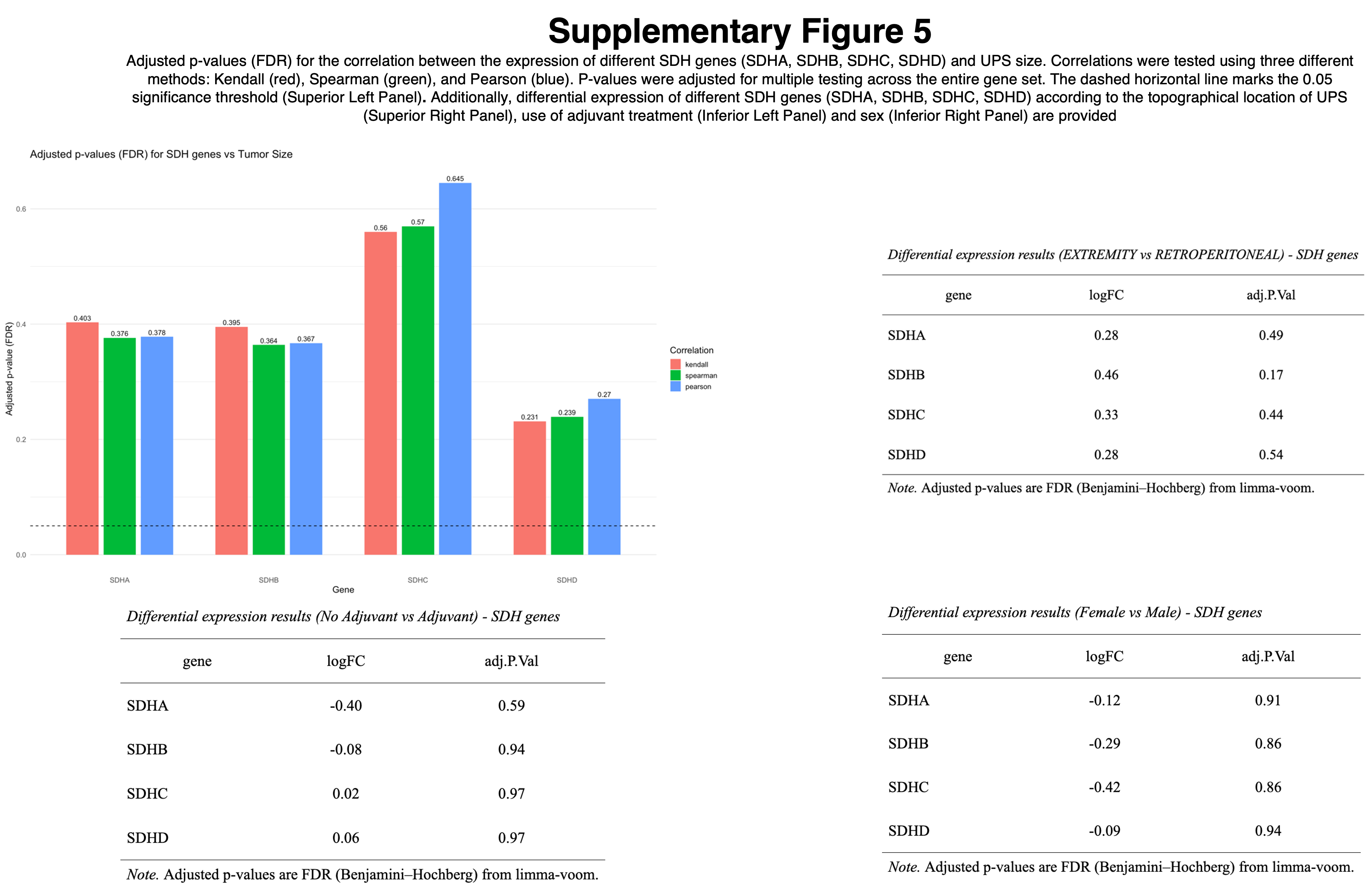

Supplement: Supplementary Figure 5 [file crc-25-0468_supplementary_figure_5_suppsf5.png]

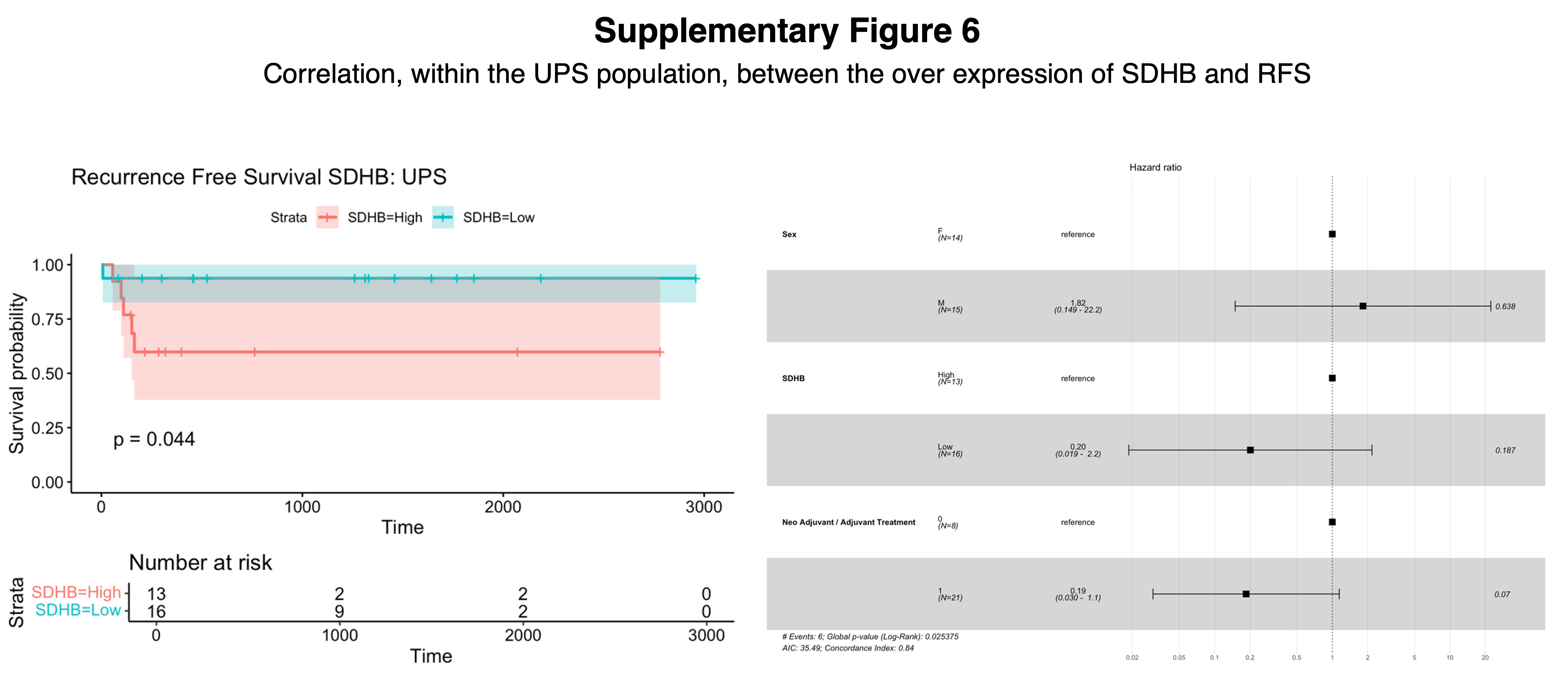

Supplement: Supplementary Figure 6 [file crc-25-0468_supplementary_figure_6_suppsf6.png]

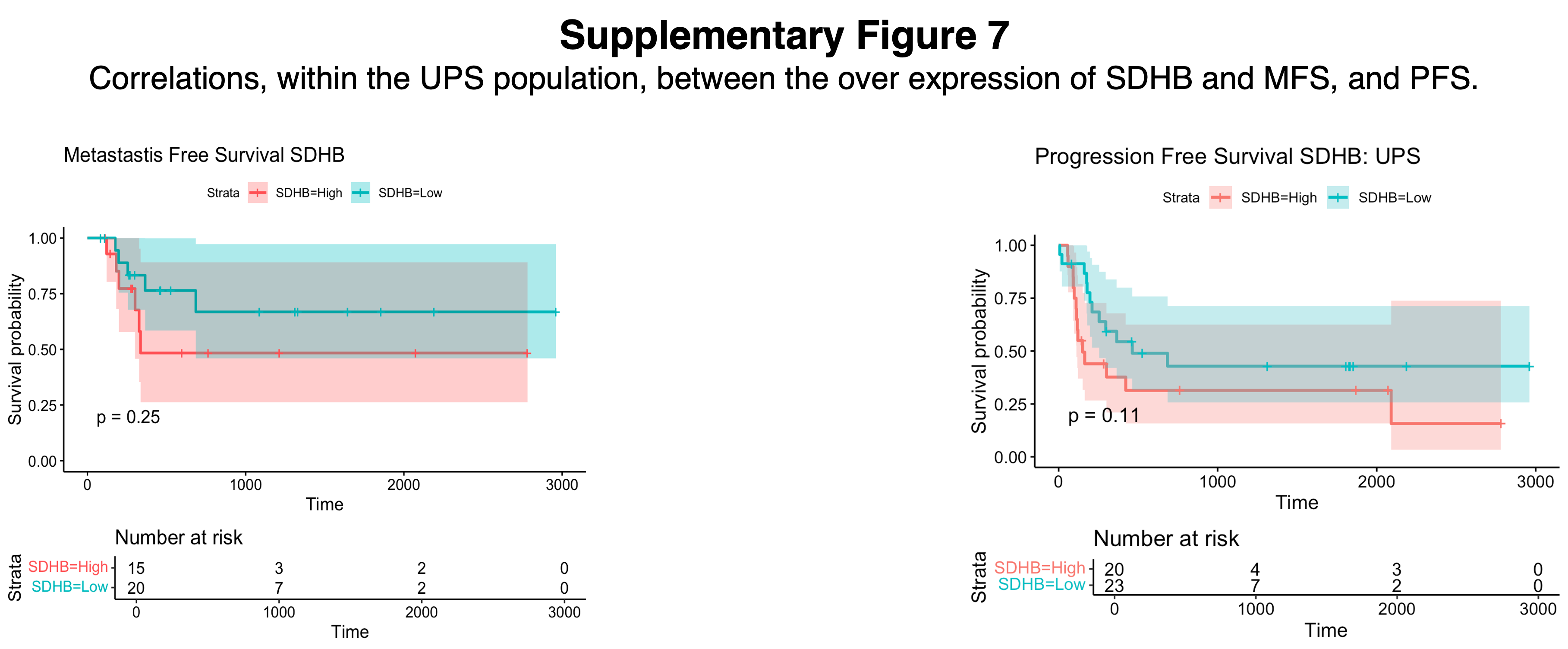

Supplement: Supplementary Figure 7 [file crc-25-0468_supplementary_figure_7_suppsf7.png]

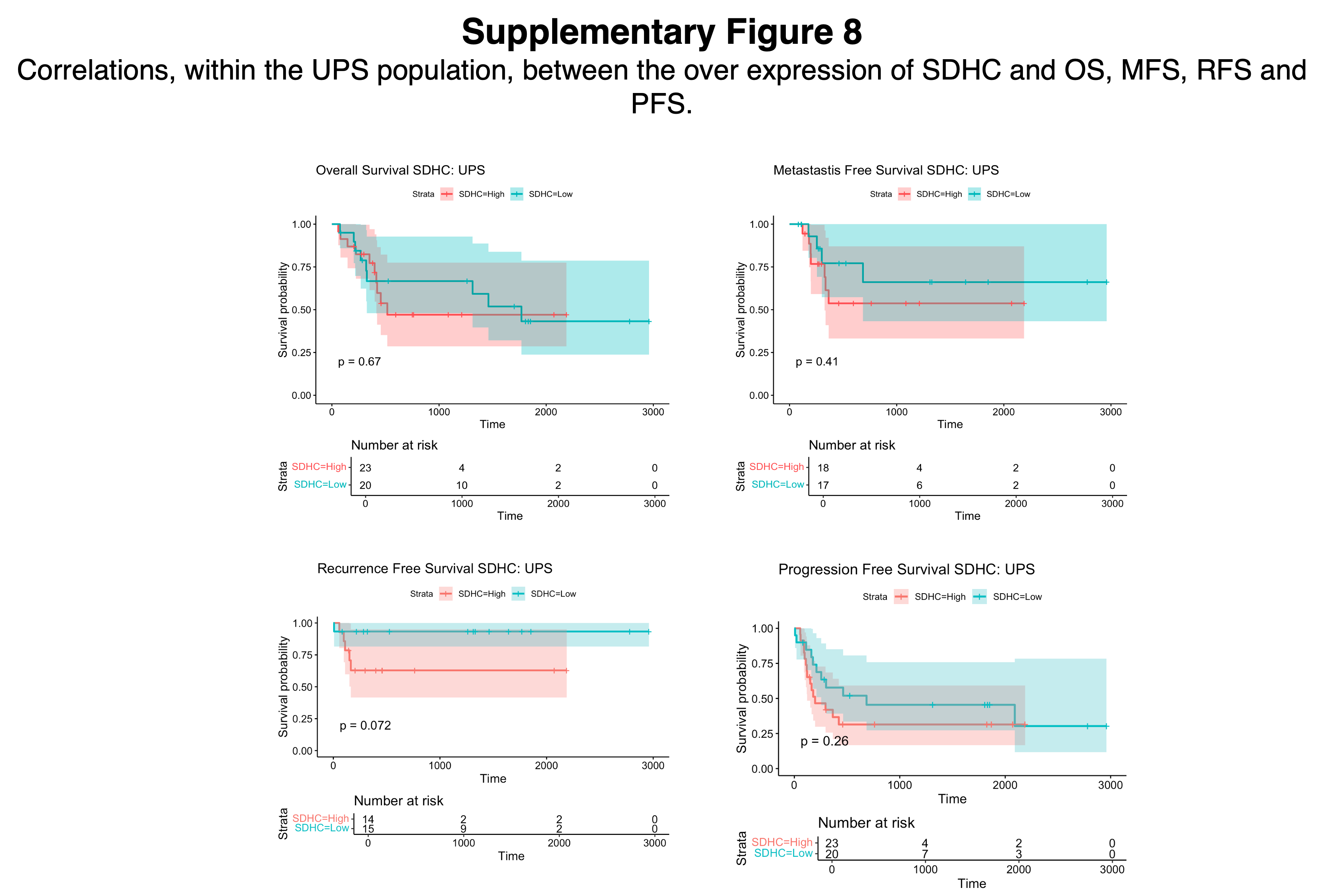

Supplement: Supplementary Figure 8 [file crc-25-0468_supplementary_figure_8_suppsf8.png]

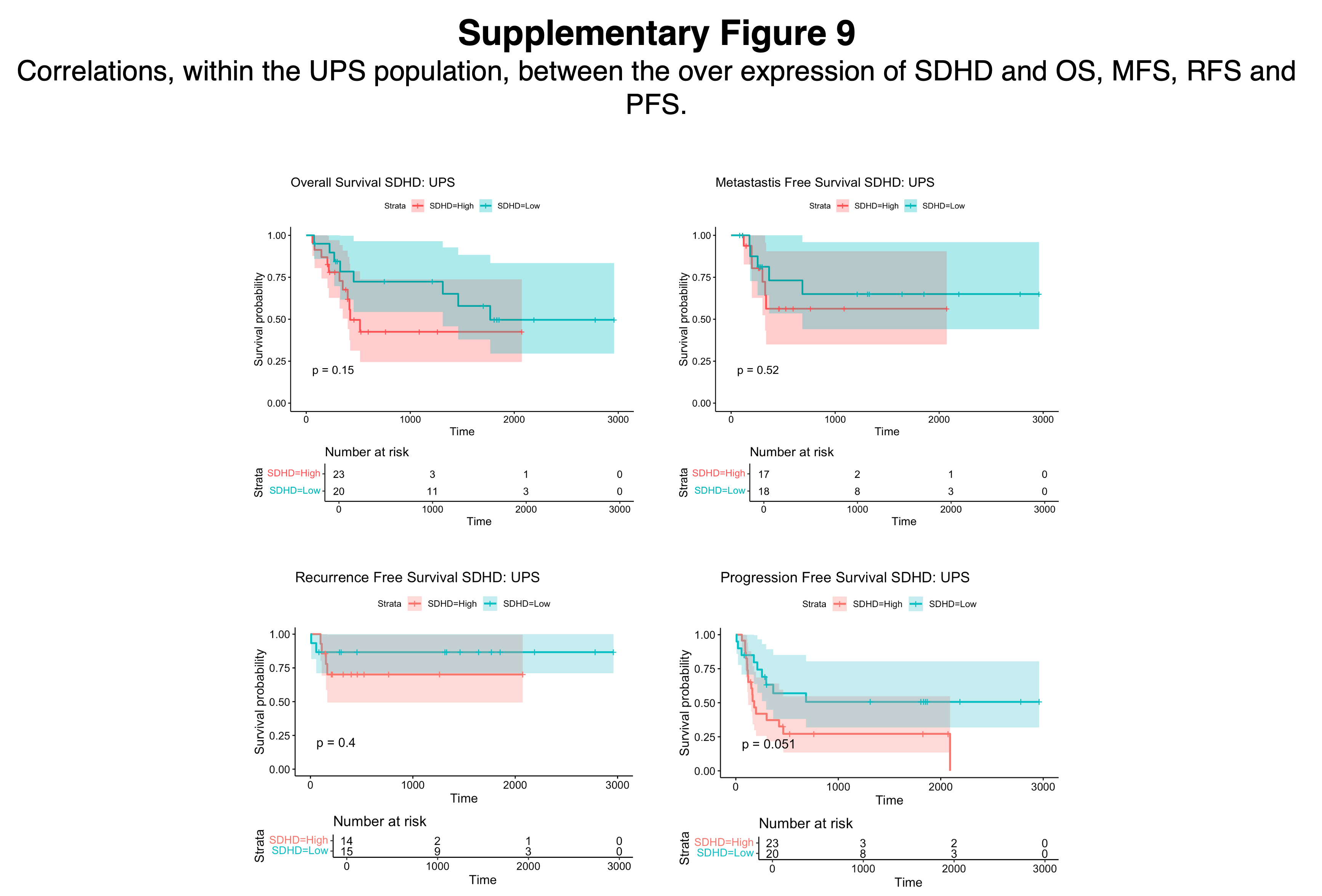

Supplement: Supplementary Figure 9 [file crc-25-0468_supplementary_figure_9_suppsf9.png]

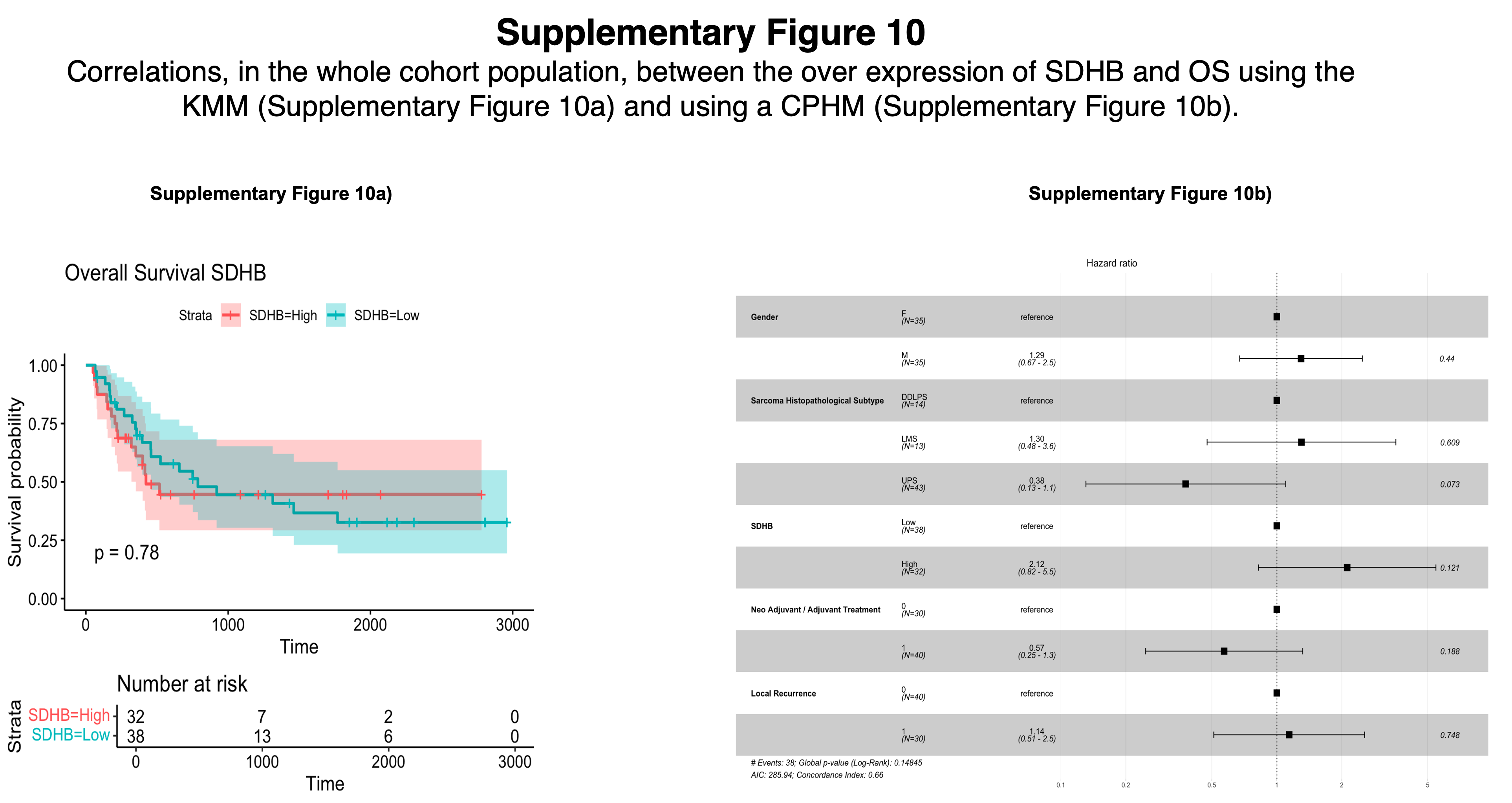

Supplement: Supplementary Figure 10 [file crc-25-0468_supplementary_figure_10_suppsf10.png]

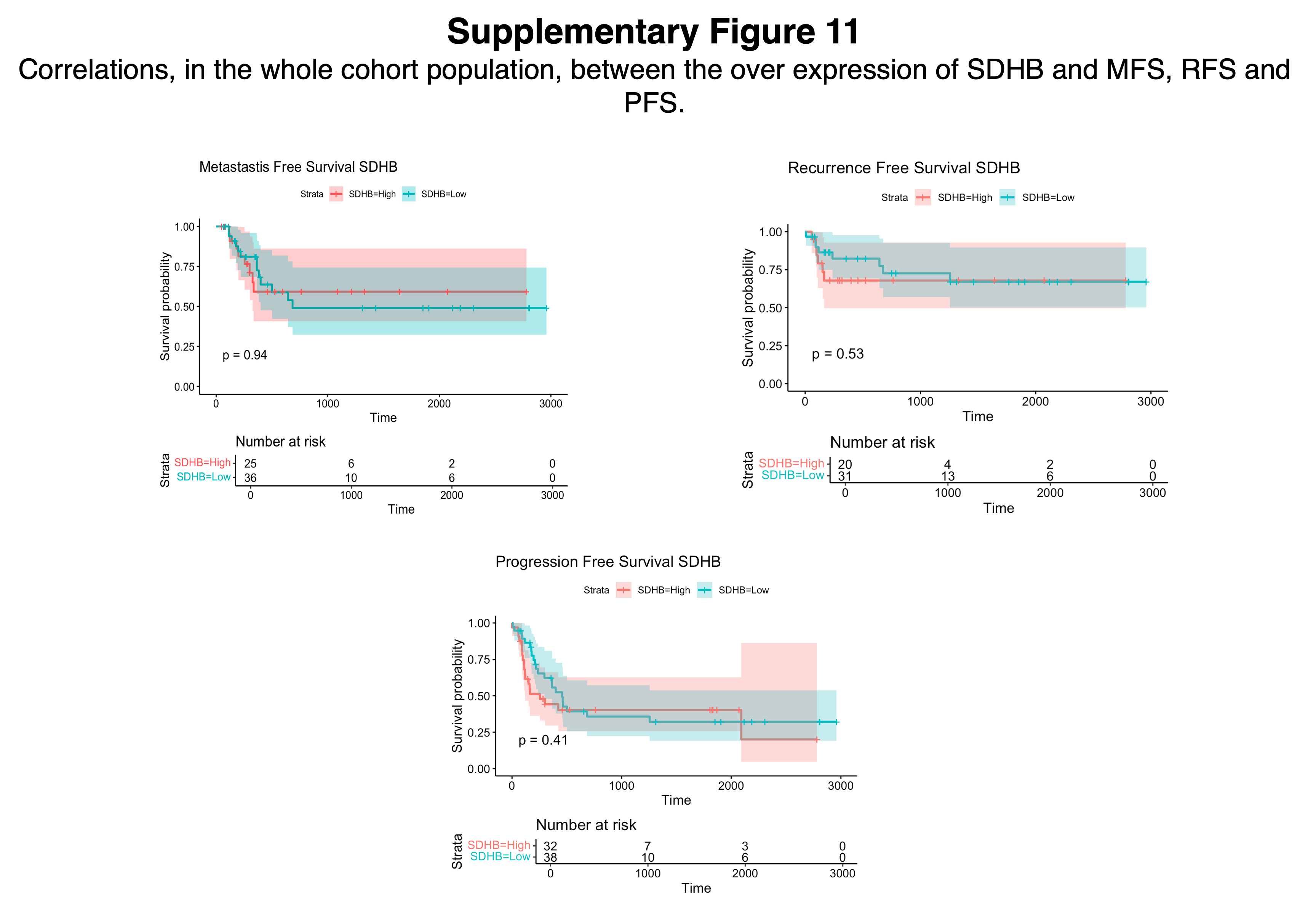

Supplement: Supplementary Figure 11 [file crc-25-0468_supplementary_figure_11_suppsf11.png]

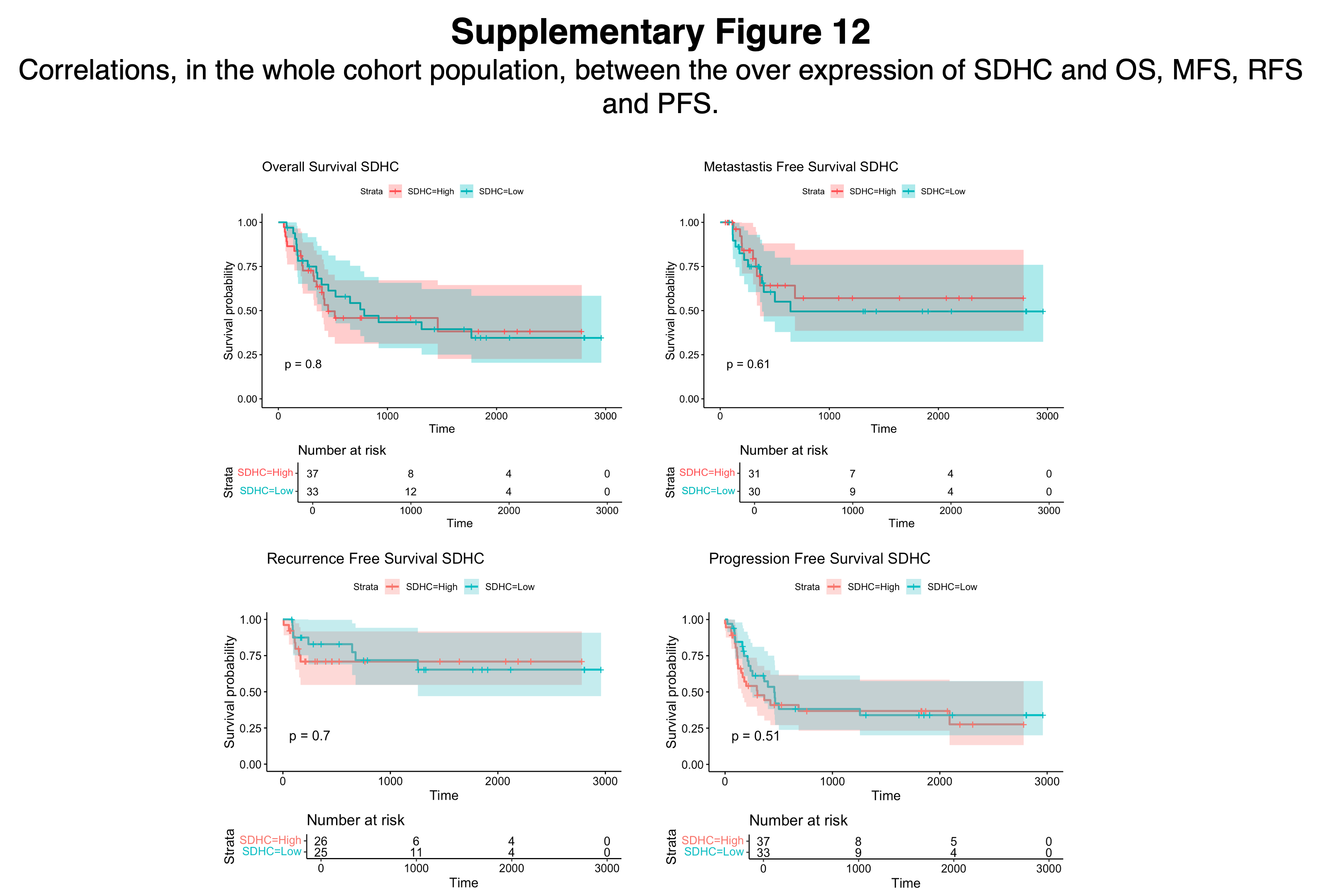

Supplement: Supplementary Figure 12 [file crc-25-0468_supplementary_figure_12_suppsf12.png]

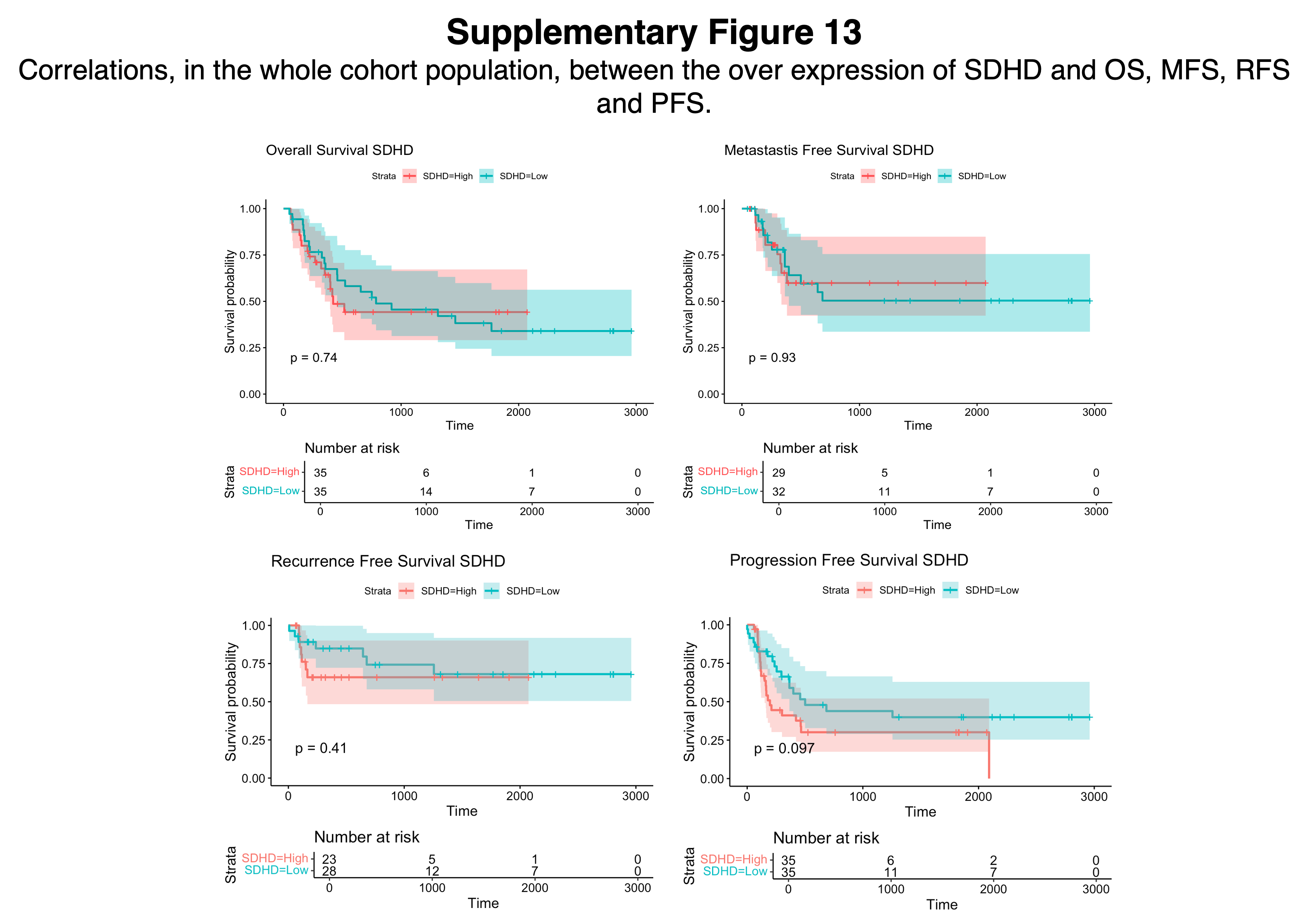

Supplement: Supplementary Figure 13 [file crc-25-0468_supplementary_figure_13_suppsf13.png]

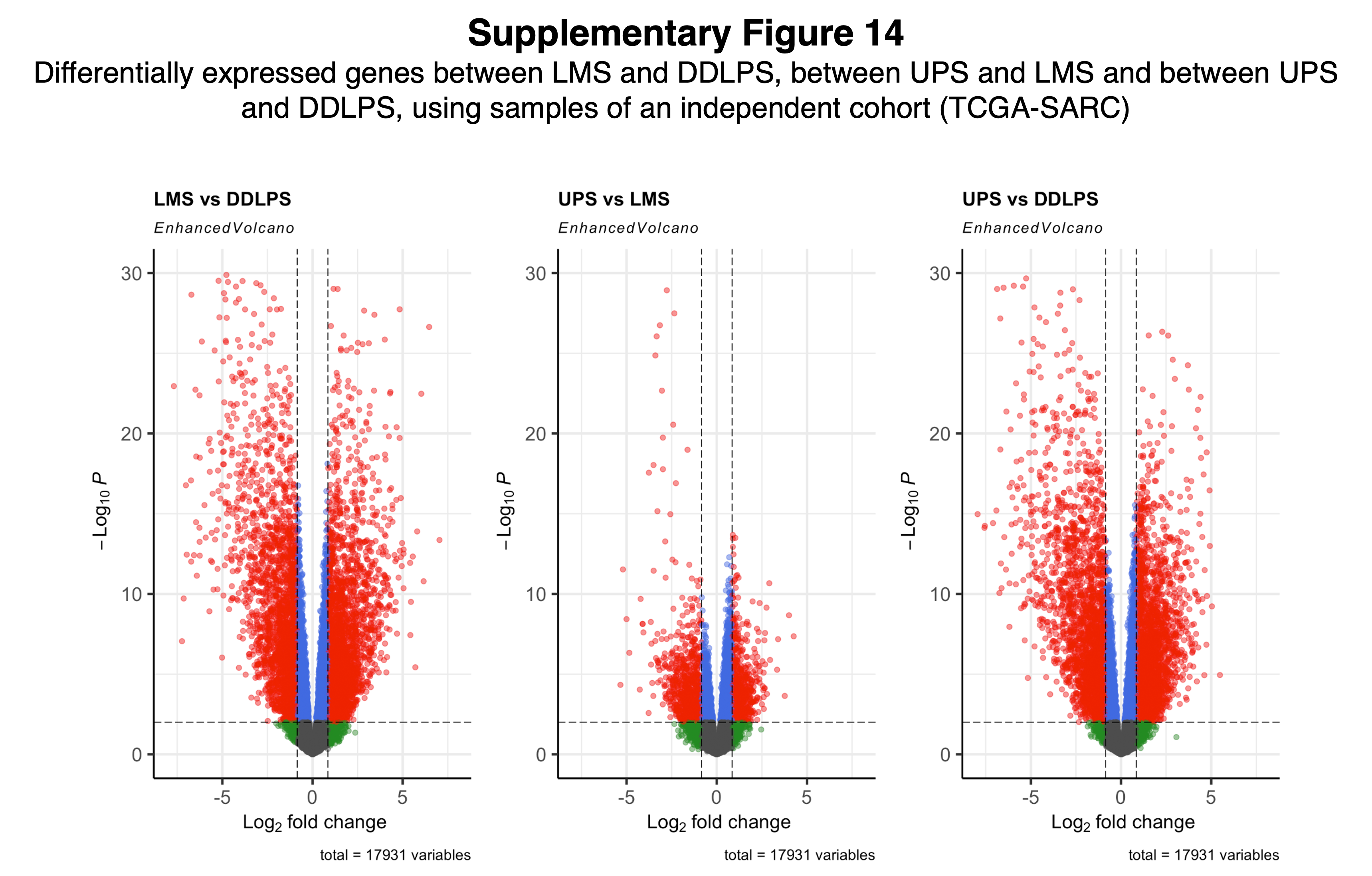

Supplement: Supplementary Figure 14 [file crc-25-0468_supplementary_figure_14_suppsf14.png]

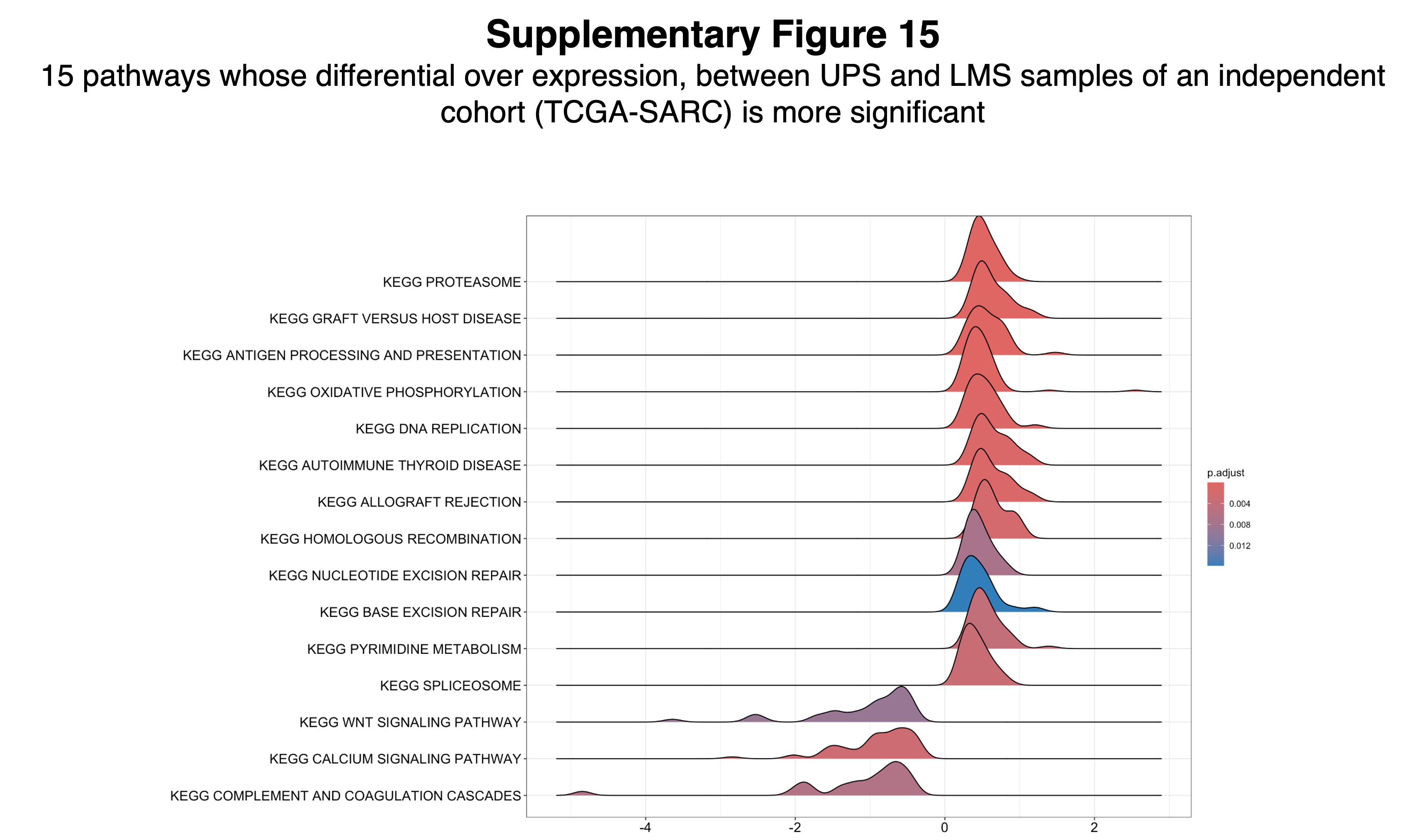

Supplement: Supplementary Figure 15 [file crc-25-0468_supplementary_figure_15_suppsf15.png]

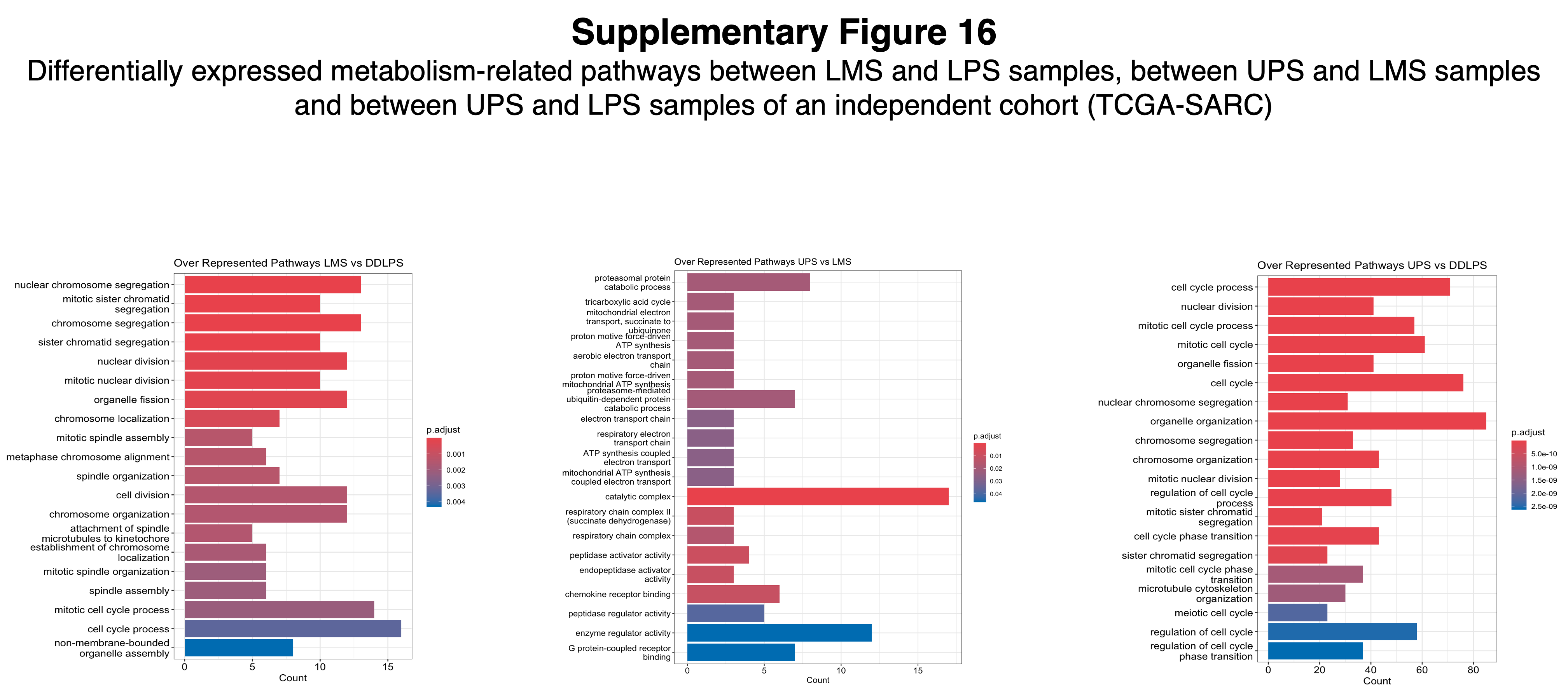

Supplement: Supplementary Figure 16 [file crc-25-0468_supplementary_figure_16_suppsf16.png]

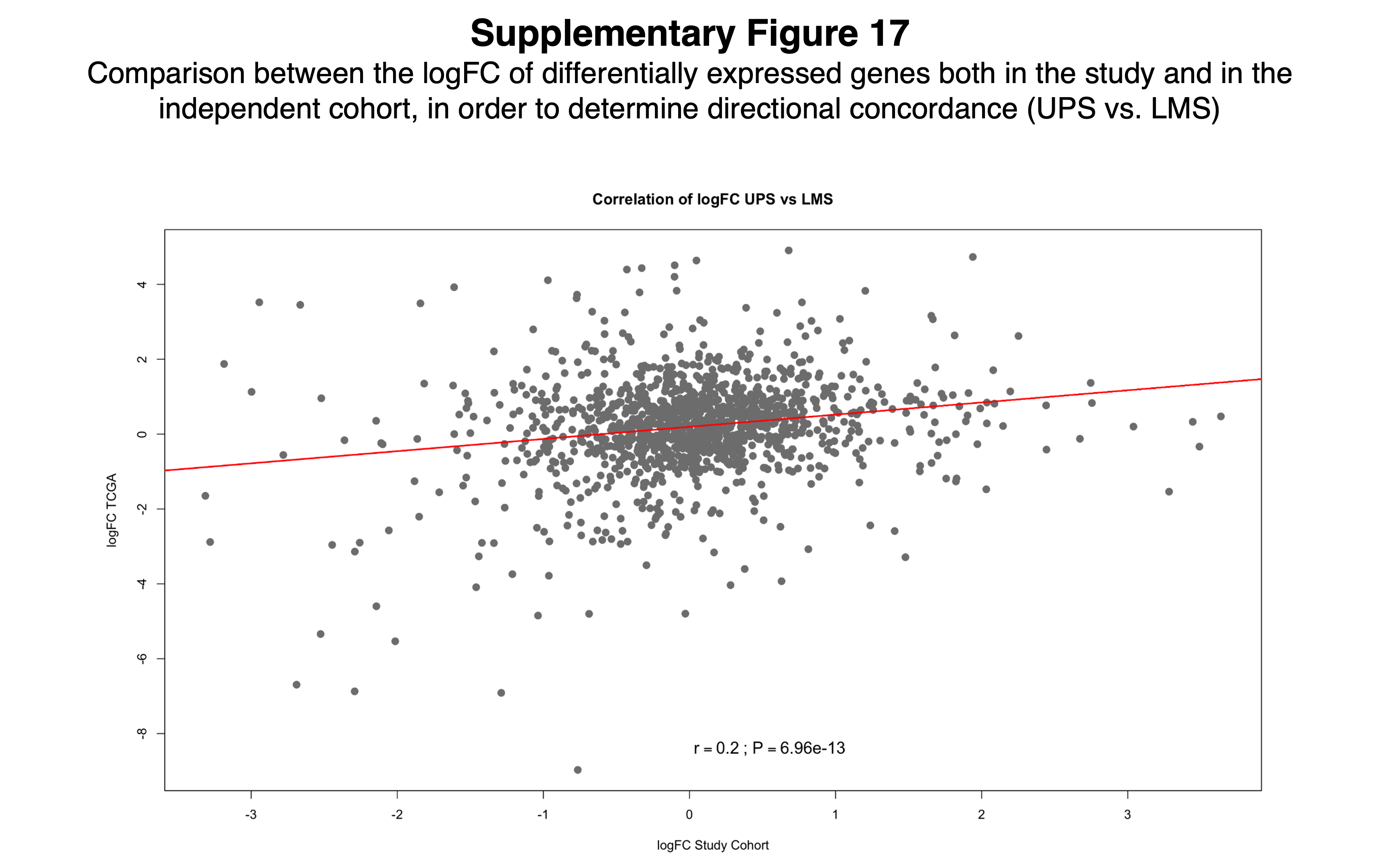

Supplement: Supplementary Figure 17 [file crc-25-0468_supplementary_figure_17_suppsf17.png]

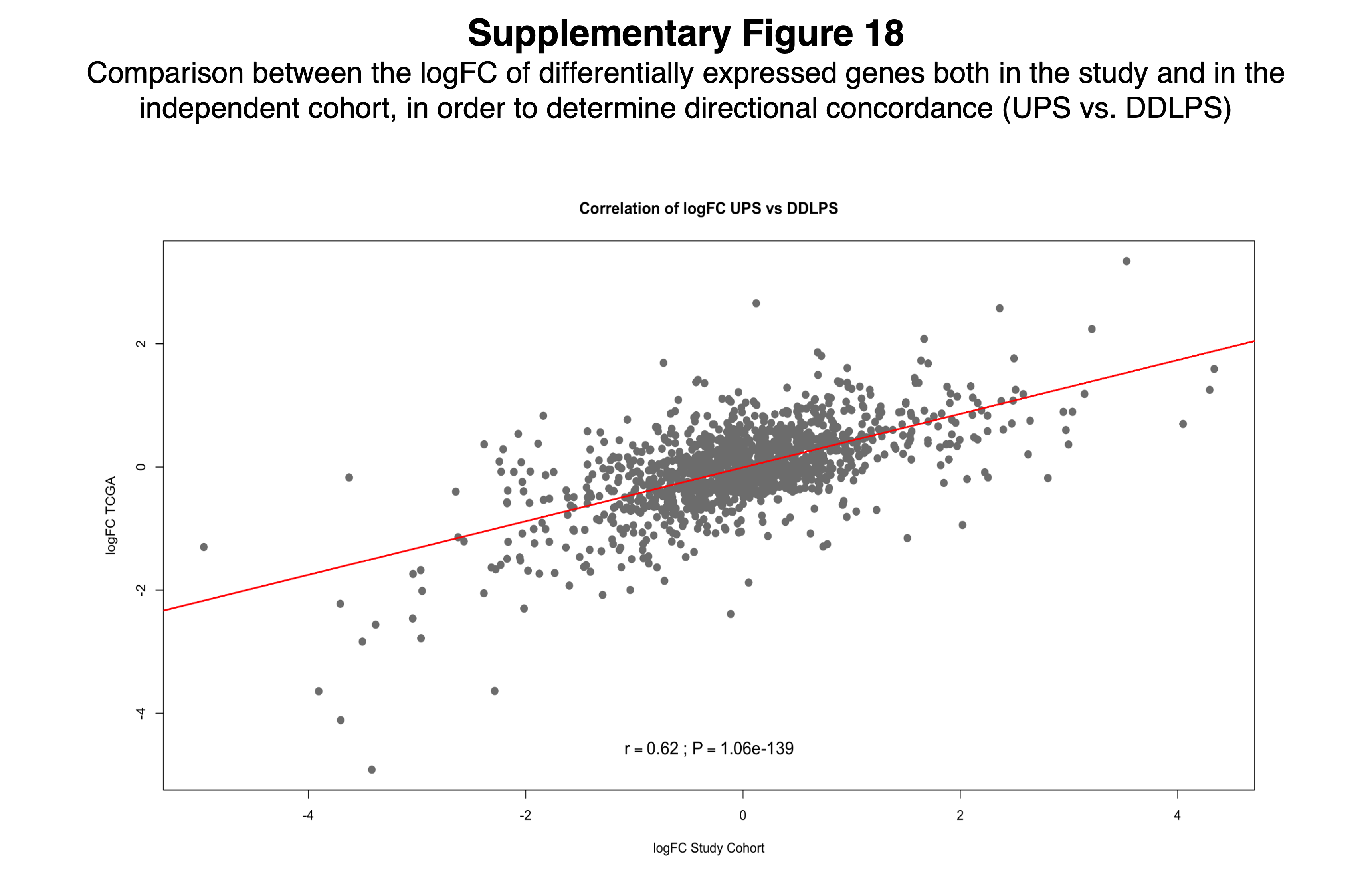

Supplement: Supplementary Figure 18 [file crc-25-0468_supplementary_figure_18_suppsf18.png]

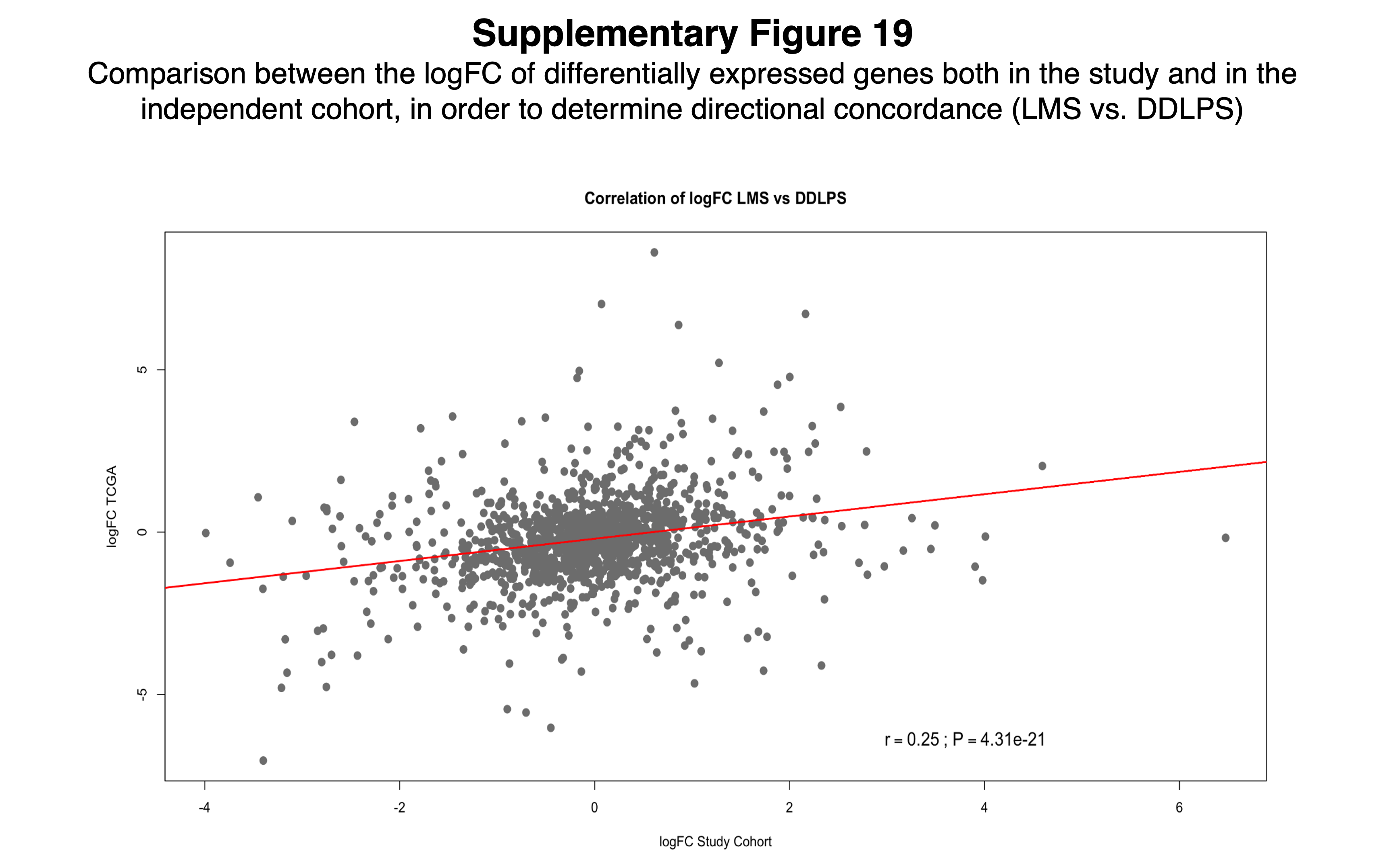

Supplement: Supplementary Figure 19 [file crc-25-0468_supplementary_figure_19_suppsf19.png]

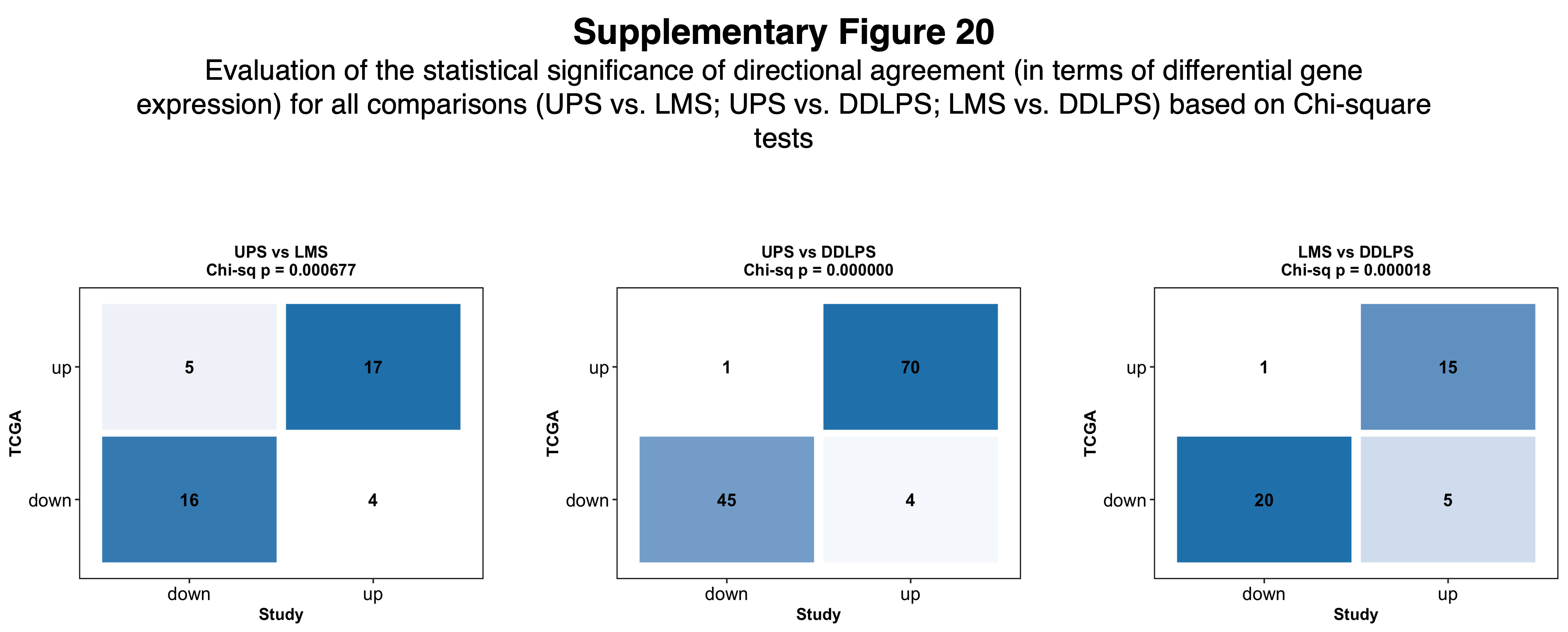

Supplement: Supplementary Figure 20 [file crc-25-0468_supplementary_figure_20_suppsf20.png]

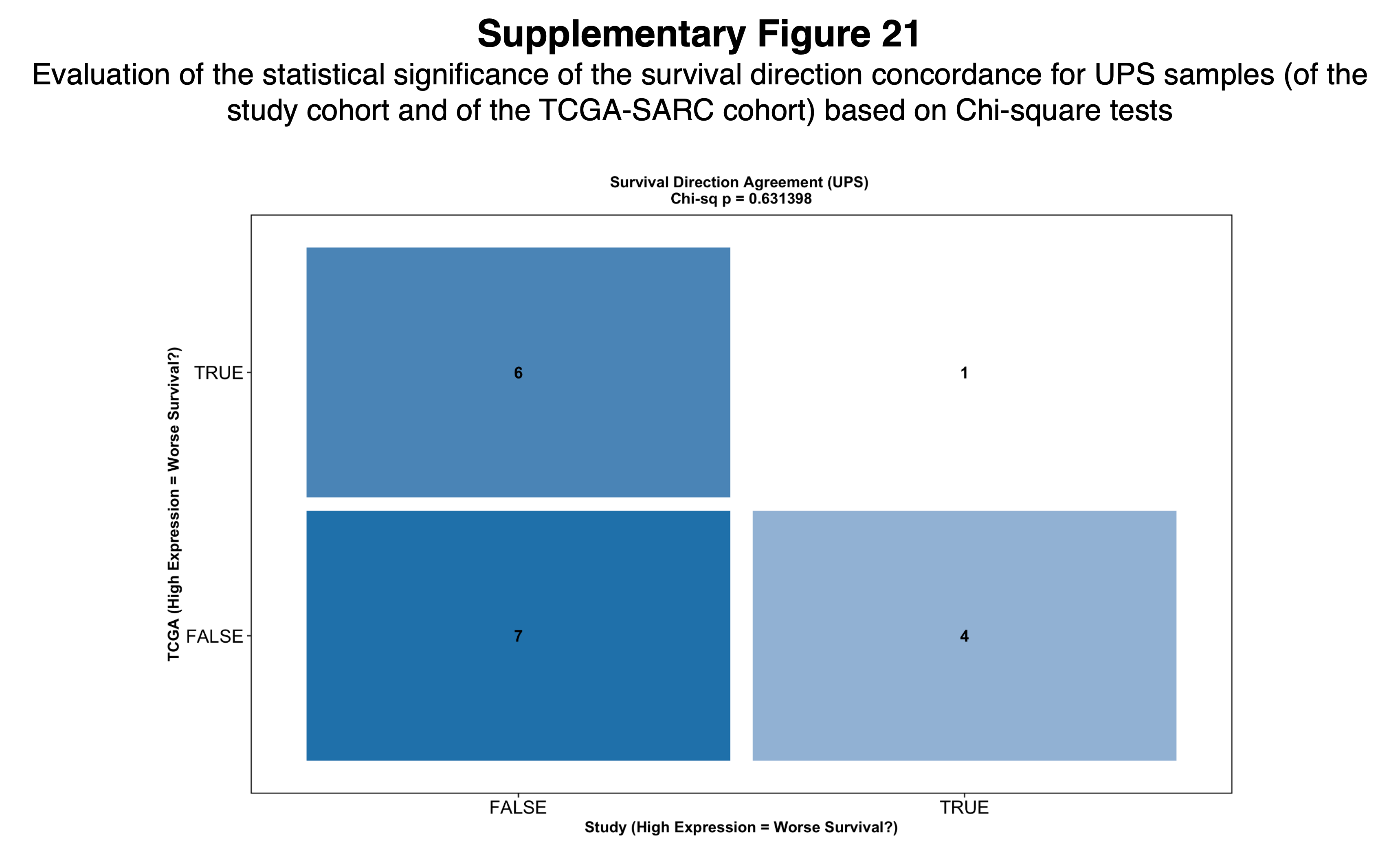

Supplement: Supplementary Figure 21 [file crc-25-0468_supplementary_figure_21_suppsf21.png]

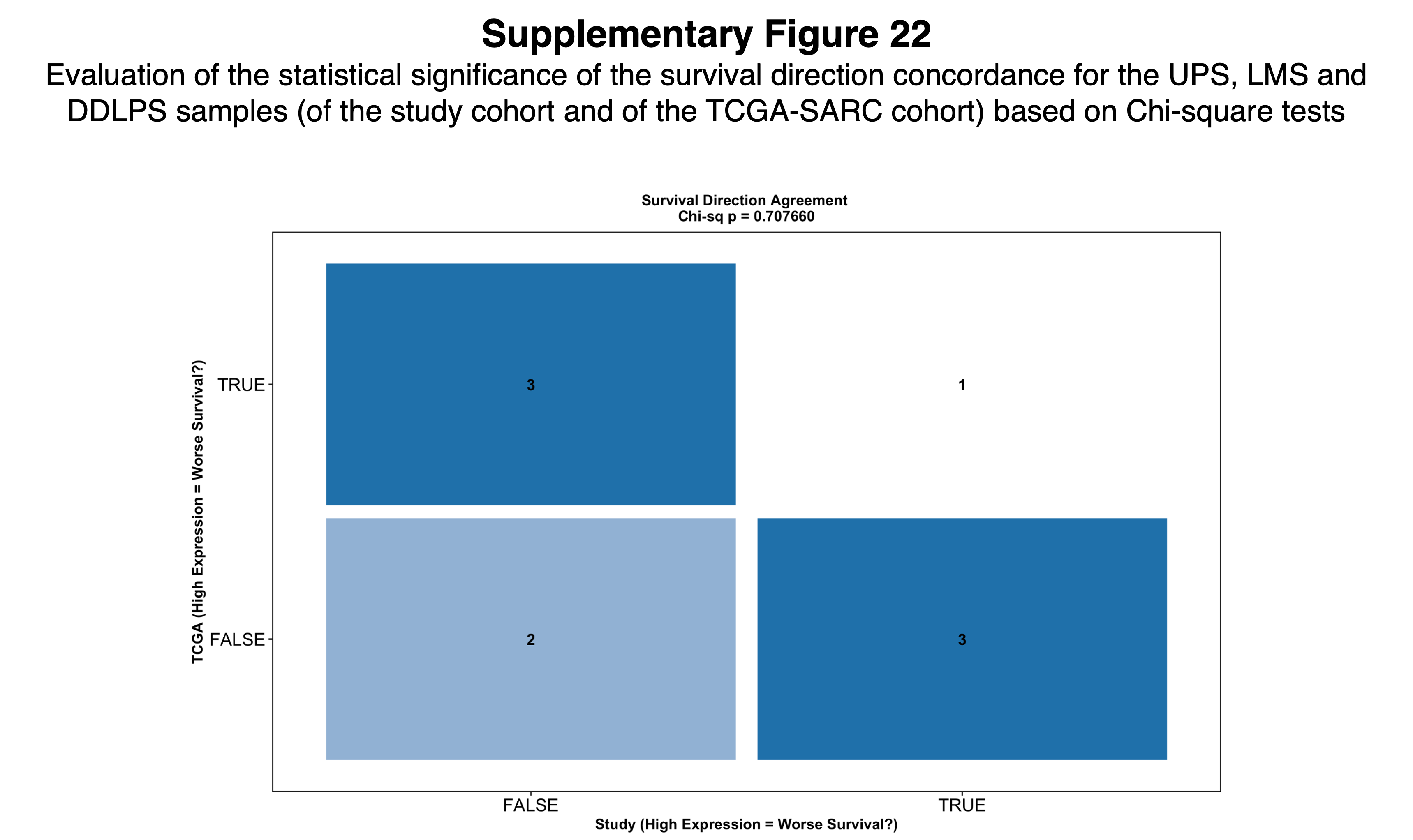

Supplement: Supplementary Figure 22 [file crc-25-0468_supplementary_figure_22_suppsf22.png]

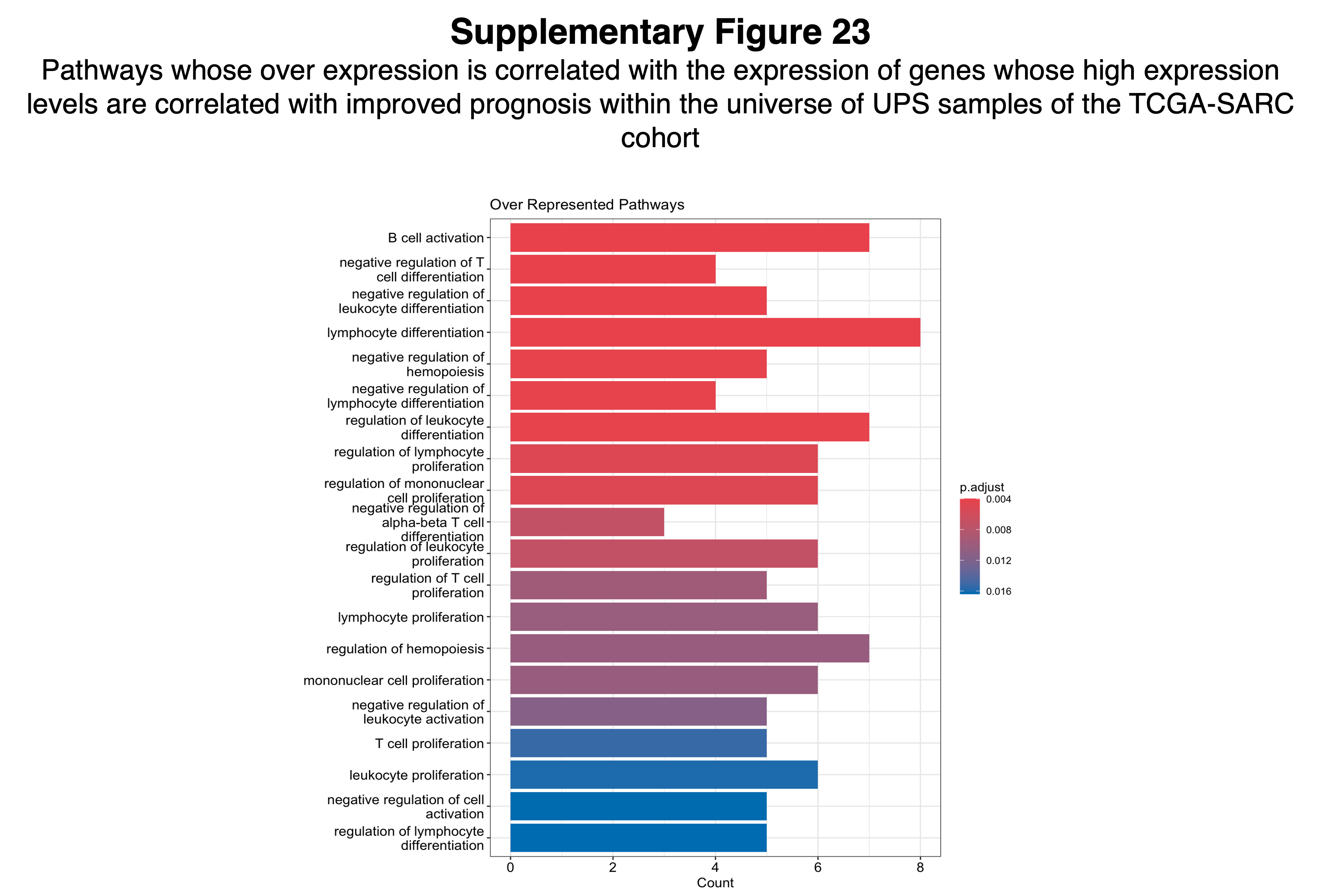

Supplement: Supplementary Figure 23 [file crc-25-0468_supplementary_figure_23_suppsf23.png]

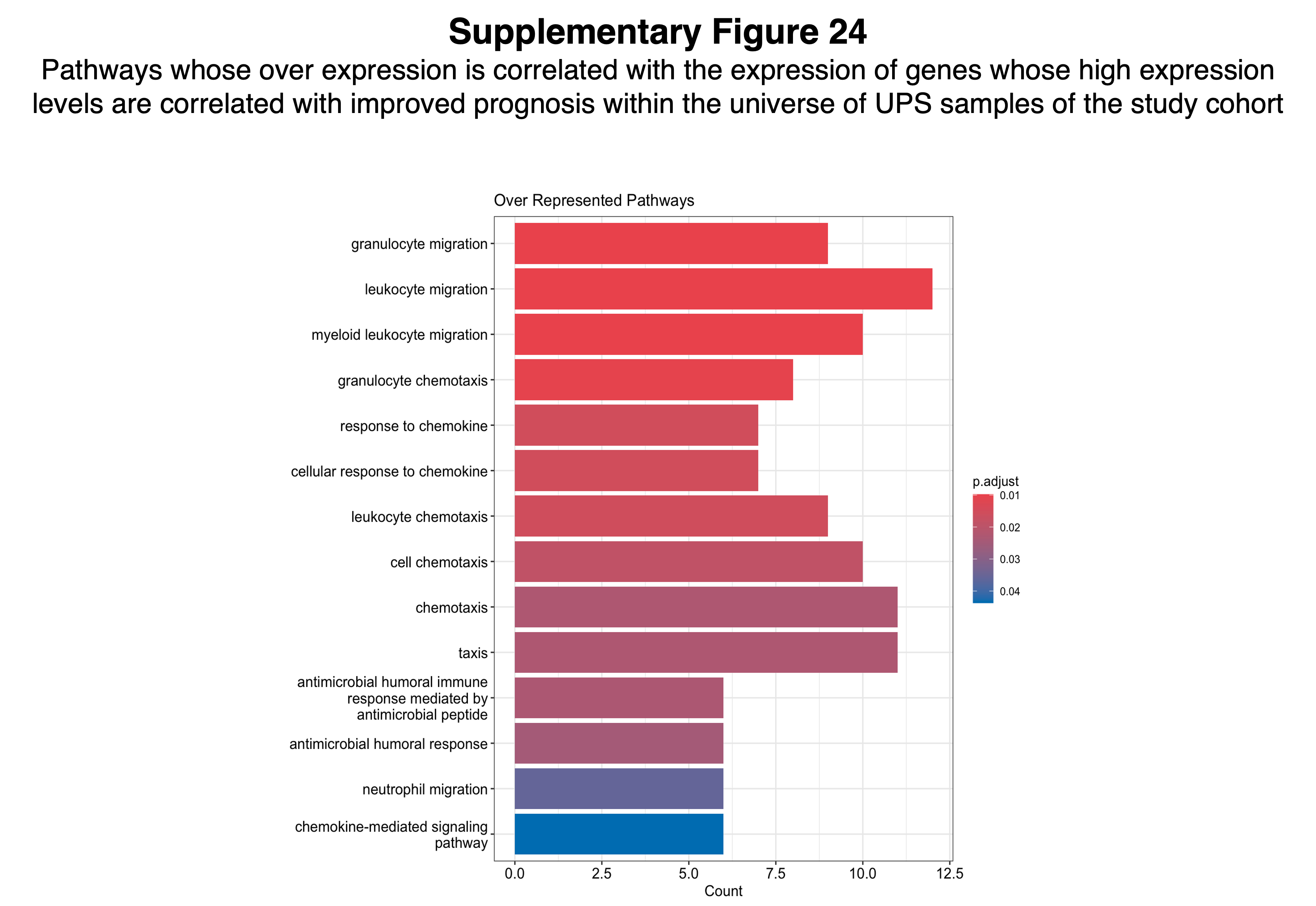

Supplement: Supplementary Figure 24 [file crc-25-0468_supplementary_figure_24_suppsf24.png]

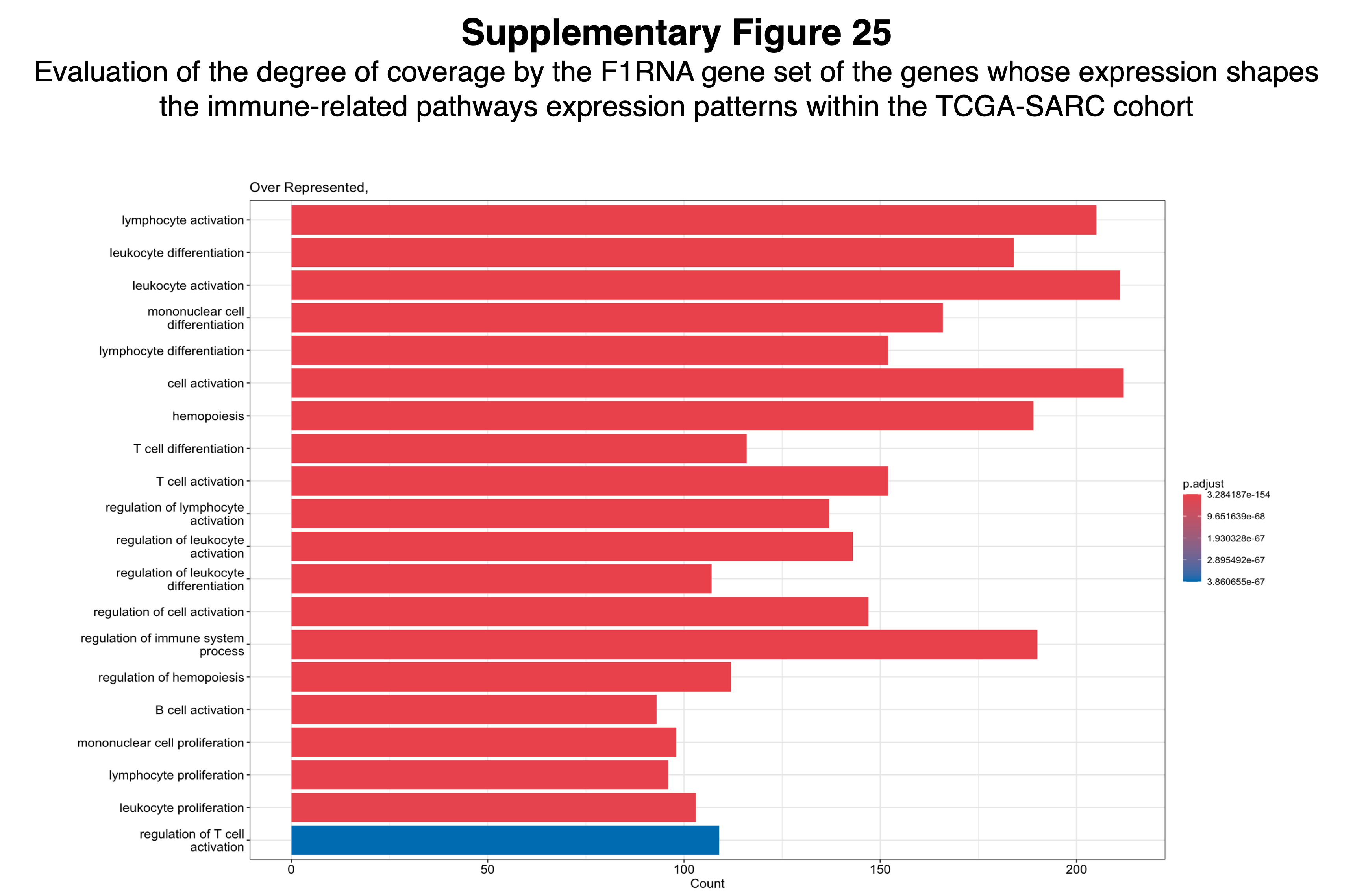

Supplement: Supplementary Figure 25 [file crc-25-0468_supplementary_figure_25_suppsf25.png]

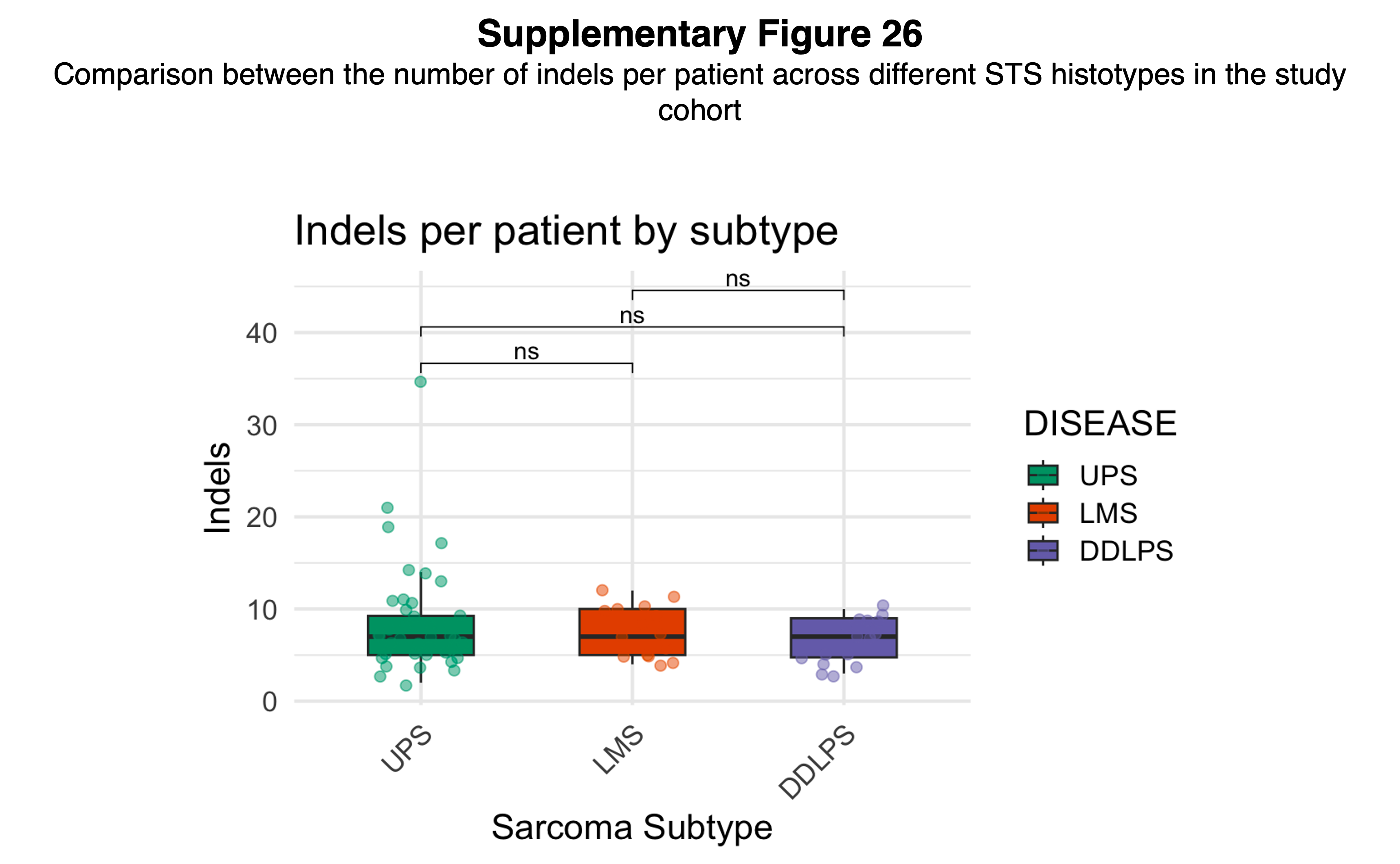

Supplement: Supplementary Figure 26 [file crc-25-0468_supplementary_figure_26_suppsf26.png]

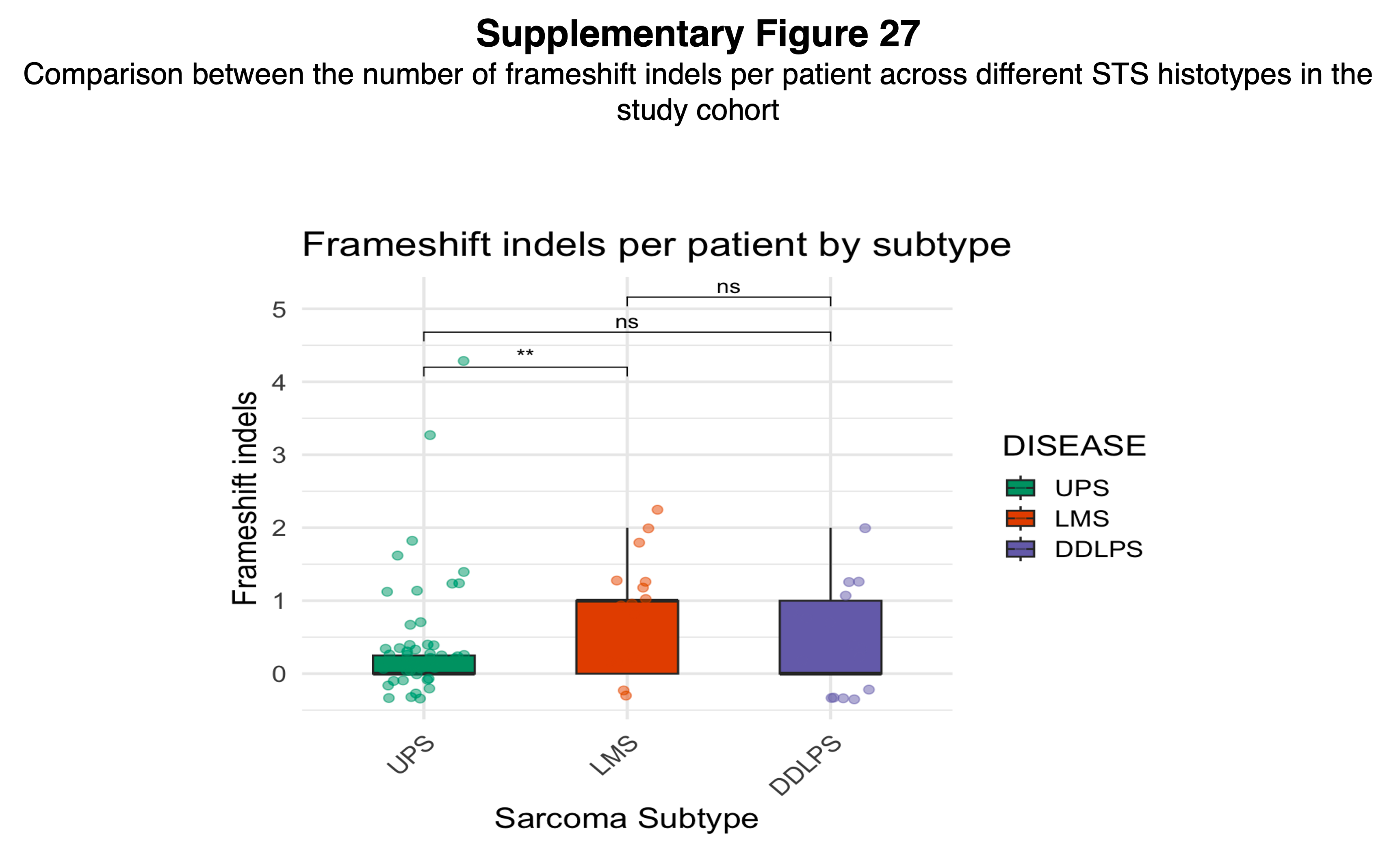

Supplement: Supplementary Figure 27 [file crc-25-0468_supplementary_figure_27_suppsf27.png]

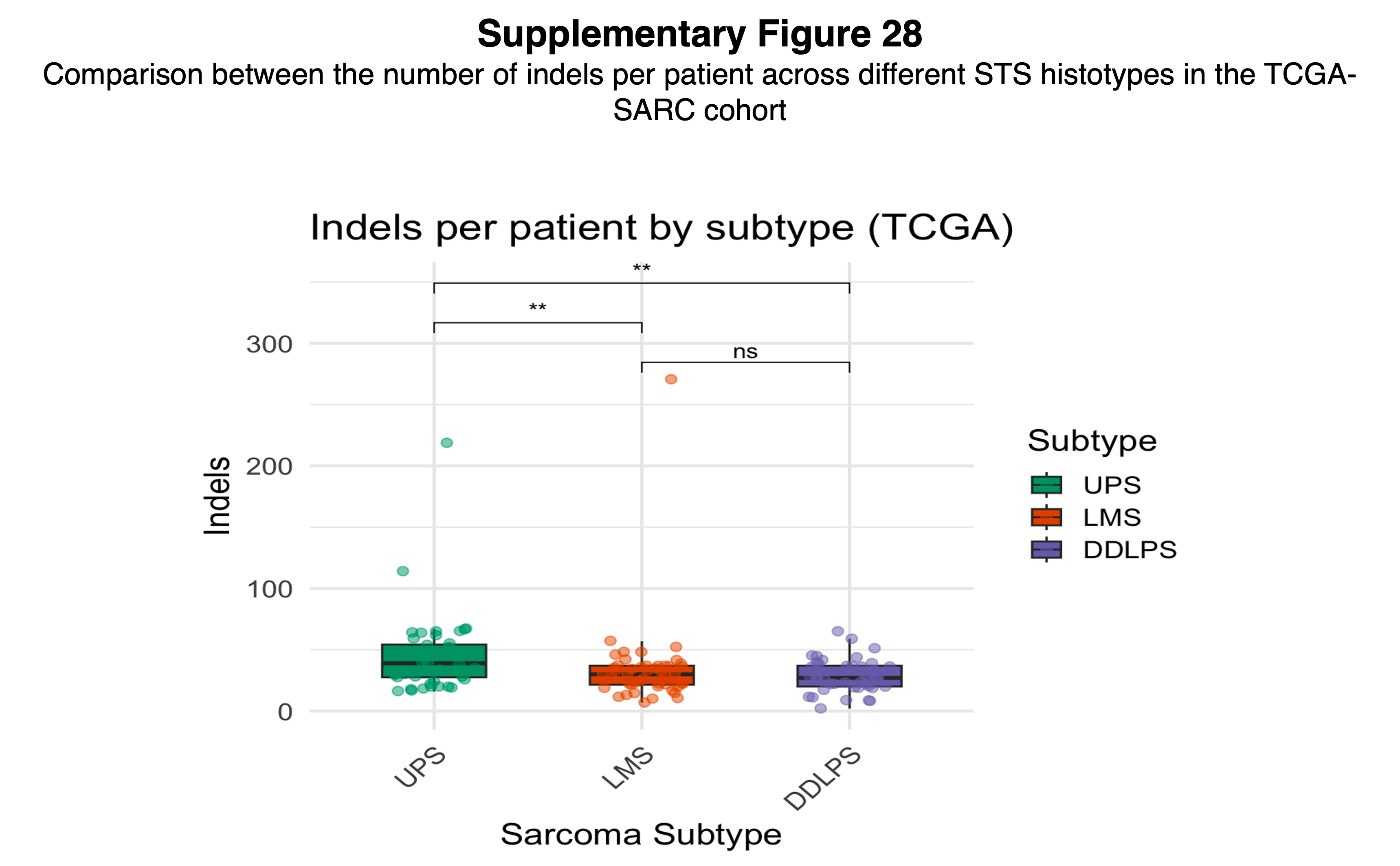

Supplement: Supplementary Figure 28 [file crc-25-0468_supplementary_figure_28_suppsf28.png]

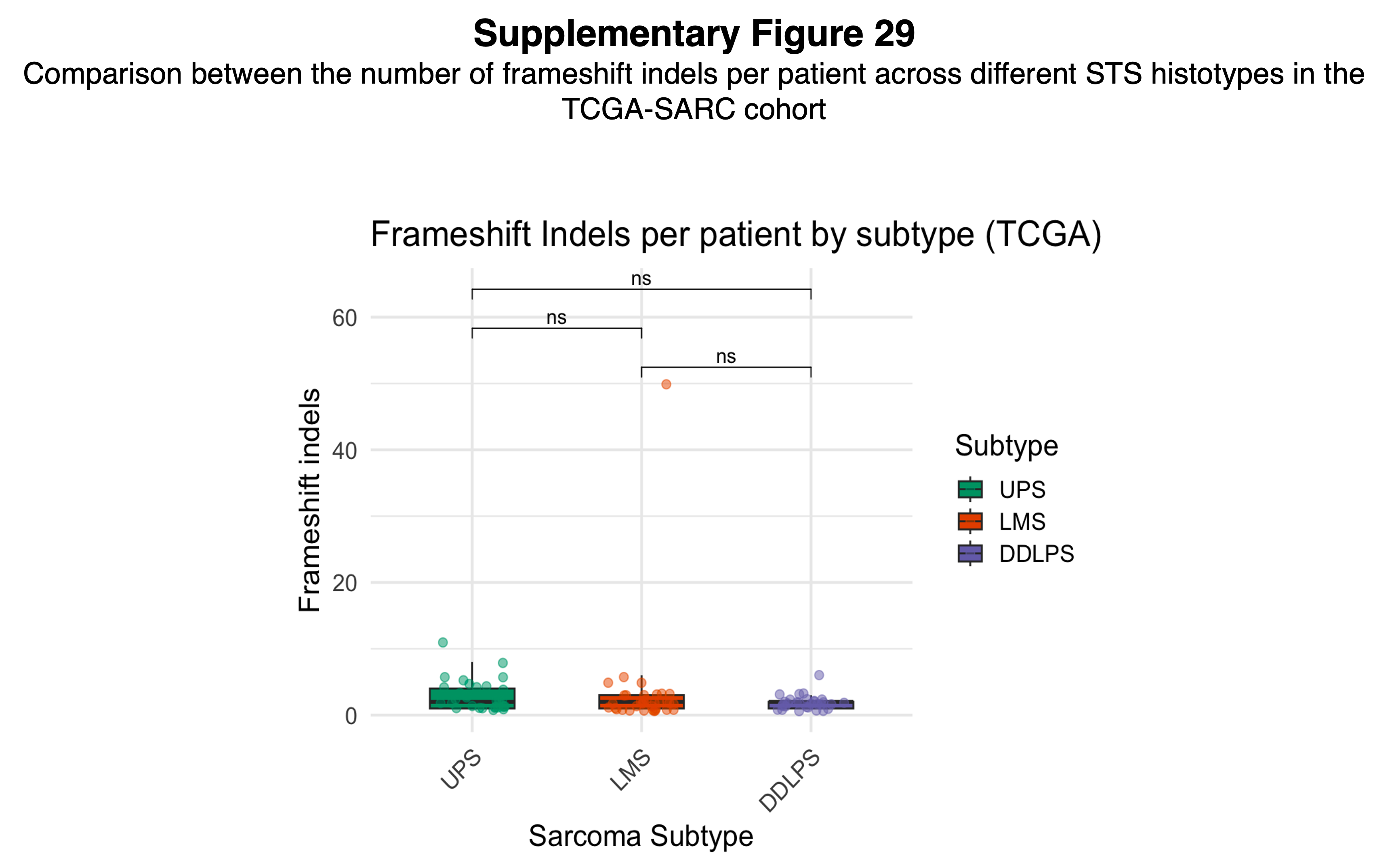

Supplement: Supplementary Figure 29 [file crc-25-0468_supplementary_figure_29_suppsf29.png]
