# Supplementary material for: Succinate Dehydrogenase B (SDHB) Overexpression with Enzymatic Dysfunction Defines a Distinct Subtype of Undifferentiated Pleomorphic Sarcoma
Source: Cancer Res Commun. 2025 Oct 30;5(10):1934–45. doi: 10.1158/2767-9764.CRC-25-0468 (PMC12573234; doi:10.1158/2767-9764.CRC-25-0468)
Supplement: Supplementary Results — Characterization of the cohort of STS patients whose samples were used for the exploratory metabolomic characterization using 1H NMR [file crc-25-0468_supplementary_results_suppsr1.docx]

**Supplementary Results**

Characterization of the cohort of STS patients whose samples were used for the exploratory metabolomic characterization using ^1^H NMR

This group of patients was composed by 16 patients with soft tissue or bone sarcomas. The median age at diagnosis was 74 years (interquartile range [IQR]: 33.4), and the majority were female (n = 11, 68.8%). Primary tumor localization was predominantly in the lower limbs (n = 7, 43.8%), followed by the upper limbs (n = 3, 18.8%) and the retroperitoneum (n = 3, 18.8%).

This set of fresh tissue samples featured a diverse range of histological subtypes, including: 4 UPS, 3 LMS, 2 LPS—1 DDLPS and 1 MLPS -, 1 DFSP, 1 SFT, 1 SS, 1 CS, 1 CCSLGT, 1 low-grade ESS, and 1 ADM.

Regarding tumor grade, the majority of tumors in the fresh tissue samples set were high-grade (G3) lesions (n = 9, 56.3%), followed by intermediate-grade (G2) tumors (n = 5, 31.3%) and low-grade (G1) tumors (n = 2, 12.5%).

Most tissue samples were obtained from primary tumor sites (n = 13, 81.3%), whereas three samples (18.7%) were derived from patients with metastatic disease at the time of sample collection.

All patients underwent surgical treatment. Surgical procedures were performed at both centers in all cases, with curative intent in 13 patients (81.2%) and palliative intent in 3 patients (18.8%). Among the patients treated with curative intent, 2 (15.4%) had been previously operated on at other institution, 4 (30.8%) had received neoadjuvant therapy, and all presented with resectable disease. The surgical margin status was R0 or R1 in all curatively treated cases. Adjuvant therapy was administered in 5 patients (38.5%), primarily radiotherapy (n = 4, 80.0%).

Oncological outcomes were evaluated in the 13 patients who underwent curative surgery, with a median follow-up duration of 24 months (IQR: 8.3). Of these, 2 patients (15.4%) had previously undergone surgical resection at another institution. The rate of local recurrence in this subgroup was 53.8% (n = 7), with a median time to recurrence of 6 months (IQR: 10.8). Similarly, the rate of distant metastasis was 53.8% (n = 7), with a median time to metastasis of 10 months (IQR: 9.0). The disease-free survival (DFS) rate was 30.8% (n = 4), while the overall survival (OS) rate was 69.2% (n = 9).
