# Supplementary material for: Succinate Dehydrogenase B (SDHB) Overexpression with Enzymatic Dysfunction Defines a Distinct Subtype of Undifferentiated Pleomorphic Sarcoma
Source: Cancer Res Commun. 2025 Oct 30;5(10):1934–45. doi: 10.1158/2767-9764.CRC-25-0468 (PMC12573234; doi:10.1158/2767-9764.CRC-25-0468)
Supplement: Supplementary Table 1 [file crc-25-0468_supplementary_table_1_suppst1.docx]

**Supplementary Table 1** - Differentially expressed genes between UPS and high-grade LMS

| Gene | **logFC** | **AveExpr** | **t** | **P.Value** | **adj.P.Val** | **B** |
| --- | --- | --- | --- | --- | --- | --- |
| **NFIC** | -0.789584423237439 | 9.85583721746982 | -4.6660585018346 | 1.27135446837404e-05 | 0.0120270391011854 | 3.04831067541231 |
| **STAT1** | 1.70934989082454 | 7.67779987726033 | 4.48488591618392 | 2.50911803223618e-05 | 0.0120270391011854 | 2.45239696777146 |
| **PPP1R13L** | -1.82984844077045 | 6.42612631774074 | -4.48740428565049 | 2.48574777197426e-05 | 0.0120270391011854 | 2.44230457271796 |
| **MAFB** | -1.20505318351186 | 7.96666758464597 | -4.3697395307354 | 3.83786404151358e-05 | 0.013082367784934 | 2.06379964871452 |
| **SDHD** | 1.059339669051 | 6.21377881179977 | 4.24624566850602 | 6.01514716959258e-05 | 0.013082367784934 | 1.64860486804697 |
| **BCAR1** | -0.916175634126613 | 7.09177556659966 | -4.21702103692396 | 6.68335435202448e-05 | 0.013082367784934 | 1.56626603886904 |
| **SP2** | -0.69359254201985 | 7.8139211508565 | -4.18824794918991 | 7.41088290066368e-05 | 0.013082367784934 | 1.46673965215019 |
| **FOXO4** | -0.962971458356956 | 6.79906087671984 | -4.17452813287969 | 7.78411833354592e-05 | 0.013082367784934 | 1.4295063347586 |
| **GNA12** | 0.884818280232242 | 6.24666348018837 | 4.12487634688578 | 9.2921968862282e-05 | 0.013082367784934 | 1.26517173990552 |
| **SDHC** | 0.96469603609239 | 7.92909864628879 | 4.10801208008077 | 9.86566121906347e-05 | 0.013082367784934 | 1.205319930679 |
| **MRVI1** | -1.40317465136939 | 6.90771964013597 | -4.03766343913373 | 0.000126468462537322 | 0.0134968252328725 | 0.99455365309176 |
| **DCAF12** | 1.04845431977809 | 6.48046987078603 | 4.03478162622433 | 0.000127755177330249 | 0.0134968252328725 | 0.986581195842436 |
| **ALOX12** | -1.05560818070521 | 6.79124530293581 | -4.0267637439263 | 0.000131401636481374 | 0.0134968252328725 | 0.96118142024031 |
| **CDK2** | 0.957753438981249 | 5.59209412999343 | 3.9339151282697 | 0.000181610719333151 | 0.0159918024729759 | 0.654646742801542 |
| **MALAT1** | -0.838050666754233 | 12.4567926229887 | -3.98169342015621 | 0.000153833792908748 | 0.0147475329468519 | 0.601171495377745 |
| **PSME1** | 0.916464511001152 | 7.63706827543938 | 3.89069144573472 | 0.000210830365549874 | 0.0159918024729759 | 0.523839166374308 |
| **ZEB1** | -1.25395266293234 | 5.07097883876647 | -3.89004964839513 | 0.000211296416541406 | 0.0159918024729759 | 0.488550038378418 |
| **PARP12** | 1.32167593196179 | 4.23223254928642 | 3.90113258224261 | 0.000203385222509571 | 0.0159918024729759 | 0.409523701816112 |
| **FBXW11** | 0.947713948809528 | 5.1460530867747 | 3.84600063469755 | 0.00024574975306906 | 0.0176694072456654 | 0.36736542687013 |
| **ELF4** | 0.723924242102099 | 8.85448493792498 | 3.82070855166854 | 0.00026789550627175 | 0.0183444637151799 | 0.271177837795681 |
| **PSENEN** | 1.01102953566758 | 6.75695886193859 | 3.77620882743149 | 0.00031156414292861 | 0.0184612552389121 | 0.192455209103178 |
| **PRPF8** | 1.09618469051861 | 7.27140011149265 | 3.77784495165756 | 0.000309844733984757 | 0.0184612552389121 | 0.185931968072728 |
| **IL4R** | -0.986530497173707 | 6.07832256813762 | -3.76742334291816 | 0.000320953672442839 | 0.0184612552389121 | 0.173224747830697 |
| **HDAC5** | -0.676570828491214 | 7.57194189812347 | -3.72785299537956 | 0.00036669402056659 | 0.0188262309940631 | 0.0275723245293769 |
| **TPM3** | 0.694216188836872 | 11.3755175626795 | 3.77214774518086 | 0.000315871390119234 | 0.0184612552389121 | -0.00362359774743659 |
| **ZC3H13** | -0.787798795080736 | 9.142888222286 | -3.72786429478596 | 0.000366680113322768 | 0.0188262309940631 | -0.0274837140767881 |
| **CDC6** | 1.82431862649599 | 6.26393514089737 | 3.68804204713572 | 0.000418942553414477 | 0.0188262309940631 | -0.0608173889452077 |
| **GAS1** | -1.52418631375995 | 8.51699241897329 | -3.70734927716933 | 0.000392775286557459 | 0.0188262309940631 | -0.0628476546399499 |
| **SDHB** | 0.764410407423375 | 9.02510705733615 | 3.71232230256748 | 0.000386291805152101 | 0.0188262309940631 | -0.0693821590224903 |
| **IRF1** | 1.27297577574181 | 7.23333501388461 | 3.69167012540596 | 0.000413902031566874 | 0.0188262309940631 | -0.0720496622093973 |
| **ATAD2** | 1.54602842456617 | 4.00626755112027 | 3.71617429709067 | 0.000381340050188926 | 0.0188262309940631 | -0.130004799319943 |
| **CASP1** | 1.85420409956581 | 4.12127720538774 | 3.65454228399873 | 0.000468327259386067 | 0.019807488205799 | -0.276708110242507 |
| **APH1A** | 0.581090741306348 | 8.25216939630856 | 3.62723705738726 | 0.000512626868176715 | 0.021061641041089 | -0.293786118555732 |
| **DDX21** | 0.994664813116282 | 5.70929447492488 | 3.60066669496652 | 0.00055953621146423 | 0.0218976507729986 | -0.311205882892391 |
| **TCL1A** | -2.97087493747756 | -1.79053088752711 | -4.10399013066376 | 0.000100073745225503 | 0.013082367784934 | -0.313873212014557 |
| **CNN1** | -2.22374270952542 | 5.50285040591746 | -3.50920766275827 | 0.000754108360327525 | 0.0264489712719752 | -0.56902233659557 |
| **LPP** | -0.655660519492356 | 10.4622602159627 | -3.56133166060312 | 0.000636525052651795 | 0.0240874480450863 | -0.598332375619616 |
| **PVRIG** | -1.06694254408491 | 5.8573433177247 | -3.46838867979067 | 0.000860250108755257 | 0.0274897701420013 | -0.681663797165902 |
| **CD244** | 2.65930632921841 | 1.71320393218464 | 3.67535404065576 | 0.000437033550352471 | 0.0190440680426319 | -0.683594133088697 |
| **JAK1** | 0.65614998541497 | 12.1126984491325 | 3.54465046704332 | 0.000672119935570723 | 0.0243704299529685 | -0.748521223813842 |
| **CDK4** | 0.87345473634057 | 11.0481320094943 | 3.4748031488797 | 0.000842683477142154 | 0.0274897701420013 | -0.888439993732616 |
| **ACTG2** | -2.6359879599009 | 4.68324949873384 | -3.3872922275652 | 0.00111437323395613 | 0.0333847648006025 | -0.923635082785917 |
| **LY6E** | 1.13708408653432 | 8.03043097194872 | 3.38942957096463 | 0.00110685092309864 | 0.0333847648006025 | -0.976196471230746 |
| **CXCL11** | 2.49141070213015 | 2.2690675813549 | 3.50109567787524 | 0.000774161770433262 | 0.0265058244257865 | -0.97647470619912 |
| **ARHGAP15** | 3.43187615413257 | 1.51736308621025 | 3.54202198108126 | 0.000677897912460875 | 0.0243704299529685 | -1.01342520317702 |
| **C5** | 3.5078087410777 | 0.598295495425664 | 3.59855700781313 | 0.0005634305136307 | 0.0218976507729986 | -1.02012080246691 |
| **CLTC** | 0.511164864309173 | 12.3423770107446 | 3.44804869951008 | 0.000918270080139964 | 0.0287059212008971 | -1.05184162054194 |
| **FUCA1** | 1.03798838745945 | 5.59456238700002 | 3.32613196733803 | 0.00135118957201456 | 0.0378602976043414 | -1.06536251235048 |
| **NOS1AP** | -2.45732542633202 | 1.9530615040011 | -3.48254423356801 | 0.000821935371830395 | 0.0274870480160955 | -1.07288547900803 |
| **CXCL10** | 1.91976595871566 | 5.19974482256587 | 3.30707438057998 | 0.00143416558204807 | 0.0378729290261139 | -1.11552401419339 |
| **MEGF9** | -0.578896185674568 | 8.82547273090993 | -3.33098686349743 | 0.00133078489236414 | 0.0378602976043414 | -1.17736835702136 |
| **KDR** | 1.58360578160831 | 5.26574865641108 | 3.27655099448382 | 0.00157710768051356 | 0.0397873832382194 | -1.19468191913993 |
| **NXPH3** | -1.41398602160162 | 3.75280976665206 | -3.30387703484765 | 0.00144854735496263 | 0.0378729290261139 | -1.20982035859615 |
| **SKP1** | 0.892900007857872 | 6.91753203598304 | 3.28036250496056 | 0.0015585542491817 | 0.0397873832382194 | -1.23593015327085 |
| **RGCC** | -0.956792626419003 | 5.96359606347053 | -3.25184259355131 | 0.00170250101123396 | 0.0414948551551598 | -1.27133192345584 |
| **DHH** | -1.10162471330505 | 2.44936375831611 | -3.34741048917319 | 0.00126388611787112 | 0.0370911885203809 | -1.29166857229547 |
| **CCL8** | 2.00832830371516 | 3.73866146947965 | 3.22463307685507 | 0.00185137419629623 | 0.042939937004419 | -1.40097944845372 |
| **NKD1** | -2.16578714451691 | 4.52967115004595 | -3.1827254890963 | 0.00210470113462438 | 0.0465624651013824 | -1.44828305697936 |
| **PSMB8** | 0.92145054852312 | 6.55759138665133 | 3.18634477040592 | 0.00208160555928537 | 0.0465624651013824 | -1.47173801110651 |
| **SS18** | 0.56681038858023 | 10.9421447558634 | 3.26687784383266 | 0.00162512890897367 | 0.0402919891569679 | -1.48156499708501 |
| **APOBR** | -1.14393285048069 | 9.82998442272165 | -3.22641851570226 | 0.0018412431489761 | 0.042939937004419 | -1.52851940827563 |
| **WNT16** | -2.50233022533956 | 0.970347960667151 | -3.30654005429618 | 0.00143655962509844 | 0.0378729290261139 | -1.58237186493538 |
| **YY1** | -0.458134303470864 | 7.62317518370726 | -3.16005079335066 | 0.00225493397349069 | 0.0483969411026809 | -1.59165947529959 |
| **NEAT1** | -0.695691659265238 | 12.0076595692596 | -3.2335635231698 | 0.00180121795092807 | 0.042939937004419 | -1.64686559194275 |
| **CXCR5** | -1.58756790938665 | 1.1947580572389 | -3.19652340335262 | 0.00201791717828504 | 0.046059760355141 | -1.77959255527872 |
| **CDX2** | -2.38894959864585 | -3.35859924892717 | -3.32193193884522 | 0.00136907891197897 | 0.0378602976043414 | -1.98119868285315 |
| **IHH** | -2.10299166365811 | -1.11174763770739 | -3.16345595024607 | 0.00223175098824757 | 0.0483969411026809 | -2.04435208258442 |
