# Supplementary material for: Succinate Dehydrogenase B (SDHB) Overexpression with Enzymatic Dysfunction Defines a Distinct Subtype of Undifferentiated Pleomorphic Sarcoma
Source: Cancer Res Commun. 2025 Oct 30;5(10):1934–45. doi: 10.1158/2767-9764.CRC-25-0468 (PMC12573234; doi:10.1158/2767-9764.CRC-25-0468)
Supplement: Supplementary Table 2 [file crc-25-0468_supplementary_table_2_suppst2.docx]

**Supplementary Table 2** - Differentially expressed genes between UPS and DDLPS

| Gene | **logFC** | **AveExpr** | **t** | **P.Value** | **adj.P.Val** | **B** |
| --- | --- | --- | --- | --- | --- | --- |
| **MDM2** | -3.43687632132414 | 8.29318909997119 | -13.3168093110995 | 1.12693473209516e-21 | 1.62053214475284e-18 | 38.2628546199005 |
| **CDK4** | -2.29283312838111 | 11.0481320094943 | -9.47684030878701 | 1.44554429982583e-14 | 1.03934635157477e-11 | 22.8024759412912 |
| **CCNB1** | 2.09232664455016 | 6.16107552618726 | 7.2092996753437 | 3.32441952102088e-10 | 1.59350509040934e-07 | 12.965833102946 |
| **CLTC** | 0.973536472632007 | 12.3423770107446 | 6.82335493341451 | 1.78981982825169e-09 | 6.43440228256481e-07 | 11.3022428777154 |
| **CCND2** | -3.04904442754487 | 7.51636847893976 | -6.68786625725138 | 3.21774833852623e-09 | 9.25424422160144e-07 | 10.8631051249955 |
| **WHSC1** | 1.38230927351389 | 9.96797152236539 | 6.35518001830884 | 1.34246513058173e-08 | 3.21744142962755e-06 | 9.44405660524049 |
| **ANXA2** | 1.54081426376126 | 7.57648312659572 | 6.12511576914354 | 3.56391389773567e-08 | 7.3212974070627e-06 | 8.56552375023907 |
| **KIF23** | 1.94489456591771 | 7.0699488629083 | 6.04385982191768 | 5.01865976039145e-08 | 9.02104091930363e-06 | 8.2437856021799 |
| **TPM3** | 1.05259703273507 | 11.3755175626795 | 5.94433704921053 | 7.6177234464948e-08 | 1.21714292400661e-05 | 7.69873500066388 |
| **ZMAT3** | -1.45812405562422 | 7.00743750286118 | -5.85012571717193 | 1.12847714610337e-07 | 1.4752273964515e-05 | 7.47414114692098 |
| **AURKA** | 2.58473371922525 | 5.46159656917431 | 5.85402622526248 | 1.11031123429752e-07 | 1.4752273964515e-05 | 7.46109664210979 |
| **ELF4** | 1.05657748103714 | 8.85448493792498 | 5.8044031389142 | 1.36456217441891e-07 | 1.63520033901199e-05 | 7.25005007755985 |
| **NRAS** | 1.24171341270049 | 9.24542481988066 | 5.50243151215441 | 4.72146984296236e-07 | 5.22267202629221e-05 | 6.04131786334106 |
| **CCNB2** | 1.87281831899367 | 4.96628794243036 | 5.41219494499644 | 6.8089341572695e-07 | 6.99374808439539e-05 | 5.75005899623829 |
| **RRM2** | 1.92464976834381 | 6.53079159814559 | 5.38093905166572 | 7.72529026100944e-07 | 7.40597826355439e-05 | 5.66092591562009 |
| **CDCA5** | 1.49176657234836 | 7.46693861182796 | 5.21328175867733 | 1.51309148234315e-06 | 0.000134856390425326 | 4.99891193804802 |
| **TOP2A** | 1.67180795332205 | 6.17158009105779 | 5.20015578260586 | 1.59426887150941e-06 | 0.000134856390425326 | 4.98724534661351 |
| **CDC20** | 2.07806572687036 | 5.74632963522538 | 5.16205985049827 | 1.85480324786655e-06 | 0.000146621550350762 | 4.85095173774474 |
| **CDKN3** | 1.74263160168204 | 6.37308000991613 | 5.13290071671133 | 2.0819722334271e-06 | 0.000149693803583409 | 4.73157540181996 |
| **KIF2C** | 3.0481937988861 | 5.53678481188073 | 5.04590274877661 | 2.93395306192605e-06 | 0.000191773841047712 | 4.42490200665526 |
| **TPX2** | 1.79117202523813 | 5.5062293775195 | 5.01660822301251 | 3.29130257799959e-06 | 0.000205777961181018 | 4.31835878723997 |
| **SOX17** | -1.41243873435967 | 5.49637862514277 | -4.94999386850339 | 4.26963223512162e-06 | 0.000255822131421037 | 4.07749122849891 |
| **ECT2** | 1.53522096923241 | 5.88834794509555 | 4.8686523225608 | 5.8542778342112e-06 | 0.000301929435689813 | 3.77859754344779 |
| **ZBTB16** | -3.02969522450831 | 7.55188795965377 | -4.87651963200134 | 5.67884588081095e-06 | 0.000301929435689813 | 3.74286482301715 |
| **CDK2** | 1.11862682100119 | 5.59209412999343 | 4.84487130458789 | 6.4173250603003e-06 | 0.000301929435689813 | 3.69959253059801 |
| **NEK2** | 4.02729494171626 | 0.493595759371475 | 5.06509597756555 | 2.72070229525967e-06 | 0.00018630332859921 | 3.67232764554546 |
| **UBA7** | -1.2141914687894 | 6.41468546013424 | -4.83474309322513 | 6.67285341902151e-06 | 0.000301929435689813 | 3.63677095944523 |
| **SHISA5** | -0.66930640115519 | 8.80976332935458 | -4.84326036413137 | 6.45732012045044e-06 | 0.000301929435689813 | 3.5637039565271 |
| **DDX10** | 0.983516149902752 | 8.59542403277383 | 4.83295924997945 | 6.71887478586511e-06 | 0.000301929435689813 | 3.5379682151666 |
| **TCL1A** | -3.60769750911783 | -1.79053088752711 | -5.15108792754164 | 1.93728056791689e-06 | 0.000146621550350762 | 3.53772985043737 |
| **PRKCA** | 1.10090652009618 | 9.68726634838434 | 4.8394045725602 | 6.5540381910949e-06 | 0.000301929435689813 | 3.49767979845207 |
| **BIRC5** | 1.48648005239151 | 7.04331007026114 | 4.79554632420369 | 7.75858261634734e-06 | 0.000338086115221439 | 3.46786664095592 |
| **HLF** | -3.68052703036482 | 3.2243431277507 | -4.78759373921531 | 7.99899142414855e-06 | 0.000338310284350753 | 3.33122121520384 |
| **PTTG1** | 1.29200631473635 | 5.06175958842315 | 4.7360067155337 | 9.74432130418084e-06 | 0.000365568902310812 | 3.3161025407366 |
| **MAML3** | -1.13913797793737 | 7.79005774710433 | -4.75377675503408 | 9.10489143247562e-06 | 0.000361446903234324 | 3.28707632276831 |
| **UBE2C** | 1.85637443983706 | 5.10165107462133 | 4.72513379071813 | 1.0156909908887e-05 | 0.000365568902310812 | 3.27851506274708 |
| **BRCA1** | 1.46394547249812 | 9.65789139531925 | 4.75772508936888 | 8.96846647461913e-06 | 0.000361446903234324 | 3.19995262906164 |
| **ABL2** | 1.08776685336014 | 9.34131965458619 | 4.72482647716685 | 1.01688150851408e-05 | 0.000365568902310812 | 3.09935851674455 |
| **TCF7L1** | -1.59380066544909 | 7.94820885333099 | -4.65467696910967 | 1.32734546651691e-05 | 0.000465542141671054 | 2.92396571863269 |
| **RAC1** | 0.850385024825114 | 7.49529250357203 | 4.62902588729791 | 1.46246803422395e-05 | 0.000489076519352104 | 2.85161464493739 |
| **ANLN** | 2.21358141867246 | 6.64769665784129 | 4.60177019883416 | 1.62068121542973e-05 | 0.000529668088133625 | 2.79391433437805 |
| **CEACAM5** | -3.59123866796717 | -1.94566212971336 | -4.88258918292219 | 5.54701707898487e-06 | 0.000301929435689813 | 2.74084219158653 |
| **MAGEA3** | 6.35383561113722 | 1.58931441429007 | 4.64081873071703 | 1.39876348615455e-05 | 0.000478909974545295 | 2.56225785254754 |
| **NUF2** | 2.88900834655358 | 3.39777460745 | 4.49307712400218 | 2.43387493342437e-05 | 0.000777758256503166 | 2.39204728244211 |
| **CENPF** | 1.36054437279211 | 6.74228526996646 | 4.47769725146477 | 2.57700630663617e-05 | 0.00080559458020496 | 2.35452436067173 |
| **MEST** | -2.94502925489403 | 4.85135156753427 | -4.45261916886218 | 2.82806363577383e-05 | 0.000813985470365727 | 2.34361833347357 |
| **FOXM1** | 1.15845573899596 | 6.59134509552175 | 4.46617496489004 | 2.68955081474972e-05 | 0.000813985470365727 | 2.32284397208683 |
| **CEP55** | 1.66270193698569 | 4.14237818936096 | 4.44705713875397 | 2.88687475581725e-05 | 0.000813985470365727 | 2.30487555154276 |
| **GJA4** | -1.16456594059534 | 6.53568928266332 | -4.44933075052264 | 2.86269249534848e-05 | 0.000813985470365727 | 2.26767532518626 |
| **RBP7** | -1.97808651177442 | 5.16052157050578 | -4.42907373910943 | 3.08524771341003e-05 | 0.000837091738091249 | 2.26177475404237 |
| **TYMS** | 1.1159138073786 | 7.98575294821864 | 4.45372554662049 | 2.81650439274219e-05 | 0.000813985470365727 | 2.21199352344049 |
| **CDX2** | -3.30856083563814 | -3.35859924892717 | -4.74822636928026 | 9.30009417223227e-06 | 0.000361446903234324 | 2.14338529729037 |
| **ORC6** | 2.14759682313583 | 3.86778865088096 | 4.38039526024092 | 3.69073968365519e-05 | 0.000931102397385292 | 2.07054924388497 |
| **TP53** | -1.50950490106517 | 9.2489821895617 | -4.42993431803163 | 3.07546107603376e-05 | 0.000837091738091249 | 2.05339893759585 |
| **BTG1** | -0.989759559639765 | 8.11983632953776 | -4.4091416280592 | 3.32054624201259e-05 | 0.000884249165928538 | 2.05020410542932 |
| **ZNF608** | -1.43373770312096 | 5.77728143507703 | -4.34086526931 | 4.26562185133743e-05 | 0.00105758003831435 | 1.9413361339008 |
| **SFRP1** | -3.9552061137872 | 4.83147470612725 | -4.31047292049684 | 4.7655806660449e-05 | 0.00112342704881518 | 1.87063651245789 |
| **FLNA** | 0.940010155872242 | 10.4533373946454 | 4.39033865825046 | 3.55838368059424e-05 | 0.00093035558776264 | 1.83775048260095 |
| **NIN** | 0.716249474555887 | 11.2502589788451 | 4.38227280191176 | 3.66538829067259e-05 | 0.000931102397385292 | 1.75575376160915 |
| **COL18A1** | -1.22817418215843 | 7.0525427017944 | -4.27986313134791 | 5.32615087510613e-05 | 0.00122739193885622 | 1.65817558683894 |
| **CDCA7L** | 1.89856705765824 | 5.32001522067576 | 4.2068988181914 | 6.93106999653896e-05 | 0.00151013312954894 | 1.51728373654644 |
| **CBL** | 0.608893897751194 | 9.43495760638122 | 4.27722664240994 | 5.37730821612949e-05 | 0.00122739193885622 | 1.51171984033877 |
| **STIL** | 1.11582966196778 | 6.82276876037446 | 4.21967263272838 | 6.61988832703788e-05 | 0.00147557628772544 | 1.46807537411121 |
| **PLEK2** | 4.30534255145317 | 0.588127128773754 | 4.31758661104576 | 4.64371469805235e-05 | 0.00111294362263321 | 1.46728695218775 |
| **SSPO** | -2.58268261592189 | 5.11161085466355 | -4.15905138396299 | 8.22689697096523e-05 | 0.00173974674180118 | 1.36907503451292 |
| **MEGF9** | -0.704205396839651 | 8.82547273090993 | -4.21758315974122 | 6.66985109194394e-05 | 0.00147557628772544 | 1.3501561337423 |
| **DTX1** | -2.51873105530207 | 3.78425152972448 | -4.13701300461795 | 8.89949608753857e-05 | 0.00177742713526117 | 1.2924880789786 |
| **CNTNAP2** | -4.92483878148736 | -1.22668120237324 | -4.32227941445264 | 4.56497632011444e-05 | 0.00111261626242789 | 1.28549499345275 |
| **EMCN** | -1.85684173124978 | 3.47133801066378 | -4.14030123234929 | 8.79588267720773e-05 | 0.00177742713526117 | 1.28451873012508 |
| **CDCA8** | 1.53216418124137 | 6.34857896475686 | 4.14435574541503 | 8.66972204510507e-05 | 0.00177742713526117 | 1.24802658906493 |
| **MELK** | 2.42940078477617 | 4.39987719884064 | 4.10981159011044 | 9.80288270538686e-05 | 0.00188346292284841 | 1.22301263834308 |
| **ARG2** | 2.47698271081156 | 2.79907484131873 | 4.131480153422 | 9.07649654614423e-05 | 0.00178794548402129 | 1.19787712568063 |
| **TGFB1** | 0.818767204649225 | 9.69031825923995 | 4.1828122426857 | 7.55664547853159e-05 | 0.00162185913404902 | 1.17269919860625 |
| **CX3CL1** | -1.57380694808997 | 4.37737454936791 | -4.07010719426765 | 0.000112815430602868 | 0.00207985370778107 | 1.09766532731182 |
| **SPRY4** | -1.42264084573196 | 7.76366624910483 | -4.10922379871957 | 9.82334625964053e-05 | 0.00188346292284841 | 1.04918776452387 |
| **PRMT5** | 0.721423699617493 | 10.2386843571671 | 4.14937640950808 | 8.51591254083612e-05 | 0.00177476554111918 | 1.0234595362917 |
| **ABI1** | 0.589889341546361 | 7.65939442898955 | 4.09042601714651 | 0.00010499891630219 | 0.00196088885250063 | 0.99219842811368 |
| **CEBPA** | -2.0184382685675 | 3.8665713809092 | -4.01689367402131 | 0.000136027856713635 | 0.00238546412139277 | 0.9239105179441 |
| **IFI16** | 0.841496339137113 | 7.17012536446893 | 4.05138611411388 | 0.000120510274846529 | 0.00219359209151023 | 0.889221094311413 |
| **TFG** | 0.743943991578584 | 9.92361777276792 | 4.0967653502981 | 0.000102668737174537 | 0.00194260057969717 | 0.867452448392681 |
| **CD36** | -1.90046998940801 | 7.07763823172717 | -4.03816844804607 | 0.000126244264584566 | 0.00226924065590757 | 0.851145349718682 |
| **BUB1B** | 2.31538481278129 | 4.34223097799566 | 3.95010436355237 | 0.000171699720503224 | 0.00280589688332397 | 0.724039745585332 |
| **EDNRB** | -1.40448357215831 | 5.80590005369478 | -3.96823601950368 | 0.000161215363296115 | 0.00275985348118825 | 0.715543200434919 |
| **SDHB** | 0.797064893128686 | 9.02510705733615 | 4.02959038615312 | 0.000130104840409372 | 0.00230976247541577 | 0.707065540609236 |
| **CD79B** | -1.54640903120178 | 3.04660188769957 | -3.95008717616175 | 0.000171709962261829 | 0.00280589688332397 | 0.679798779632503 |
| **HMGA2** | -3.56393816851271 | 6.73720236397679 | -3.95709128503346 | 0.000167584457376815 | 0.00280216801985883 | 0.609489021568163 |
| **BAG1** | 0.942190042594286 | 7.03604331038156 | 3.9628461115673 | 0.000164266090229685 | 0.00277899573823868 | 0.608663076206334 |
| **PTPRB** | -1.6580993658271 | 4.47299925907608 | -3.91048149081594 | 0.00019693310236014 | 0.00300885351606468 | 0.600441846968578 |
| **FGFR1OP** | 0.829094615908327 | 8.79237545295724 | 3.99140464992971 | 0.000148709670231858 | 0.00257643982883628 | 0.598350163638586 |
| **ODC1** | 1.13485461567255 | 7.21899801246465 | 3.93327949915497 | 0.000182010826669937 | 0.00290812854168189 | 0.502555085591766 |
| **RRAGC** | 1.01787562533637 | 6.68984637110609 | 3.9077803995404 | 0.00019877683172889 | 0.00300885351606468 | 0.454554995704194 |
| **PBK** | 2.34533142498986 | 3.3217000496932 | 3.86687345572315 | 0.000228802722350053 | 0.00329018314739377 | 0.451150116292498 |
| **ANXA1** | 0.934227085358573 | 8.69789629853966 | 3.9425698229985 | 0.000176246060577564 | 0.00284766106865772 | 0.445846190931237 |
| **COL15A1** | -1.56573358078258 | 8.22882430859266 | -3.92218814415196 | 0.000189129938392268 | 0.00292706231996158 | 0.410911737049359 |
| **FAP** | 2.63757428823055 | 5.44389281084811 | 3.85772117773592 | 0.000236091243608216 | 0.0033613783000853 | 0.392721213238443 |
| **SOCS1** | -1.20957620446082 | 7.3906536326869 | -3.89380131730105 | 0.000208585987525586 | 0.00309223350579168 | 0.366186653154318 |
| **CYLD** | 0.824769246507302 | 9.36599616675823 | 3.92687832284302 | 0.000186087527439602 | 0.00292706231996158 | 0.345988226481082 |
| **SDHC** | 0.879171414761639 | 7.92909864628879 | 3.8863153161425 | 0.000214027784985974 | 0.00310880762434172 | 0.31284134022158 |
| **P2RY8** | -1.41270844848827 | 3.61080421098569 | -3.81404134461128 | 0.000274043597732109 | 0.00375309231941689 | 0.306241929174495 |
| **DDIT4** | -1.26848959112528 | 8.49303839063792 | -3.88704120884144 | 0.000213494219336489 | 0.00310880762434172 | 0.28073239966534 |
| **MAGEA6** | 4.90830035040555 | 0.319771394873463 | 3.89469406506388 | 0.000207945933683727 | 0.00309223350579168 | 0.27579782194208 |
| **RHOA** | 0.522956699073175 | 10.5294436249095 | 3.92192450623122 | 0.000189302361443969 | 0.00292706231996158 | 0.249198930504259 |
| **UBE2T** | 1.26428081139244 | 4.7759738516116 | 3.78814341441324 | 0.000299228189539234 | 0.00402140314539643 | 0.216911265592535 |
| **CCNA2** | 1.52766779571648 | 4.61642658112547 | 3.75626526938839 | 0.000333268544247932 | 0.0043966987764085 | 0.127399739315665 |
| **APH1A** | 0.588710029913238 | 8.25216939630856 | 3.81702042734559 | 0.000271279990673127 | 0.00375309231941689 | 0.072866709925294 |
| **NFIC** | -0.626949285785596 | 9.85583721746982 | -3.85083604992552 | 0.000241720122672831 | 0.00340777976866207 | 0.0655100267123885 |
| **LRRC15** | 3.21449572673313 | 6.36894823259401 | 3.73864330473226 | 0.00035363988712877 | 0.00456001840809545 | -0.0522317684755 |
| **SNW1** | 0.71607516063691 | 6.51772116224886 | 3.73736629715405 | 0.000355161378099228 | 0.00456001840809545 | -0.0684484794273992 |
| **ATIC** | 0.79052172524664 | 9.40988703891627 | 3.79416868333644 | 0.00029317880595044 | 0.00397727474487483 | -0.0834913309982079 |
| **NOTCH4** | -1.04605242677878 | 10.5180359050849 | -3.81669783340554 | 0.000271577960971047 | 0.00375309231941689 | -0.0897737843431097 |
| **PAFAH1B2** | 0.711371477125049 | 7.83157130647687 | 3.73820483328403 | 0.000354161602150683 | 0.00456001840809545 | -0.150237287608659 |
| **BCL2** | -1.03412182275148 | 10.8232205867547 | -3.78267003737772 | 0.000304826614061652 | 0.00405870991685792 | -0.22016032170892 |
| **KMT5A** | 0.715843869676683 | 6.97457438442478 | 3.6829398742777 | 0.000426130010108615 | 0.00542278720828485 | -0.27047189982518 |
| **PVRIG** | -1.06581121150692 | 5.8573433177247 | -3.65101915954171 | 0.000473831409111564 | 0.00597692602019674 | -0.274744759251115 |
| **TDRD7** | 2.04223885251096 | 3.07426554457914 | 3.59499331725836 | 0.000570067289524227 | 0.00694709120623592 | -0.33715340729512 |
| **PPARG** | -1.56487204255917 | 6.69288535824364 | -3.6208406565452 | 0.000523565456897678 | 0.00643493270956292 | -0.43946972555853 |
| **ARID3A** | -1.0374864730222 | 4.48967375537047 | -3.56019887924892 | 0.000638884514459888 | 0.00759269365118446 | -0.444924244890665 |
| **PAGE5** | 4.92537854161681 | -0.914344196772872 | 3.64155724777841 | 0.000488919748460731 | 0.00611362259379592 | -0.463954143497072 |
| **WBSCR17** | -3.68399822855932 | 3.55138461170312 | -3.54085551347122 | 0.000680477110732919 | 0.00795549662791819 | -0.479387324630422 |
| **MCM4** | 1.24684455842201 | 5.48081375041509 | 3.55459815533236 | 0.000650672590841826 | 0.00766940316090612 | -0.529371328771038 |
| **CHEK1** | 1.82706636049403 | 4.41465354627502 | 3.52171978930035 | 0.000724138960835521 | 0.00833049460545183 | -0.551986866592586 |
| **BUB1** | 1.1022246454636 | 4.83284892492767 | 3.50494524351866 | 0.000764583249664654 | 0.0086562558298236 | -0.624278229047891 |
| **LAMB1** | -0.950671596006534 | 8.1525155608191 | -3.58062573387698 | 0.000597584761892011 | 0.00717167172791234 | -0.654407999578964 |
| **ATM** | 0.703019238920122 | 10.6486538320452 | 3.62750603926713 | 0.000512171669556789 | 0.00634916259329882 | -0.694160003377232 |
| **SDHD** | 0.831059231930197 | 6.21377881179977 | 3.50333042631485 | 0.000768587513037279 | 0.0086562558298236 | -0.749252578543153 |
| **WT1** | -3.79259073372004 | 2.77319785943346 | -3.43199868106804 | 0.000966641980764635 | 0.0103733669279071 | -0.790456265594206 |
| **EXO1** | 2.00279045347618 | 3.93328904255895 | 3.42652197963517 | 0.00098369123167372 | 0.0104252050750226 | -0.802191389845009 |
| **CTSV** | 3.01706855863633 | -1.26588062403566 | 3.50255590772806 | 0.000770515122543408 | 0.0086562558298236 | -0.827127693226366 |
| **PSMB8** | 0.957522222412327 | 6.55759138665133 | 3.46973382516442 | 0.000856537857654673 | 0.00940230106341541 | -0.879560518427239 |
| **RBX1** | 0.668416650745774 | 6.93118955778652 | 3.47814215303098 | 0.000833674066573849 | 0.00926049963678907 | -0.884886176439246 |
| **EPS15** | 0.638653119364228 | 11.359797274409 | 3.58017376290258 | 0.000598470519714521 | 0.00717167172791234 | -0.89385111361774 |
| **TPM1** | 0.984038645729397 | 9.88373094770697 | 3.53222966518331 | 0.000699840544464348 | 0.008115892765643 | -0.930600283773695 |
| **RAD51** | 1.48821284725954 | 3.97773722597828 | 3.3784427348525 | 0.00114603452456597 | 0.0117974911662533 | -0.935987908699992 |
| **CREB1** | 0.444782461515555 | 7.7000475296914 | 3.47683874289277 | 0.000837180078430166 | 0.00926049963678907 | -0.93807438849456 |
| **STEAP1** | 2.68974886741476 | 3.42838428831222 | 3.36832267888667 | 0.001183280005808 | 0.0119827932982528 | -0.952494274094819 |
| **ITPKB** | -0.802228956353141 | 6.44507019634005 | -3.4257961040893 | 0.000985972107234407 | 0.0104252050750226 | -0.998092043761567 |
| **FZD7** | -1.18702904209053 | 7.4705948003603 | -3.44853417807802 | 0.000916843182958361 | 0.00991293606837686 | -1.00782836221529 |
| **CDKN1A** | -0.934945504323371 | 8.32124008617721 | -3.46366061469204 | 0.000873419375451717 | 0.0095149777416634 | -1.01638100639175 |
| **FOXO3** | -0.736842174992096 | 6.44339480572688 | -3.41326150747668 | 0.00102615483724575 | 0.0107708807004335 | -1.03435341167893 |
| **MRE11A** | 0.940064811067709 | 4.83310862762722 | 3.34371012978278 | 0.00127867673212883 | 0.012594090005488 | -1.08108591401449 |
| **ATAD2** | 1.3039039217806 | 4.00626755112027 | 3.30456884525532 | 0.00144542412661026 | 0.0134098057681649 | -1.13811953581787 |
| **FANCD2** | 1.7801577370913 | 2.92143842026188 | 3.29843804237616 | 0.00147332389743861 | 0.0135307841291667 | -1.1387554121179 |
| **CEACAM8** | -2.27134927482478 | 1.48590001137227 | -3.3128165477166 | 0.00140867342163965 | 0.0133267919757751 | -1.15161955519393 |
| **MOCOS** | 1.94536880612763 | 3.42585577046377 | 3.27525453679991 | 0.00158346560321777 | 0.0140184791307432 | -1.20020557113246 |
| **FOXO1** | -1.11347908851806 | 6.64283308267774 | -3.36122910747456 | 0.00121006343550875 | 0.0120838279184832 | -1.20236225887212 |
| **SPARCL1** | -1.22518051943253 | 5.55364086163782 | -3.313995711957 | 0.00140349154491279 | 0.0133267919757751 | -1.22843962661701 |
| **COL4A2** | -0.866119440104926 | 8.52492035720122 | -3.3932915902232 | 0.00109337991072496 | 0.0113933355914674 | -1.23827710703531 |
| **NOS1AP** | -2.19990250795027 | 1.9530615040011 | -3.25276805288032 | 0.00169764049786503 | 0.0142760645376018 | -1.28094542856102 |
| **MAGEA12** | 4.19685564643552 | 0.032061366504364 | 3.2771058123032 | 0.00157439414130236 | 0.0140184791307432 | -1.29874992263145 |
| **ACVRL1** | -1.07168675302581 | 8.78779244185615 | -3.37512575350301 | 0.00115811878802923 | 0.011811168916213 | -1.3119177014528 |
| **PAK2** | 0.559817040112623 | 6.84128892990554 | 3.32222195768121 | 0.00136783650053966 | 0.0131569352537161 | -1.330660594277 |
| **COL4A1** | -0.807975072661858 | 9.27987636861398 | -3.37774307827808 | 0.00114857354887028 | 0.0117974911662533 | -1.34407831321242 |
| **CDC45** | 2.1871105422263 | 5.60932646360077 | 3.2682264619683 | 0.00161835251300352 | 0.0140192223716811 | -1.36188662322779 |
| **LMO4** | 0.873246160661167 | 7.50042981347622 | 3.32703505301055 | 0.0013473718484602 | 0.0130913562032822 | -1.36274586563694 |
| **IRF4** | -1.58061895387024 | 5.76755311380368 | -3.27328973498627 | 0.00159314700446633 | 0.0140184791307432 | -1.36419787057604 |
| **BRCA2** | 0.937117212439533 | 9.28425829436263 | 3.36541543757023 | 0.0011941887338027 | 0.0120086950993587 | -1.38040708026672 |
| **BLVRA** | 0.638914347582419 | 6.05783765375503 | 3.27043350576998 | 0.00160731995593755 | 0.0140184791307432 | -1.40308321109276 |
| **DLL4** | -0.873153667266717 | 7.37247977650118 | -3.3080905604161 | 0.00142962294122794 | 0.0134098057681649 | -1.40883290549627 |
| **RGCC** | -0.911504431991517 | 5.96359606347053 | -3.26317931382896 | 0.00164384976414327 | 0.0140206362467929 | -1.41324451125457 |
| **EZH2** | 0.985660158493691 | 6.95285864037927 | 3.29553891805329 | 0.00148669255383055 | 0.0135307841291667 | -1.41539933602414 |
| **SOX11** | -2.20510768821388 | 5.11979055678222 | -3.22622991216077 | 0.00184231087673395 | 0.0153135435881123 | -1.42961184682809 |
| **DCAF12** | 0.810155336626987 | 6.48046987078603 | 3.27019312824774 | 0.00160851812000878 | 0.0140184791307432 | -1.44659737108155 |
| **TNFRSF1A** | -0.486813209341081 | 9.91110533381927 | -3.3578370685507 | 0.00122307167915091 | 0.012129497066338 | -1.45077855123054 |
| **LDHB** | 0.922866420522018 | 9.16110216127409 | 3.33663863507401 | 0.00130739617700697 | 0.0127893585206533 | -1.4540517566005 |
| **XPO1** | 0.675890131985466 | 7.60993875438212 | 3.29705634511225 | 0.00147968113475795 | 0.0135307841291667 | -1.45528107545087 |
| **TTL** | 0.530141875853412 | 8.6450216623707 | 3.32115319400616 | 0.0013724202281345 | 0.0131569352537161 | -1.45683210559198 |
| **ADGRB3** | -2.97972534272869 | 1.69824989142076 | -3.17772970545985 | 0.00213697429997517 | 0.0170720502409127 | -1.47401980737066 |
| **CCNE1** | 1.08191425006901 | 7.70209747114747 | 3.27078422279867 | 0.00160557330581068 | 0.0140184791307432 | -1.53570271486108 |
| **SH3PXD2A** | -0.604242357373304 | 9.2055584514404 | -3.30622764499862 | 0.00143796111074073 | 0.0134098057681649 | -1.54542020946693 |
| **KIAA0040** | -2.65256111995845 | -0.153657894180022 | -3.17003652148105 | 0.00218757876746546 | 0.0173797694343389 | -1.5556042793872 |
| **PDGFB** | -0.674981390629425 | 8.45789187372517 | -3.27826324456384 | 0.00156874739673047 | 0.0140184791307432 | -1.56516114286699 |
| **AURKB** | 1.12715826230456 | 5.53036410678676 | 3.18848455230011 | 0.00206806275058316 | 0.0168015493521954 | -1.57314082083674 |
| **CDC25C** | 1.21693568355828 | 5.39249018650722 | 3.1829250530207 | 0.00210342144964365 | 0.0169806013688017 | -1.57404032449007 |
| **EPOR** | -0.837605277896282 | 6.64882776081772 | -3.2288676644236 | 0.00182743048962036 | 0.0152781688608958 | -1.57775324448136 |
| **FEV** | -2.79944509062023 | -2.9604208005247 | -3.2093708785957 | 0.00194013133460969 | 0.0158912307969956 | -1.59530505810076 |
| **MAPK1** | 0.565417252451175 | 7.23318050063985 | 3.21939101161283 | 0.00188142153653423 | 0.0155487595950357 | -1.65007900250511 |
| **APLN** | -1.14232535993445 | 4.97828342163412 | -3.13724196732109 | 0.00241611483496183 | 0.0184807081525272 | -1.65629578082064 |
| **ROBO4** | -0.859930886937599 | 9.16384971857725 | -3.26241061195986 | 0.00164776601231432 | 0.0140206362467929 | -1.66736449169246 |
| **MCM6** | 1.40253383882538 | 4.51219909480524 | 3.11500426372814 | 0.00258355559609948 | 0.0194510625507385 | -1.67373005483544 |
| **CITED4** | -1.6531487544902 | 2.1799695501392 | -3.08793224196676 | 0.00280198916480821 | 0.0203498000959303 | -1.6780699189582 |
| **CRNDE** | 1.10805866396247 | 4.71157592530515 | 3.10530505731138 | 0.00265992249032207 | 0.0197163326859956 | -1.71618277403781 |
| **BCL6** | -0.832158057835464 | 9.96889971039958 | -3.26447332393079 | 0.00163727698142662 | 0.0140206362467929 | -1.72455536995364 |
| **CA4** | -2.95643238483437 | -1.31046850071621 | -3.10944830945414 | 0.00262704670765479 | 0.0196486226375845 | -1.73628565240617 |
| **INSR** | -0.672325765254801 | 9.99306056756542 | -3.25997217057258 | 0.00166024695094334 | 0.0140437359732736 | -1.73921133818343 |
| **ITGAV** | 0.830757054205023 | 7.08415699795047 | 3.18132241161846 | 0.00211371880738213 | 0.0169806013688017 | -1.74541721714404 |
| **PSENEN** | 0.81094634189665 | 6.75695886193859 | 3.16730996830786 | 0.00220578075894112 | 0.0174280919305348 | -1.75778208627149 |
| **CTPS1** | 0.967480331997654 | 6.54881006076923 | 3.16012652254246 | 0.00225441595751903 | 0.0177150281252042 | -1.75866924485791 |
| **TNS1** | -0.825616094556832 | 6.46834973659149 | -3.14467391916101 | 0.00236245137486292 | 0.0182645434250155 | -1.79326137034542 |
| **SESN1** | -1.28370388849483 | 4.79899924115412 | -3.07785550002394 | 0.00288759730144036 | 0.0208661553742273 | -1.79614900185372 |
| **ECSCR** | -0.800579642368319 | 5.50942648433826 | -3.09689535464351 | 0.00272783357521222 | 0.0200133912303835 | -1.81768690977435 |
| **KRAS** | 0.795871348017331 | 7.31193498919579 | 3.15638917453553 | 0.00228011387016577 | 0.0177761902267082 | -1.83023527951455 |
| **CD79A** | -1.93194203747698 | 1.76662188534424 | -3.00839523161013 | 0.00354720749319575 | 0.0244061453359593 | -1.87442765109203 |
| **AFF3** | -1.60663034578257 | 6.35209578092677 | -3.10817319490858 | 0.00263712390059375 | 0.0196486226375845 | -1.8805533108497 |
| **IL7** | 2.29444439348232 | 2.36493528308722 | 2.99637396115076 | 0.0036746610767293 | 0.0246923487305455 | -1.89480546526656 |
| **PALB2** | 0.633087008786023 | 7.62713683196386 | 3.13971369259775 | 0.00239814241554693 | 0.0184413304468261 | -1.8966943662804 |
| **ITGB1** | 0.756513307459482 | 8.46280016407432 | 3.15540549757083 | 0.00228692294293534 | 0.0177761902267082 | -1.91048779367398 |
| **MAPKAPK2** | 0.640711311829005 | 7.63402349110721 | 3.12811720682324 | 0.00248355690506066 | 0.0188960572988213 | -1.92894232640239 |
| **RAD51B** | 0.859440929960964 | 4.29689711874123 | 3.00617446721735 | 0.0035704396292169 | 0.0244490104133996 | -1.93724752856511 |
| **NDC80** | 1.67809796089948 | 3.6084823352177 | 2.98488441799906 | 0.00380043240373208 | 0.025184432242243 | -1.94759557926142 |
| **CDC6** | 1.44395578597339 | 6.26393514089737 | 3.06883501360638 | 0.00296629034285234 | 0.0212215199652819 | -1.97702146451408 |
| **WNT7B** | 3.51872473119136 | 1.06533289740239 | 2.96850806365224 | 0.00398660084164382 | 0.0259308049086591 | -1.98150106925754 |
| **PRKACG** | -1.38754134721895 | -0.103805657040987 | -2.97187224048224 | 0.00394768096927666 | 0.0258034783355447 | -2.0000688059311 |
| **SRD5A1** | 1.21104207822223 | 4.4828535134421 | 2.98613646986296 | 0.00378653558486373 | 0.025184432242243 | -2.00344853084469 |
| **PICALM** | 0.576275367730164 | 11.7354884362364 | 3.20855895743843 | 0.00194496287918722 | 0.0158912307969956 | -2.02141732635754 |
| **NFE2L2** | 0.977873728406751 | 5.91553680282495 | 3.03605588755402 | 0.00326935260752163 | 0.0228219856777481 | -2.0247303934294 |
| **SRSF3** | 0.455402191076088 | 6.7750176509881 | 3.06955122539668 | 0.00295997007158016 | 0.0212215199652819 | -2.02516054187619 |
| **IRF7** | -0.708019595551023 | 6.08992988593943 | -3.04000351338882 | 0.00323138827660033 | 0.0227781193223102 | -2.03438168650463 |
| **PLAG1** | -2.39739410693176 | 2.96205707363763 | -2.91873516062149 | 0.00460548853638632 | 0.0286696645685001 | -2.0847656243488 |
| **ISG20** | -0.912286244533581 | 4.87652462090335 | -2.96708028552146 | 0.00400322579257463 | 0.0259308049086591 | -2.0895756566244 |
| **KDM1A** | 0.605825995925193 | 6.54768124845609 | 3.03699644987733 | 0.00326026980927273 | 0.0228219856777481 | -2.09111541341507 |
| **PDIA3** | 0.599478913743603 | 8.53114464026027 | 3.08965110097006 | 0.00278762445317281 | 0.020348243470368 | -2.09633053833146 |
| **AKT3** | 1.11969994445742 | 10.1067870226873 | 3.11728933981082 | 0.002565863935058 | 0.0194195386242811 | -2.14808420311452 |
| **WNT2** | 2.85094535033056 | 4.04999333239555 | 2.90681797919946 | 0.0047662840753316 | 0.0294159506451795 | -2.16648263587176 |
| **HDAC1** | 0.814563115246188 | 4.68008610313991 | 2.9194094517224 | 0.00459654193089168 | 0.0286696645685001 | -2.19007583469095 |
| **NUP98** | 0.411595762735956 | 10.0567816697456 | 3.10047346919577 | 0.00269874409681184 | 0.0199015077498227 | -2.19059848012473 |
| **ZNF703** | -0.751054468721194 | 6.61571275872583 | -3.00013405842977 | 0.00363434608459461 | 0.0246518380643729 | -2.19527730084442 |
| **TMPRSS2** | -2.06015261843754 | 1.2339137065014 | -2.86749835536325 | 0.00533426566551155 | 0.0320953259672848 | -2.20349169788029 |
| **NRAP** | 4.27983013340913 | -0.0558643808349152 | 2.86390255962215 | 0.00538920288233745 | 0.0321563225925363 | -2.23310119699666 |
| **BRIP1** | 0.924397035123963 | 7.80002928805852 | 3.0144820423069 | 0.00348424822923413 | 0.0240882161232629 | -2.24669264107619 |
| **FMOD** | -1.78831753566505 | 7.90805435829958 | -3.01705806285318 | 0.00345791654681393 | 0.0240216618083016 | -2.24696955765673 |
| **GINS2** | 0.947225289582278 | 4.99693762386322 | 2.89561123750788 | 0.00492220426777224 | 0.030119701008751 | -2.28271303776919 |
| **MAGEC1** | 3.39120296711718 | 1.59515937081083 | 2.82754536512314 | 0.00597466621303905 | 0.0346434274772184 | -2.28712456559328 |
| **RAD54L** | 1.22780611130244 | 4.40009483515272 | 2.86445452289851 | 0.00538073618769135 | 0.0321563225925363 | -2.29915467611268 |
| **PTGDS** | -2.1606771406803 | 4.20390008415428 | -2.8573972771429 | 0.00548991710365446 | 0.032621904111798 | -2.29920544053497 |
| **DTL** | 0.984866420721693 | 3.96624427922039 | 2.84927614643137 | 0.00561807780060558 | 0.0331098191691427 | -2.30020700982949 |
| **CDHR1** | -2.60325511798437 | 0.689900263800672 | -2.817693220843 | 0.00614308597265482 | 0.0354769382677817 | -2.32019968169261 |
| **SMAD4** | 0.445674929288453 | 9.95080416962075 | 3.04765212644612 | 0.00315899429644027 | 0.0224882861301045 | -2.32662976436769 |
| **DIRC2** | 0.489107801368794 | 8.06330198086246 | 2.98998510740476 | 0.00374411287620496 | 0.0250420200743383 | -2.32943602692619 |
| **MEG3** | -2.36789385182138 | 5.46196644639608 | -2.87602905491847 | 0.00520598845750329 | 0.0317212347537701 | -2.3840678947477 |
| **CEACAM1** | -1.15384856661421 | 3.02377342420284 | -2.79058444747358 | 0.00662924891620382 | 0.037828809291671 | -2.38626417119892 |
| **MCAM** | -0.7395210477849 | 7.82797755800885 | -2.95456934730061 | 0.00415167087851978 | 0.0267717610910827 | -2.40685996444145 |
| **NUP214** | 0.383373071054937 | 10.9773563156576 | 3.04234059018167 | 0.00320910600184426 | 0.0227324848800593 | -2.41997894138887 |
| **TBC1D10C** | -0.838350415562464 | 5.37647127609931 | -2.85054210397207 | 0.00559792015598104 | 0.0331098191691427 | -2.43782877334046 |
| **PSME1** | 0.665212777305008 | 7.63706827543938 | 2.93286547590958 | 0.00442132694111026 | 0.0280082296974298 | -2.45086679065044 |
| **PIK3R1** | -0.868696595101166 | 10.0002309925012 | -3.00214901721743 | 0.00361291123384085 | 0.0246225893566973 | -2.45298377002289 |
| **GMPS** | 0.736134310014126 | 6.50977090872864 | 2.89668685474936 | 0.00490703757161215 | 0.030119701008751 | -2.45363256857338 |
| **MAFB** | -0.779270775803513 | 7.96666758464597 | -2.93338814337186 | 0.00441464728623057 | 0.0280082296974298 | -2.47158252787519 |
| **KIAA0125** | -1.6140311222143 | 2.57546567298117 | -2.74115463483845 | 0.00760746492464152 | 0.0420751329293635 | -2.48418853926849 |
| **SYCP3** | 2.21650916736716 | -2.17688816400266 | 2.76381681031222 | 0.00714366444167336 | 0.0402846645769658 | -2.48844286713558 |
| **JUN** | -0.719260578422794 | 9.62709941709403 | -2.97634238756601 | 0.00389651036658622 | 0.0257026692988578 | -2.49259168959055 |
| **PLEKHA4** | -0.990449097354285 | 6.35034880804293 | -2.86749337613911 | 0.00533434138121076 | 0.0320953259672848 | -2.5109902613894 |
| **BCAM** | -0.826723536264134 | 5.93073279639023 | -2.84151783685354 | 0.00574307735083536 | 0.0335713220752083 | -2.5270968158759 |
| **RAD51AP1** | 1.15441516852903 | 3.90924645151829 | 2.75053228463808 | 0.00741230285399743 | 0.0411540212511517 | -2.53023148360366 |
| **EGF** | 2.8170765764054 | 1.08348688686692 | 2.71540025860152 | 0.00816785096900644 | 0.0441555251632754 | -2.53323988818836 |
| **NBEA** | -1.90387614394964 | 3.15268652937989 | -2.72380293707688 | 0.00798100779886586 | 0.0438041573082791 | -2.54422364970159 |
| **TTK** | 2.04804269121851 | 4.32760949155221 | 2.75858950874599 | 0.00724828906790212 | 0.0404450416985391 | -2.54743791647965 |
| **SKP1** | 0.748586285593642 | 6.91753203598304 | 2.87146807774748 | 0.00527421466068916 | 0.0320013530889072 | -2.55653167000825 |
| **MALT1** | 0.69962018765289 | 8.73613268595508 | 2.9129802421296 | 0.00468250244055525 | 0.0290234418513726 | -2.58384165122969 |
| **BMF** | -0.701515239147074 | 6.74675352412345 | -2.84746359920386 | 0.00564705494351605 | 0.0331447551378616 | -2.60210765247357 |
| **NRG2** | -2.05222108901016 | 3.05812907595956 | -2.69483692496611 | 0.00864205275860999 | 0.0453550068134349 | -2.60489733433097 |
| **PARP12** | 0.873851382465765 | 4.23223254928642 | 2.7212232563922 | 0.0080379506679996 | 0.0438896505371659 | -2.62643991483794 |
| **ELN** | -2.03587957061992 | 11.6220344356367 | -2.9723951808889 | 0.00394166280208499 | 0.0258034783355447 | -2.66144167345016 |
| **IL11** | 2.92968373994671 | 2.25371806620626 | 2.65485324672041 | 0.00963657677648872 | 0.0485759779235897 | -2.66601211874693 |
| **WIF1** | -3.39894651480548 | -2.28157580928531 | -2.67382472847345 | 0.00915239678422535 | 0.0470040949132716 | -2.66623775347832 |
| **NONO** | 0.349310497353134 | 12.5888330264345 | 2.99831309862434 | 0.00365381856026692 | 0.0246675638012386 | -2.67242434648019 |
| **NKD1** | -1.7404837879964 | 4.52967115004595 | -2.7011839116723 | 0.00849307683365252 | 0.0450665848221119 | -2.7032460343468 |
| **NUTM2A** | -1.26646370021783 | 5.70234210510509 | -2.75818310252351 | 0.00725648175119825 | 0.0404450416985391 | -2.70485622805485 |
| **PDCD1** | -1.28679306561792 | 4.39876905994992 | -2.6867588352693 | 0.00883510402476273 | 0.0458504442959954 | -2.72311243931604 |
| **SMAD9** | -1.21095834337549 | 6.35871847764882 | -2.77920496061968 | 0.00684364200672664 | 0.0387447134081611 | -2.73309514377699 |
| **HMMR** | 2.08041515857325 | 4.8863526961286 | 2.70517440392545 | 0.00840061492682403 | 0.0450665848221119 | -2.73396219253175 |
| **AXL** | 1.15830071584403 | 12.0164245895428 | 2.95134272430674 | 0.00419077292066712 | 0.0269032654460684 | -2.75112461310955 |
| **C1RL** | -0.668668556222687 | 5.03280036865967 | -2.68951687049056 | 0.00876875489368377 | 0.045686483830135 | -2.7881759582603 |
| **S1PR2** | -0.80202928677725 | 7.72345621188695 | -2.79815734565119 | 0.00649000374418024 | 0.0373305015365247 | -2.80111281632265 |
| **CENPA** | 1.1983479035785 | 4.57197321520107 | 2.65634748848119 | 0.00959761330767465 | 0.0485759779235897 | -2.81225729508276 |
| **DTX3L** | 0.518639590451021 | 5.80507355699621 | 2.71896294780935 | 0.00808814839523572 | 0.0438896505371659 | -2.81290144191127 |
| **HDAC8** | 0.727329017266337 | 5.5359016033961 | 2.70187285319281 | 0.00847704771003686 | 0.0450665848221119 | -2.82002958854072 |
| **PLVAP** | -0.764219459198063 | 6.89985855397016 | -2.76152368321804 | 0.00718939012757177 | 0.0403841523572196 | -2.83113534417644 |
| **LY6E** | 0.902357905661092 | 8.03043097194872 | 2.79239423167253 | 0.00659572505122119 | 0.0377874606520162 | -2.83671866852886 |
| **DEK** | 0.61679671298729 | 12.7601135410959 | 2.93753096099066 | 0.00436203129173355 | 0.0278782266556127 | -2.84816426795701 |
| **LMNA** | 0.520811949613471 | 12.4731661659254 | 2.92889735318889 | 0.00447234465072954 | 0.028207156174338 | -2.84832983890064 |
| **KIF5B** | 0.697788734554228 | 12.3779587087822 | 2.92406160793102 | 0.00453525229726307 | 0.0284790078753899 | -2.85340054766333 |
| **CCL14** | -1.23084133789336 | 5.12485143613191 | -2.66447740492703 | 0.00938811778159096 | 0.0477035808124657 | -2.85811514035724 |
| **SCUBE2** | -1.28908116816095 | 5.43767498852039 | -2.67457339577602 | 0.00913375182511437 | 0.0470040949132716 | -2.87272731812452 |
| **GMNN** | 0.79600058064605 | 6.12346557054396 | 2.69756459302717 | 0.00857773976517239 | 0.0452599262803017 | -2.90459375265552 |
| **TET1** | -1.10299522432242 | 7.35462248858777 | -2.73847670868687 | 0.00766405210252155 | 0.0422256970246206 | -2.92384183322712 |
| **CD47** | 0.585399966068079 | 6.24075448525865 | 2.69287598611449 | 0.00868855896077328 | 0.0454332646748799 | -2.930193423975 |
| **CD248** | -0.904725332642988 | 10.5896123146487 | -2.83518991460079 | 0.0058469186580532 | 0.0340399555881802 | -2.93583186319763 |
| **RAD51D** | 0.480736194773715 | 7.02183243654285 | 2.7193350626358 | 0.00807986471362932 | 0.0438896505371659 | -2.9451656240566 |
| **HDAC4** | -0.650709438624883 | 6.16126587912274 | -2.66882947653576 | 0.0092776885931664 | 0.0473096319041606 | -2.97814403353734 |
| **DDX21** | 0.692469633831638 | 5.70929447492488 | 2.64253282646538 | 0.0099633612461387 | 0.0495627165466753 | -2.98285513085174 |
| **ALDH2** | -0.796214067895345 | 6.94313203612925 | -2.66993988936125 | 0.00924970290379556 | 0.0473096319041606 | -3.05822834107074 |
| **TBL1XR1** | 0.488949287819501 | 10.9883791694911 | 2.78656999140017 | 0.00670416959369212 | 0.0381051220384556 | -3.09149998597109 |
| **CCND3** | -0.498783360667474 | 8.57935854761159 | -2.70393022119597 | 0.00842934437688724 | 0.0450665848221119 | -3.09844837875919 |
| **HIST1H3B** | 0.859541168739522 | 7.37606746146405 | 2.653913795907 | 0.00966114720872508 | 0.0485759779235897 | -3.13085359212759 |
| **CCL2** | 1.03732961733387 | 7.22184740386323 | 2.64134982229085 | 0.00999526272499015 | 0.0495627165466753 | -3.14942658670761 |
| **SESN2** | -0.615042433373784 | 7.68355534008214 | -2.63715969104088 | 0.0101090033406353 | 0.0499544563705623 | -3.19270221878287 |
| **ELL** | -0.573201185669051 | 9.38636014243196 | -2.68556380883025 | 0.00886399409894765 | 0.0458504442959954 | -3.2142595569534 |
| **MEF2D** | -0.36278214758077 | 10.7544110824689 | -2.70217247712157 | 0.00847008517766472 | 0.0450665848221119 | -3.28330055935208 |
| **SPARC** | -0.482940003741762 | 11.9467979502059 | -2.69693856111216 | 0.0085924616651755 | 0.0452599262803017 | -3.39707872923289 |
| **B2M** | 0.515988315298396 | 12.8040762661172 | 2.64299899923382 | 0.00995081561704323 | 0.0495627165466753 | -3.60006092800625 |
| **COL3A1** | -0.610563572672532 | 12.8716408911363 | -2.64509957134344 | 0.00989446285514674 | 0.0495627165466753 | -3.6002543446345 |
