# Supplementary material for: Succinate Dehydrogenase B (SDHB) Overexpression with Enzymatic Dysfunction Defines a Distinct Subtype of Undifferentiated Pleomorphic Sarcoma
Source: Cancer Res Commun. 2025 Oct 30;5(10):1934–45. doi: 10.1158/2767-9764.CRC-25-0468 (PMC12573234; doi:10.1158/2767-9764.CRC-25-0468)
Supplement: Supplementary Table 3 [file crc-25-0468_supplementary_table_3_suppst3.docx]

**Supplementary Table 3** - Metabolism-related genes covered by the F1RNA gene set

| ABCA6 | BLVRA | GALNT10 | LDHB | PLCB4 | SRD5A1 |
| --- | --- | --- | --- | --- | --- |
| ABCB1 | CA4 | GALNT12 | MTAP | PLPP3 | ST3GAL2 |
| ABCC2 | CD38 | GMPS | MTHFD1L | POLD1 | SULT1A1 |
| ABCC9 | CES1 | GPI | NAT1 | POLE | SUV39H2 |
| ACACA | CES2 | GSTM1 | NOS1 | PSAT1 | TAP1 |
| ACLY | CMPK2 | GUSB | NSD1 | PTEN | TAP2 |
| ACSL3 | CTPS1 | HDC | NT5C3A | PTGDS | TYMS |
| ACSL6 | CTPS2 | HSD11B1 | NT5E | PTGS2 | UGT8 |
| ADCY7 | DNMT1 | IDO1 | ODC1 | RRM2 | UPP1 |
| ALDH2 | DNMT3A | IMPDH1 | PAFAH1B2 | SCD |  |
| ALOX12 | DOT1L | INPP1 | PHGDH | SCD5 |  |
| ANPEP | ENTPD1 | ITPKB | PIK3CA | SDHA |  |
| ARG2 | FUCA1 | KDSR | PIK3CD | SDHB |  |
| ATIC | FUT4 | KMT5A | PIK3CG | SDHC |  |
| B3GNT5 | FUT8 | KYNU | PLA2G7 | SDHD |  |
