# Supplementary material for: Succinate Dehydrogenase B (SDHB) Overexpression with Enzymatic Dysfunction Defines a Distinct Subtype of Undifferentiated Pleomorphic Sarcoma
Source: Cancer Res Commun. 2025 Oct 30;5(10):1934–45. doi: 10.1158/2767-9764.CRC-25-0468 (PMC12573234; doi:10.1158/2767-9764.CRC-25-0468)
Supplement: Supplementary Table 4 [file crc-25-0468_supplementary_table_4_suppst4.docx]

| **Pathway** | **genes_involved_in_pathway** | **kit_gene_counts** | **leiomyo_lipo_gene_counts** | **UPS_leiomyo_gene_counts** | **UPS_lipo_gene_counts** | **genes_leiomyo_lipo** | **genes_UPS_leiomyo** | **genes_UPS_lipo** | **genes_leiomyo_lipo_commonly_expressed** | **genes_UPS_leiomyo_commonly_expressed** | **genes_UPS_lipo_commonly_expressed** |
| --- | --- | --- | --- | --- | --- | --- | --- | --- | --- | --- | --- |
| KEGG_PURINE_METABOLISM | 159 | 10 | 1 | 0 | 3 | RRM2 | NA | ATIC-GMPS-RRM2 | NA | NA | NA |
| KEGG_OXIDATIVE_PHOSPHORYLATION | 132 | 4 | 0 | 3 | 3 | NA | SDHB-SDHC-SDHD | SDHB-SDHC-SDHD | NA | NA | NA |
| KEGG_PYRIMIDINE_METABOLISM | 98 | 11 | 1 | 0 | 3 | RRM2 | NA | CTPS1-RRM2-TYMS | NA | NA | NA |
| KEGG_GLYCOLYSIS_GLUCONEOGENESIS | 62 | 3 | 0 | 0 | 1 | NA | NA | LDHB | NA | NA | ALDH2 |
| KEGG_ARACHIDONIC_ACID_METABOLISM | 58 | 3 | 0 | 0 | 0 | NA | NA | NA | NA | ALOX12 | PTGDS |
| KEGG_ARGININE_AND_PROLINE_METABOLISM | 54 | 4 | 0 | 0 | 2 | NA | NA | ARG2-ODC1 | NA | NA | ALDH2 |
| KEGG_INOSITOL_PHOSPHATE_METABOLISM | 54 | 7 | 0 | 0 | 0 | NA | NA | NA | NA | NA | ITPKB |
| KEGG_DRUG_METABOLISM_OTHER_ENZYMES | 51 | 7 | 0 | 0 | 1 | NA | NA | GMPS | NA | NA | NA |
| KEGG_GLUTATHIONE_METABOLISM | 50 | 4 | 1 | 0 | 2 | RRM2 | NA | ODC1-RRM2 | NA | NA | NA |
| KEGG_ABC_TRANSPORTERS | 44 | 6 | 0 | 0 | 0 | NA | NA | NA | NA | NA | NA |
| KEGG_LYSINE_DEGRADATION | 44 | 5 | 1 | 0 | 1 | KMT5A | NA | KMT5A | NA | NA | ALDH2 |
| KEGG_FATTY_ACID_METABOLISM | 42 | 3 | 0 | 0 | 0 | NA | NA | NA | NA | NA | ALDH2 |
| KEGG_PYRUVATE_METABOLISM | 40 | 3 | 0 | 0 | 1 | NA | NA | LDHB | NA | NA | ALDH2 |
| KEGG_TRYPTOPHAN_METABOLISM | 40 | 3 | 0 | 0 | 0 | NA | NA | NA | NA | NA | ALDH2 |
| KEGG_SPHINGOLIPID_METABOLISM | 39 | 3 | 0 | 0 | 0 | NA | NA | NA | NA | NA | NA |
| KEGG_CYSTEINE_AND_METHIONINE_METABOLISM | 34 | 4 | 0 | 0 | 1 | NA | NA | LDHB | NA | NA | NA |
| KEGG_ETHER_LIPID_METABOLISM | 33 | 3 | 0 | 0 | 1 | NA | NA | PAFAH1B2 | NA | NA | NA |
| KEGG_PROPANOATE_METABOLISM | 33 | 3 | 0 | 0 | 1 | NA | NA | LDHB | NA | NA | ALDH2 |
| KEGG_CITRATE_CYCLE_TCA_CYCLE | 31 | 5 | 0 | 3 | 3 | NA | SDHB-SDHC-SDHD | SDHB-SDHC-SDHD | NA | NA | NA |
| KEGG_O_GLYCAN_BIOSYNTHESIS | 30 | 3 | 0 | 0 | 0 | NA | NA | NA | NA | NA | NA |
| KEGG_NICOTINATE_AND_NICOTINAMIDE_METABOLISM | 24 | 3 | 0 | 0 | 0 | NA | NA | NA | NA | NA | NA |
| KEGG_ONE_CARBON_POOL_BY_FOLATE | 17 | 3 | 0 | 0 | 2 | NA | NA | ATIC-TYMS | NA | NA | NA |

**Supplementary Table 4** - Distribution of metabolism-related genes (covered by the F1RNA gene set) per metabolic pathway
