# Supplementary material for: Succinate Dehydrogenase B (SDHB) Overexpression with Enzymatic Dysfunction Defines a Distinct Subtype of Undifferentiated Pleomorphic Sarcoma
Source: Cancer Res Commun. 2025 Oct 30;5(10):1934–45. doi: 10.1158/2767-9764.CRC-25-0468 (PMC12573234; doi:10.1158/2767-9764.CRC-25-0468)
Supplement: Supplementary Table 7 [file crc-25-0468_supplementary_table_7_suppst7.docx]

**Supplementary Table 7 -** Demographic, pathological and clinical characteristics of the patients whose fresh normal and tumor tissue samples were used for ^1^H NMR

|  | **Total**  (n=16) |
| --- | --- |
| **Age at diagnosis**, median [IQR], years  **Gender**, n (%)  Male  Female  **Histologic subtypes**, n (%)  Undifferentiated pleomorphic sarcoma  Leiomyosarcoma  Liposarcoma  Other histology*  **Tumor grade**, n (%)  G1  G2  G3  **Location**, n (%)  Upper limb  Lower limb  Retroperitoneum  Abdominal/Pelvic  Trunk  **Presentation type**, n (%)  Localized  Distant metastasis | 74 [33.4  5 (31.2)  11 (68.8)  4 (25.0)  3 (18.8)  2 (12.5)  7 (43.7)  2 (12.5)  5 (31.2)  9 (56.3)  3 (18.8)  7 (43.7)  3 (18.8)  2 (12.5)  1 (6.2)  13 (81.2)  3 (18.8) |

*1 DFSP, 1 SFT, 1 synovial sarcoma,1 chondrosarcoma,1 CCSLGT, 1 low-grade endometrial stromal sarcoma, and 1 adamantinoma
