# Supplementary material for: Succinate Dehydrogenase B (SDHB) Overexpression with Enzymatic Dysfunction Defines a Distinct Subtype of Undifferentiated Pleomorphic Sarcoma
Source: Cancer Res Commun. 2025 Oct 30;5(10):1934–45. doi: 10.1158/2767-9764.CRC-25-0468 (PMC12573234; doi:10.1158/2767-9764.CRC-25-0468)
Supplement: Supplementary Table 8 [file crc-25-0468_supplementary_table_8_suppst8.docx]

**Supplementary Table 8 -** Surgical and systemic treatment specificities of the patients that underwent curative-intent surgery at the participating institutions and whose fresh normal and tumor tissue samples were used for ^1^H NMR

|  | **Total**  (n=13) |
| --- | --- |
| **Neoadjuvant treatment,** n (%)  No neoadjuvant treatment  Neoadjuvant treatment  **Ressectability,** n (%)  Resectable  **Ressection margins,** n (%)  R0/R1  **Adjuvant treatment,** n (%)  Radiotherapy  Chemotherapy  No adjuvant treatment | 9 (69.2)  4 (30.8)  13 (100.0)  13 (100.0)  4 (30.8)  1 (7.7)  8 (61.5) |
